# SnCl_4_-catalyzed solvent-free acetolysis of 2,7-anhydrosialic acid derivatives

**DOI:** 10.3762/bjoc.15.295

**Published:** 2019-12-23

**Authors:** Kesatebrhan Haile Asressu, Cheng-Chung Wang

**Affiliations:** 1 Institute of Chemistry, Academia Sinica, Taipei 115, Taiwan; 2 Taiwan International Graduate Program (TIGP), Sustainable Chemical Science and Technology (SCST), Academia Sinica, Taipei 115, Taiwan; 3 Department of Applied Chemistry, National Chiao Tung University, Hsinchu 300, Taiwan

**Keywords:** acetolysis, acetolysis products, 2,7-anhydrosialic acid, SnCl_4_

## Abstract

Sialic acid-containing glycans are found in different sialic acid forms and a variety of glycosidic linkages in biologically active glycoconjugates. Hence, the preparation of suitably protected sialyl building blocks requires high attention in order to access glycans in a pure form. In line with this, various C-5-substituted 2,7-anhydrosialic acid derivatives bearing both electron-donating and -withdrawing protecting groups were synthesized and subjected to different Lewis acid-catalyzed solvent-free ring-opening reactions at room temperature in the presence of acetic anhydride. Among the various Lewis acids tested, the desired acetolysis products were obtained in moderate yields under tin(IV) chloride catalysis. Our methodology could be extended to regioselective protecting group installations and manipulations towards a number of thiosialoside and halide donors.

## Introduction

Sialic acids are the most prevalent monosaccharides that are found at the nonreducing ends of glycans, and they are involved in many biologically important ligand–receptor interactions [1]. *N*-Acetylneuraminic acid (Neu5Ac) is the most studied monosaccharide from the 50 derivatives of sialic acid that are found in nature. The most common glycosidic linkages of Neu5Ac in glycoconjugates are α(2→3) and α(2→6) to galactose, α(2→8) and α(2→9) in polysialic acids [2–3], fucosyl and galactosyl α(1→4) to Neu5Ac, and intramolecularly C-7-to-C-2-linked (via oxygen), forming 2,7-anhydro-Neu5Ac [4–6]. Owing to the substantial role of sialic acids in biological systems, numerous synthetic methods have been described in the literature [7–16]. 2,7-Anhydro-Neu5Ac is the main prebiotic, which is utilized as a sole carbon source for human gut commensal anaerobic bacterium and plays a key biological role in body fluids and secretion [17–19].

**Figure 1:**
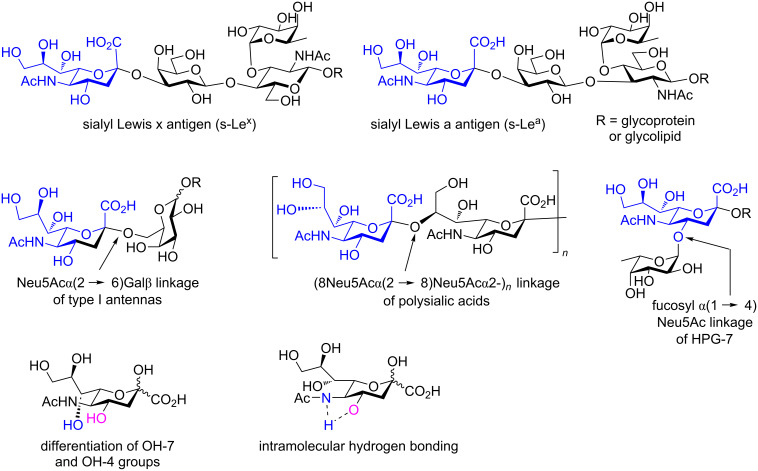
Representative structures of bacterial glycans containing sialic acid.

Upon cleaving the internal ketal ring and protection of the OH-4 group, further modifications at carbon C-7 and sialylations at the C-2 position can be conducted. To study substrate specificity of bacterial and human sialidases using the library of position C-7-modified Neu5Ac, Chen and co-workers have described the systematic substitution of the OH-7 moiety of Neu5Ac by F, OMe, H, and N_3_ substituents [20]. Additionally, De Meo and co-workers have reported the significant effect of *O*-substituents on atom C-7 on the reactivity of thiosialoside donors and on the anomeric stereoselectivity of chemical sialylations [21]. Thus, 2,7-anhydro-Neu5Ac can serve as a promising building block for the chemical synthesis of gangliosides and other Neu5Ac-containing glycans after its 2,7-anhydro backbone has been opened. Representative structures of bacterial glycans containing sialic acid, which can be chemically synthesized from sialyl donors, are illustrated in Figure 1 [2–5,22–24].

One way to obtain Neu5Ac-containing oligosaccharides with the desired regioselectivity is to regioselectively protect the Neu5Ac acceptor. Although Neu5Ac with a free primary OH-9 function is easy to obtain, there are only limited ways to distinguish the secondary OH-4 and OH-7 group (Figure 1). In our previous report, we easily distinguished these alcohols by selective protection of the OH-7 functionality as 2,7-anhydro-Neu5Ac, leaving position OH-4 free for glycosylation upon protection of the vicinal OH-8 and OH-9 substituents as benzylidene or acetonide rings [6]. The formation of the bicyclo[3.2.1] backbone of 2,7-anhydro-Neu5Ac prevented not only the installation of protection groups at the C-2 and C-7 position, but also circumvented the tedious purification of anomeric isomers, as this gave only the α-isomer. Besides, the configuration of the alcohol OH-4 and the NHAc group in position C-5 changed from equatorial to axial after 2,7-anhydro backbone formation, which resulted in alleviation of hydrogen bonding, and thus enhancing the reactivity of the OH-4 group as an acceptor (Figure 1) [6].

Several chemical and enzymatic syntheses of 2,7-anhydro-Neu5Ac are currently being developed [6,25–28]. However, only few synthetic applications of 2,7-anhydro-Neu5Ac are available in the literature. Previously, we successfully constructed the fucosyl α(1→4) to Neu5Ac linkage of ganglioside HPG-7 using 2,7-anhydro-Neu5Ac derivatives as acceptors [6]. For utilization as selective sialidase inhibitors, Chen’s and Juge’s groups have reported one-pot multienzyme synthetic protocols for 2,7-anhydro-Neu5Ac [17–19].

Our group has developed one-pot syntheses of several anhydro sugars via microwave (MW)-assisted intramolecular anomeric protection (iMAP) of silylated sugars as well as ring-opening protocols for their 1,6-anhydro bridges [29–31]. Accordingly, ᴅ-galactosamine and ᴅ‑allosamine derivatives were synthesized via scandium(III) triflate-catalyzed ring opening of 1,6-anhydroglucosamine derivatives [29,32]. However, there is a paucity of reports on the Lewis acid-catalyzed acetolysis of the 2,7-anhydro backbone of Neu5Ac. Thus, the aim of this study was to cleave the ring of the 2,7-anhydro derivatives with a choice of suitable Lewis acids as catalysts in order to utilize the acetolysis products as building blocks for the chemical synthesis of Neu5Ac-containing glycans.

## Results and Discussion

It is known that Sc(OTf)_3_ [29,32] and trimethylsilyl trifluoromethanesulfonate [33] in the presence of acetic anhydride act as acetolysis agents to cleave the 1,6-anhydro ring. In these reactions, Ac_2_O is used as both reactant and solvent, which can be easily removed in vacuo. Envisioned on these precedents, we applied an acetolysis procedure as a way to introduce acetate to the C-2 and C-7 position of Neu5Ac in order to prepare regioselectively protected glycosyl donors.

**Scheme 1:**
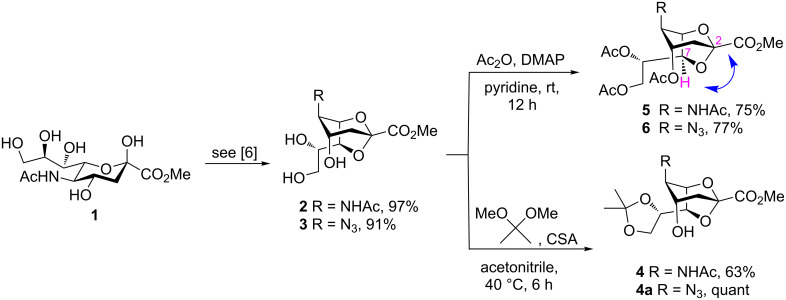
Concise synthesis of 2,7-anhydrosialic acid derivatives **2**–**6**. Conditions for the preparation of **2** and **3**, respectively, from **1** have been reported previously [6].

Our investigations began with the synthesis of 2,7-anhydro derivatives **2** and **3** following our previously published procedure [6]. In order to characterize the formation of the 2,7-anhydro skeleton, triols **2** and **3** were treated with 2,2-dimethoxypropane in the presence of a catalytic amount of camphorsulfonic acid (CSA) in acetonitrile to afford compound **4** in 63% and azide acceptor **4a** in quantitative yield (Scheme 1).

**Figure 2:**
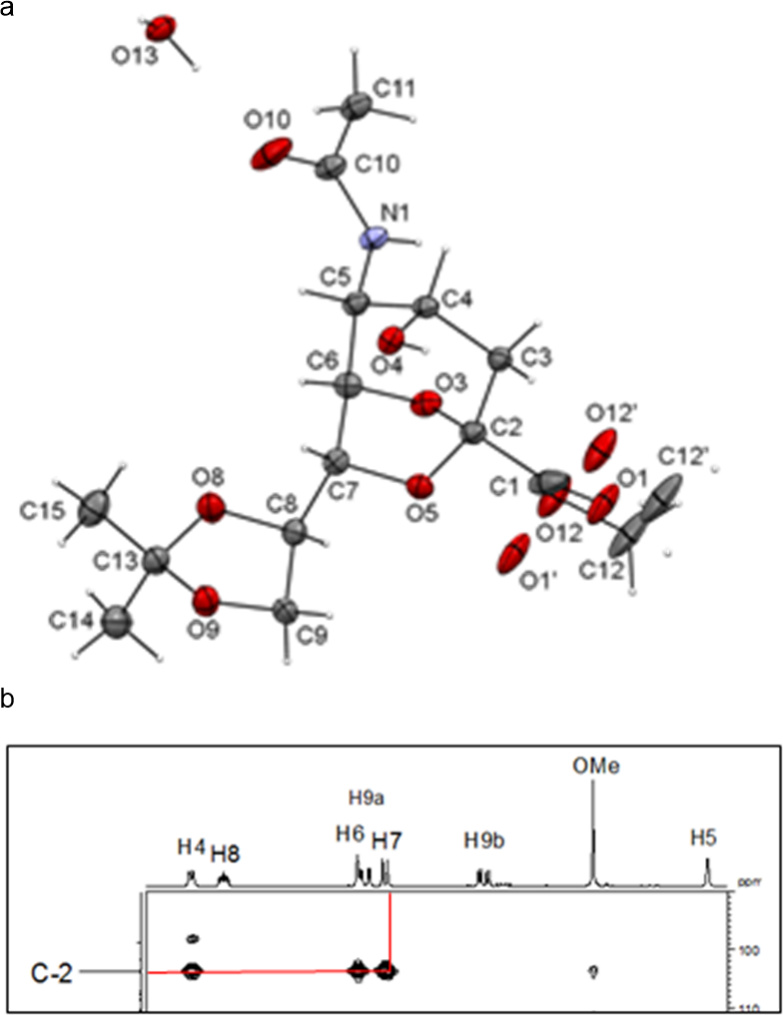
a) ORTEP diagram of compound **4**. Thermal ellipsoids indicate 50% probability. b) HMBC spectrum of **6**.

The crystal structure of **4** clearly showed that the NHAc group in position C-5 and the OH-4 group were oriented in a *trans-*diaxial configuration (Figure 2a). This enhanced the nucleophilicity of the OH-4 group due to the absence of hydrogen bonding between these moieties. After confirming the structure of **4** by single crystal X-ray crystallography, compounds **2** and **3** were acetylated with Ac_2_O in the presence of a catalytic amount of 4-(dimethylamino)pyridine (DMAP) in pyridine to give **5** [6] and **6** in 75 and 77% yield, respectively (Scheme 1), and the compatibility of acetyl protecting groups with the Lewis acids during acetolysis reactions was tested. The structure of compound **6** was confirmed by NMR analysis. The HMBC spectrum of **6** showed downfield shift of the atom H-4. Hydrogen H-7 illustrated a correlation separated by three bonds with atom C-2, like that with hydrogens H-4 and H-6 (Figure 2b).

**Scheme 2:**
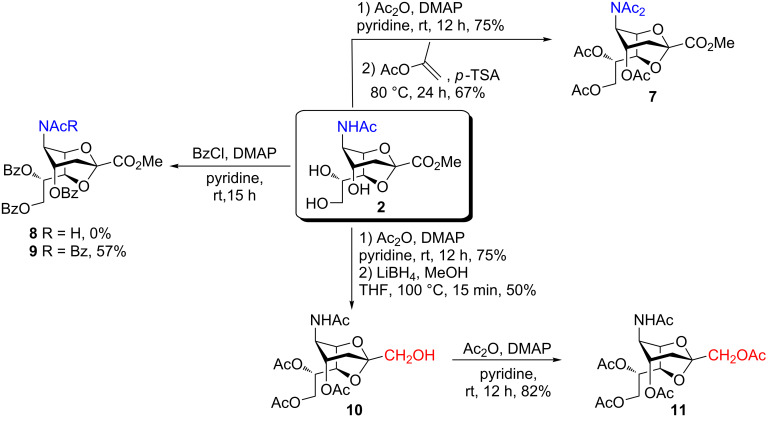
N- and C-1-functionalization of **2**.

Furthermore, the C-1 and C-5 positions of compound **2** were functionalized in certain steps to open the 2,7-anhydro bridge. Accordingly, an additional *N*-acetyl moiety was introduced to **5** following the protocol developed by Boons and Demchenko by using isopropenyl acetate and *p*-TSA, which afforded **7** in 67% yield. On the other hand, per-*O*-benzoylation of **2** with benzoyl chloride in pyridine produced compound **9** in a moderate yield of 57% (Scheme 2) [34].

Wong and co-workers reported the major effects of the carboxy group on the anomeric reactivity of sialic acid compared with the side chain protecting groups. Hence, we converted **2** into *O*-acetyl-protected hydroxymethyl group-substituted compound **11** in order to increase its reactivity by reducing the steric hinderance and destabilizing effect of the electron-withdrawing carboxy group at the anomeric center (Scheme 2) [35]. This included a LiBH_4_ reduction in MeOH and THF, followed by acetylation of the hydroxymethyl group in **10** to furnish **11** in 82% yield.

After having obtained the 2,7-anhydrosialic acid derivatives **5**–**11**, they were subjected to various Lewis acid-catalyzed ring-opening reactions. In line with this, Sc(OTf)_3_-, copper(II) triflate-, and SnCl_4_-catalyzed acetolysis of diacetylamino and acetyl(benzyl)amino derivatives (**7** and **9**) did not afford the desired acetolysis products. In the course of these reactions, the functional diacetylamino and acetyl(benzyl)amino groups in **7** and **9**, respectively, were hydrolyzed to NHAc functionalities, giving **5** and **8**, respectively, but with compound **8** only affording an unidentifiable mixture of compounds. Similarly, the 2,7-anhydro backbone of **11** was not cleaved under the above conditions and results in either recovered starting material or unidentifiable mixtures. Differently, ring-opening reactions of **6** with BF_3_⋅OEt_2_, TMSOTf, and Sc(OTf)_3_ in Ac_2_O afforded glycal **13** (Table 1, entries 1–4). None of these conditions were successful for the acetolysis of these 2,7-anhydro derivatives, although they worked for the 1,6-anhydro sugars [29,32–33]. Adding 10 equiv of acetic acid to suppress the 2,3-elimination reaction failed to give the desired compound **12**. Due to the absence of a hydrogen bond, azido-protected glycal **13** could be used as acceptor for reactions at its OH-4 and OH-8 group [36–37]. Moreover, it was used as sialyl donor in α-selective glycosylation reactions [35].

**Table 1:** Optimization of the ring-opening reaction of **6**.

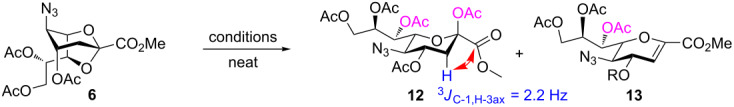					

entry	conditions	*T* (^o^C)	*t* (h)	product, yield	

				**12**	**13**

1	BF_3_⋅OEt_2_ (50 mol %), Ac_2_O (30 equiv)	100	19	–	15%^a^
2	TMSOTf (50 mol %), Ac_2_O (30 equiv)	70	20	–	13%^a^
3	TMSOTf (50 mol %), Ac_2_O (10 equiv)	70	11	–	20%^a^
4	Sc(OTf)_3_ (50 mol %), Ac_2_O (30 equiv)	100	2	–	7%^a^
5	SnCl_4_ (50 mol %), Ac_2_O (30 equiv)	rt	20	54%	34%

^a^Incomplete consumption of starting material.

Next, acetolysis of 2,7-anhydro derivative **6** was examined with SnCl_4_ as catalyst under neat conditions, which provided the expected β-ring-opened product **12** in a moderate yield (Table 1, entry 5). This acetolysis reaction resulted in regioselective acetylation of positions O*-*7 and O-2 in line with the principles of green chemistry.

It is known that anomeric acetates can be transformed smoothly into thioglycoside [15–16,35] and sialyl halide [38–39] donors when treated with a thiol in the presence of triflic acid, BF_3_⋅OEt_2_, and HCl, respectively.

**Scheme 3:**
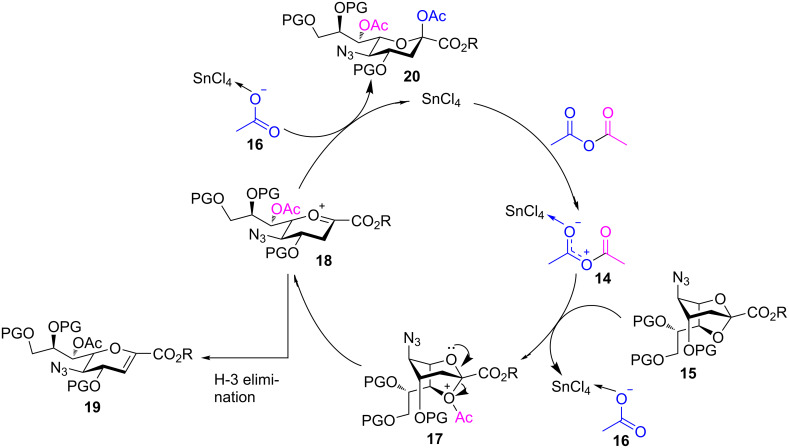
Mechanism of the SnCl_4_-catalyzed acetolysis of 2,7-anhydro derivatives **15**. R = Me, Bn, PG = electron-donating protecting groups, electron-withdrawing protecting groups.

Through comparison with the reported mechanism of Sc(OTf)_3_-catalyzed acetolysis of 1,6-anhydro-β-hexopyranoses [32], we propose a plausible mechanism for the acetolysis of 2,7-anhydro-Neu5N_3_ derivatives **15** (Scheme 3): First, Ac_2_O is activated with SnCl_4_ forming a polarized complex **14**. Next, the O-7 atom of **15** reacts with the acylium group in **14** to afford an unstable species **17** and its counter ion **16**. The C-2–O-7 bond of the positively charged intermediate **17** rapidly undergoes bond cleavage to generate the sialyl cation **18**, which is then attacked by nucleophile **16** from the β-face to provide the desired acetolysis product **20** and the original SnCl_4_ species. On the other hand, abstraction of hydrogen H-3 by the nucleophile **16** affords the 2,3-elimination product **19**.

**Scheme 4:**
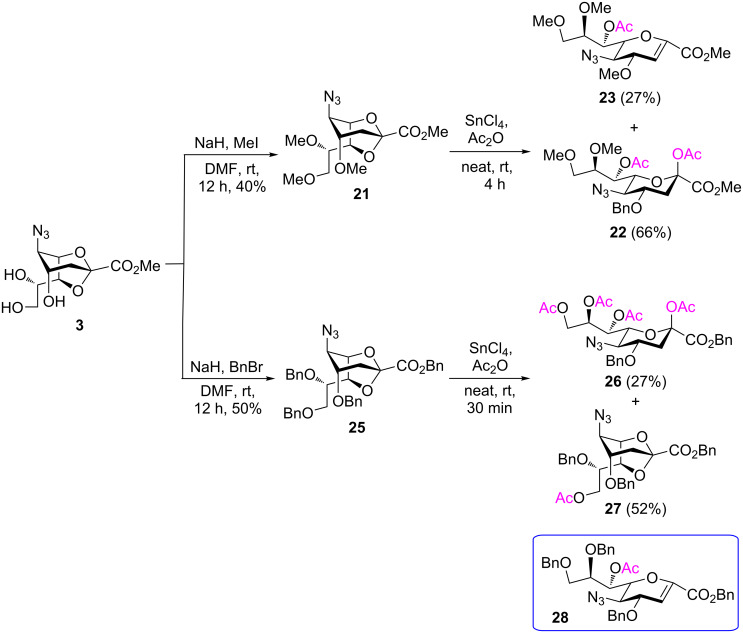
Synthesis and acetolysis of 2,7-anhydro derivatives **21** and **25**.

To substantiate the reaction mechanism and expand the substrate scope, we synthesized derivatives **21** and **25** through alkylation of **3** with MeI/NaH and BnBr/NaH, respectively, in DMF, following the reported procedures [35], which afforded the trimethylated species **21** (40%) and the tribenzylated compound **25** (50%), respectively (Scheme 4). Thus, the reactive 2,7-anhydro compounds **21** and **25**, which carried electron-donating groups, were examined under the established optimized ring-opening conditions.

As expected, the acetolysis proceeded well under the optimized conditions and gave the desired acetolysis products **22** and **26**. While ring opening of the triacetylated **6** and trimethylated **21** were accompanied by competitive 2,3-elimination, yielding products **13** (34%) and **23** (27%), respectively (Table 2, entries 1 and 2), the formation of glycal **28** from substate **25** was suppressed under the same conditions (Table 2, entry 3). The SnCl_4_-catalyzed acetolysis of tribenzylated **25** offered the acetolysis product **26** in a low yield (27%) and occurred faster than with **6** and **21**. The fast ring-opening reaction of **25** might have been attributed to the presence of three highly electron-donating benzyl groups. The low yield of **26** occurred because the more reactive primary benzyl group of **25** was cleaved [40] and replaced with an acetyl protecting group, resulting in **27** in 52% yield.

**Table 2:** Substantiation of the reaction mechanism.

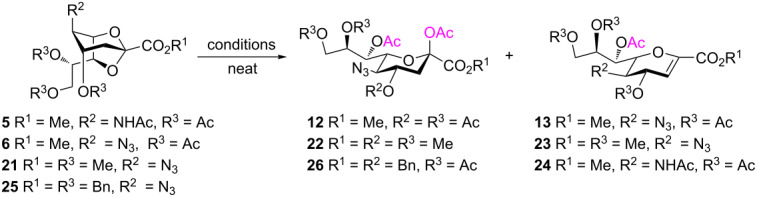			

entry	donor	ring-opening product	glycal

1	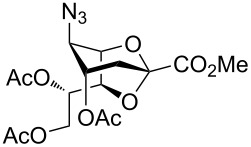 **6**	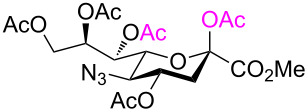 **12** (54%)	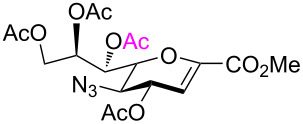 **13** (34%)
2	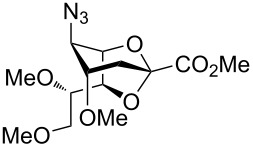 **21**	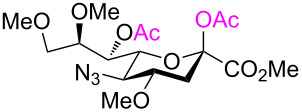 **22** (66%)	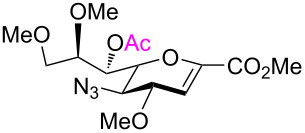 **23** (27)
3	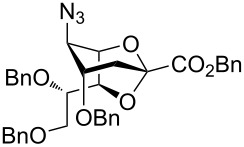 **25**	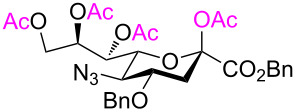 **26** (27%)	–
4	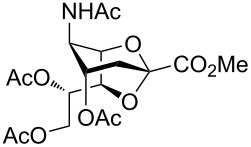 **5**	–	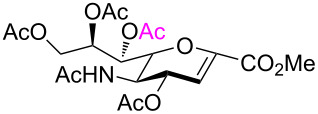 **24** (44%)

**Figure 3:**
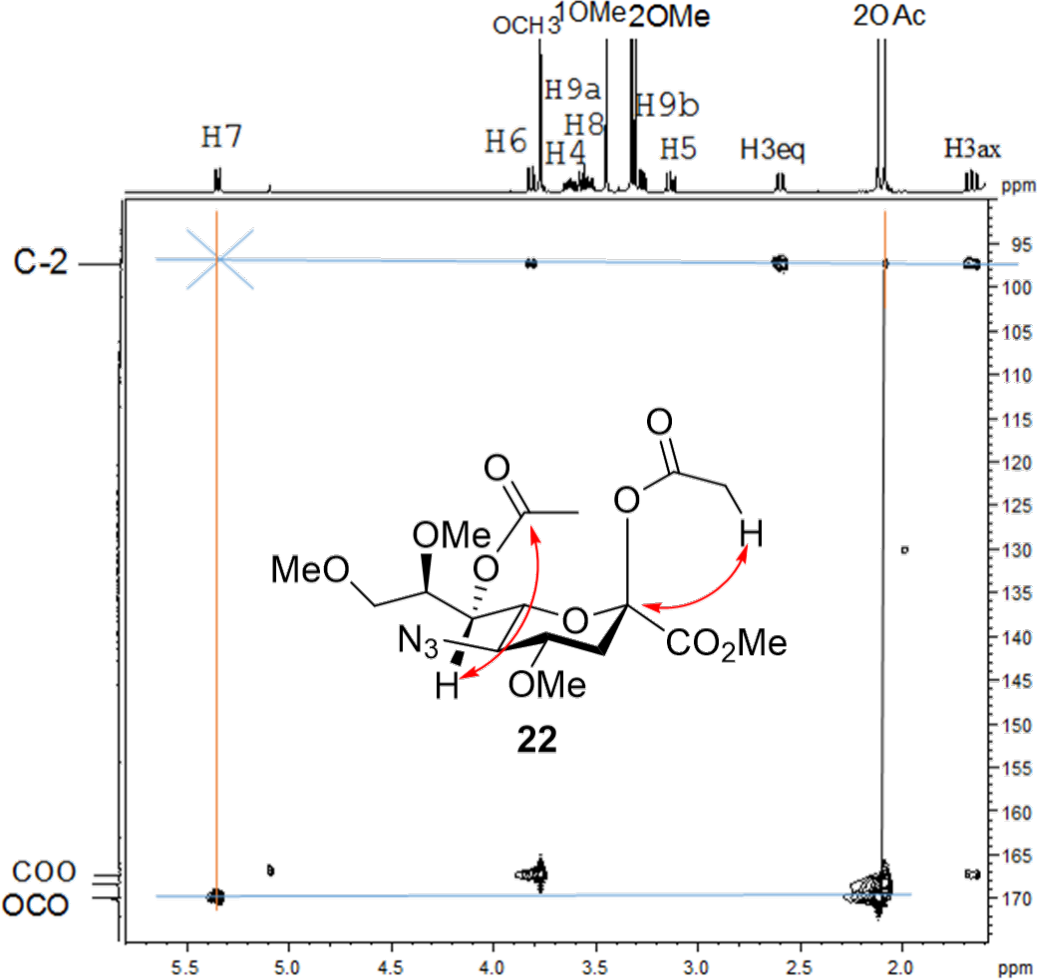
HMBC spectrum of carbohydrate **22**.

Cleavage of the intramolecular glycosidic bond in **5**, **21**, and **25** was confirmed by NMR spectroscopic analysis. For example, position H-7 of compound **22** shifted downfield due to the presence of an acetyl group on oxygen atom O-7, which was introduced after cleavage of the 2,7-anhydro skeleton. NMR also showed no correlation with position C-2, but exhibited a correlation separated by three bonds with the quaternary carbon atom of the acetyl group in position O-7 (Figure 3). The anomeric configuration of the acetolysis products was identified by the coupling constant for the correlation C-1–C-2–C-3–H-3_ax_ (
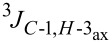
 = 1.6–2.2 Hz, see Supporting Information File 1) and indicated the β-anomer [12–16]. Moreover, the similar chemical shifts of positions H-3_eq_ and H-3_ax_ in the ^1^H NMR spectra of α-sialosides **6**, **21**, and **25** became much more different for the ring-opening products **12**, **22**, and **26**, again representative of the β-configuration.

Next, SnCl_4_-catalyzed acetolysis was evaluated with 2,7-anhydro derivative **5**; however, the reaction did not take place. On the other hand, only a known glycal **24** was obtained in a moderate yield of 44% by Sn(OTf)_2_ catalysis (Table 2, entry 4). The driving force for the formation of a 2,3-dehydro derivative **24** might have been attributed to the presence of a destabilizing electron-withdrawing carboxy group flanking atom C-2 and the lack of a participating auxiliary in position C-3 of **5**. Glycal **24** is widely used for α-sialylation by utilizing neighboring group participation and site-selective fluorination at C-3 [12–14,41]. Moreover, it is the main precursor in the synthesis of inhibitors for *N-*acetylneuraminidases from different sources [42]. The ring opening of **5** was not trivial since the substrate had a sterically hindered quaternary anomeric center, unlike the tertiary anomeric center C-1 of 1,6-anhydro sugars [29–33,43]. Furthermore, SnCl_4_ and Sn(OTf)_2_ might have formed a coordination bond with the carbonyl oxygen atom of the C-5 NHAc group and the O-6 position of the glucose glucopyranosyl ring. In turn, this retarded the ring-opening reaction. The anchimeric participation of the NHAc group in stabilizing the axial conformation of the sialyl cation could also have affected cleavage of the ring [12–14].

**Scheme 5:**
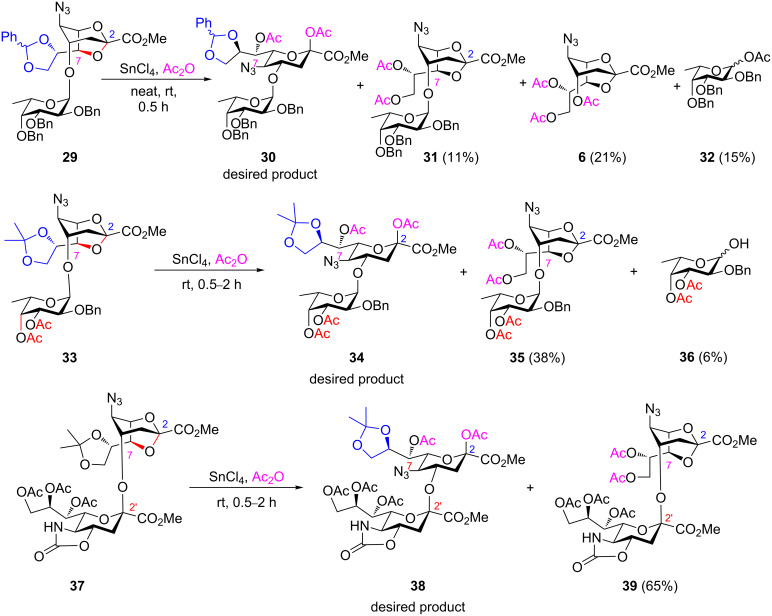
Attempted acetolysis of 2,7-anhydro-NeuN_3_-based disaccharides **29**, **33**, and **37**.

To further test the scope of the optimized ring-opening conditions, disaccharides **29**, **33**, and **37**, bearing a 2,7-anhydro-Neu5N_3_ unit, have been synthesized based on our reported procedure [6] and were examined as substrates (Scheme 5). However, the desired acetolysis products **30**, **34**, and **38** were not obtained. Replacing the more reactive fucose moiety and the benzylidene acetal ring of **29**, i.e., using the less reactive complement **33**, was also unsuccessful. The branched fucose, benzylidene, and isopropylidene rings of the disaccharides were cleaved and acetylated, as shown in Scheme 5. The difficulty of these reactions might have been attributed to the more liable nature of the tertiary acetal center C-1 of fucose as compared to the sterically hindered quaternary ketal functionality C-2 of the 2,7-anhydro skeleton. To our disappointment, an attempted acetolysis upon changing the fucose-containing disaccharides **29** and **33** with substrate **37** did not provide the desired ring-opening product **38** albeit it bore a robust N-5/O-4-oxazolidinone-based sialyl group (Scheme 5). Furthermore, 8,9-di-*O*-acetylated disaccharide **39** was subjected to SnCl_4_-catalyzed acetolysis, but the reaction did not proceed even under prolonged reaction time, which might have been attributed to the bulkier anomeric center C-2 in the 2,7-anhydro bridge.

## Conclusion

In conclusion, an acetolysis reaction was carried out by considering various parameters, such as Lewis acid type and amount (both catalytic and stoichiometric), solvent, acetolysis agent, reaction time, and temperature. However, these conditions were unsuccessful using 2,7-anhydro-based monosaccharide derivatives bearing NHAc, NAc_2_, and NAcBz functionalities and the disaccharides containing a 2,7-anhydro-Neu5N_3_ unit. Meanwhile, we successfully developed a convenient route to conduct the SnCl_4_-catalyzed acetolysis of 2,7-anhydro-Neu5N_3_ monosaccharide derivatives functionalized with a variety of protecting groups. The acetolysis products could further be transformed into regioselectively protected thiosialoside and sialyl halide donors in order to serve as alternative building blocks for the synthesis of sialic acid-containing glycans.

## Supporting Information

File 1 Experimental section and spectroscopic data for compounds described herein.

File 2 X-ray crystallographic data for compound **4**.
