# Supplementary material for: SnCl4-catalyzed solvent-free acetolysis of 2,7-anhydrosialic acid derivatives
Source: Beilstein J Org Chem. 2019 Dec 23;15:2990–9. doi: 10.3762/bjoc.15.295 (PMC6948141; doi:10.3762/bjoc.15.295)
Supplement: File 1 — Experimental section and spectroscopic data for compounds described herein. [file Beilstein_J_Org_Chem-15-2990-s001.pdf]

BEILSTEIN JOURNAL OF ORGANIC CHEMISTRY

Supporting Information

for

$SnCl_4$-catalyzed solvent-free acetolysis of 2,7-anhydrosialic acid derivatives

Kesatebrhan Haile Asressu and Cheng-Chung Wang

Beilstein J. Org. Chem. **2019,** *15,* 2990–2999. doi:10.3762/bjoc.15.295

Experimental section and spectroscopic data for compounds described herein

License and Terms: This is a supporting information file under the terms of the Creative Commons Attribution License (https://creativecommons.org/licenses/by/4.0). Please note that the reuse, redistribution and reproduction in particular requires that the authors and source are credited.
The license is subject to the *Beilstein Journal of Organic Chemistry* terms and conditions: (https://www.beilstein-journals.org/bjoc)

Table of contents

	page
Experimental section	S2
NMR spectra	S22
References	S87

Experimental section

General information. All dry solvents and chemicals were purchased from commercial sources, and were used without further purification unless otherwise mentioned. All moisture-sensitive reactions were conducted in flame-dried glassware under a dry nitrogen atmosphere. Flash column chromatography was carried out as recommended with Silica Gel 60 (230–400 mesh, Merck). TLC was performed on precoated glass plates of Silica Gel 60 F_{254} (0.25 mm, E. Merck); detection was executed by UV (254 nm) or spraying with a solution of $Ce(NH_4)_2(NO_3)_6$, $(NH_4)_6M_7O_{24}$, as well as H_2SO_4 in water and subsequent heating on a hot plate. Specific rotations were measured on a Jasco P-2000 digital polarimeter using a 100 mm cell at 589 nm and at ambient temperature conditions and reported in $10^{-1}\cdot deg\cdot cm^2\cdot g^{-1}$; the sample concentrations are given in $g\cdot dL^{-1}$. ^{1}H and ^{13}C NMR spectra were recorded with Bruker AVIII-400, AV500 or N600 MHz instruments. Chemical shifts are in ppm from Me_4Si, generated from the $CDCl_3$ lock signals at δ 7.24 for ^{1}H spectra and 77.16 for ^{13}C spectra, respectively. Multiplicities are reported by using the following abbreviations: s = singlet, d = doublet, t = triplet, q = quartet, m = multiplet, br = broad; *J* = coupling constant values in Hertz. Proton peaks were assigned based on 2D NMR spectra (COSY, HSQC, HMBC, and NOESY). Mass spectra were obtained with a JEOL JMS-700 mass spectrometer in FAB mode or Waters Premier XE mass spectrometer in ESI mode. IR spectra were taken with a Perkin-Elmer Paragon 1000 FT-IR spectrometer.

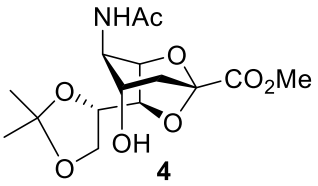

4

Methyl 5-acetamido-2,7-anhydro-3,5-dideoxy-8,9-*O*-isopropylidene-D-glycero-α-D-galacto-2-nonulopyranosidonate (4): To a solution of **2** in CH_3CN, 2,2-dimethoxypropane (625 µL, 5.1 mmol, 3 equiv) and camphorsulfonic acid (80 mg, 0.33 mmol, 0.2 equiv) were added at room temperature and stirred for 8 h. When reaction was completed, the mixture neutralized with trimethylamine (Et_3N) and concentrated in vacuo. The crude product was purified by column chromatography (60% EtOAc in hexane) to afford the desired product **4** as pale yellow solid (366.2 mg, 63% yield). $[\alpha]^{28}_D$ +6 (c = 1.1, $CHCl_3$); mp 150.6-152 ^oC; IR ($CHCl_3$) *v* 3312, 2987, 1752, 1655, 1540, 1440, 1373, 1265, 1206, 1157, 1088, 1073, 1051, 840, 742 cm^{-1}; ^1H NMR (500 MHz, $CDCl_3$): δ = 6.15 (d, *J* = 8.5 Hz, 1H, NH), 4.55 (s, 1H, H-6), 4.50 (d, *J* = 8.8 Hz, 1H, H-7), 4.10-4.05 (overlap, 2H, H-5, H-9a), 3.96-3.89 (overlap, 3H, H-4, H-8, H-9b), 3.81 (s, 3H, OCH_3), 3.41 (d, *J* = 4.9 Hz, 1H, 4-OH), 2.14 (d, *J* = 3.4 Hz, 2H, H-3eq, H-3ax), 1.99 (s, 3H, NAc), 1.38, 1.29 (each s, 6H, CH_3) ppm; ^{13}C NMR (125 MHz, $CDCl_3$): δ = 170.0 (CO), 167.3 (CO), 110.0 (C), 104.2 (C-2), 79.0 (CH), 78.3 (CH), 75.3 (CH), 67.3 (CH_2), 67.1 (CH), 53.3 (OCH_3), 52.0 (CH), 35.8 (CH_2), 27.1 (CH_3), 25.4 (CH_3), 23.4 (CH_3); HRMS (ESI): *m/z* calcd for $C_{15}H_{23}NO_8$ ($[M+Na]^+$): 368.1321, found: 368.1317.

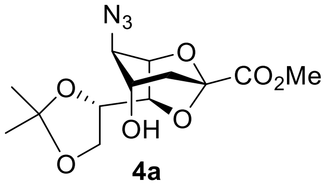

4a

Methyl 2,7-anhydro-5-azido-3,5-dideoxy-8,9-*O*-isopropylidene-ᴅ-glycero-α-ᴅ-galacto-2-nonulopyranosidonate (4a): To a solution of **3** (176 mg, 0.61 mmol, 1 equiv in 5 mL CH_3CN), 2,2-dimethoxypropane (224 µL, 1.83 mmol, 3 equiv) and CSA (21 mg, 0.09 mmol, 0.2 equiv) were added at rt and stirred for 2 h at the same temperature. When the reaction was complete, the mixture neutralized with Et_3N and concentrated in vacuo. The crude product was purified by column chromatography (30% EtOAc in hexane) to afford the desired product **4a** as pale yellow compound (201.2 mg, quant.). $[\alpha]^{28}_{D}$ +40.42 (c = 0.58, $CHCl_3$); IR ($CHCl_3$) *v* 3481, 2987, 2102, 1753, 1440, 1373, 1261, 1155, 1096, 1072, 1049, 841,741 cm^{-1}; ^{1}H NMR (500 MHz, $CDCl_3$): δ = 4.76 (br s, 1H, H-6), 4.29 (d, *J* = 9.4 Hz, 1H, H-7), 4.10-4.03 (overlap, 2H, H-4, H-9a), 4.01-3.98 (m, 1H, H-8), 3.93 (dd, *J* = 4.5, 8.8 Hz, 1H, H-9b), 3.81 (s, 3H, OCH_3), 3.54 (br d, *J* = 1.2 Hz, 1H, H-5), 2.58 (d, *J* = 7.0 Hz, 1H, 4-OH), 2.32 (dd, *J* = 5.3, 15.4 Hz, 1H, H-3eq), 2.15 (d, *J* = 15.2 Hz, 1H, H-3ax), 1.40, 1.32 (each s, 6H, CH_3) ppm; ^{13}C NMR (125 MHz, $CDCl_3$): δ = 166.8 (CO), 110.1 (C), 104.3 (C-2), 78.7 (CH), 78.6 (CH), 74.9 (CH), 67.62 (CH), 67.1 (CH_2), 61.3 (CH), 53.3 (OCH_3), 36.4 (CH_2), 27.13 (CH_3), 25.3 (CH_3) ppm; HRMS (ESI): *m/z* calcd for $C_{13}H_{19}N_3O_7$ ($[M+H]^+$): 330.1301, found: 330.1293.

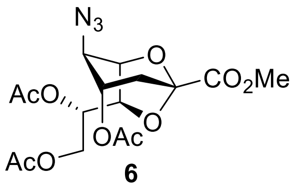

6

Methyl 4,8,9-tri-*O*-acetyl-2,7-anhydro-5-azido-3,5-dideoxy-ᴅ-glycero-α-ᴅ-galacto-2-nonulopyranosidonate (6): Compound **3** (235.8 mg, 0.815 mmol, 1 equiv) and DMAP (20 mg, 0.163 mmol, 0.2 equiv) were dissolved in dry pyridine (4 mL). To this solution, acetic anhydride (1.5 mL) was added dropwise and stirred overnight at room temperature. After the reaction was complete, the solvent was evaporated through the addition of toluene (azeotropic conditions). Then, the crude product was dissolved in 1M HCl (aq) and extracted three times with EtOAc. The extract was dried over anhydrous $MgSO_4$, filtered, and evaporated to dryness in vacuo. The crude product was purified by column chromatography using silica gel with 80% EtOAc in hexane to give **6** (261.6 mg, 77%) as colorless syrup. $[\alpha]^{25}_{D}$ +78 (c = 0.24, $CHCl_3$); IR ($CHCl_3$) *v* 2105, 1744, 1439, 1371, 1224, 1162, 1099, 1050, 1017, 798, 743 cm^{-1}; ^{1}H NMR (500 MHz, $CDCl_3$): δ = 5.05 (d, *J* = 5.5 Hz, 1H, H-4), 4.95 (m, 1H, H-8), 4.55 (s, 1H, H-6), 4.53 (dd, *J* = 2.7, 12.5 Hz, 1H, H-9a), 4.46 (d, *J* = 7.8 Hz, 1H, H-7), 4.16 (dd, *J* = 4.6, 12.4 Hz, 1H, H-9b), 3.80 (s, 3H, OCH_3), 3.44 (s, 1H, H-5), 2.36 (dd, *J* = 5.8, 15.6 Hz, 1H, H-3eq), 2.16 (d, *J* = 15.7 Hz, 1H, H-3ax), 2.08, 2.07, 2.04 ppm (each s, 9H, 3OAc); ^{13}C NMR (125 MHz, $CDCl_3$): δ = 170.6 (CO), 169.9 (CO), 169.7 (CO), 166.4 (CO), 103.7 (C-2), 77.9 (CH), 75.4 (CH), 71.1 (CH), 67.9 (CH), 62.0 (CH_2), 59.0 (CH), 53.3 (OCH_3), 33.4 (CH_2), 21.2, 21.0, 20.8 (each CH_3) ppm; HRMS (ESI): *m/z* calcd for $C_{16}H_{21}N_3O_{10}$ ($[M+Na]^+$): 438.1125, found: 438.1133.

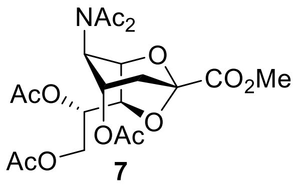

7

Methyl 4,8,9-tri-*O*-acetyl-5-(*N*-acetylacetamido)-2,7-anhydro-3,5-dideoxy-D-glycero-α-D-galacto-*2*-nonulopyranosidonate (7): Compound **5** (60 mg, 0.14 mmol, 1 equiv) and *p*-toluenesulfonic acid monohydrate (3 mg, 0.014 mmol. 0.2 equiv) were dissolved in isopropenyl acetate (0.6 mL, 40 equiv) and stirred at 80 °C for 16 h. Upon completion, the reaction was neutralized with in Et_3N (0.3 mL) and evaporated in vacuo. The crude product was purified by silica gel column chromatography (80% gradient EtOAc in hexane) to afford **7** as white foam (44.4 mg, 67%). $[\alpha]^{25}_D$ +88 (c = 0.6, $CHCl_3$); IR ($CHCl_3$) *v* 1742, 1660, 1537, 1372, 1224, 1052, 746 cm^{-1}; ^{1}H NMR (400 MHz, $CDCl_3$): δ = 5.39-5.34 (m, 1H, H-4), 4.93-4.89 (m, 1H, H-8), 4.52 (dd, *J* = 2.7, 12.4 Hz, 1H, H-9a), 4.46 (d, *J* = 1.6 Hz, 1H, H-6), 4.23 (br s, 1H, H-5), 4.22 (br d, *J* = 3.3, 1H, H-7), 4.11 (dd, *J* = 4.3, 12.5 Hz, 1H, H-9b), 3.80 (s, 3H, OCH_3), 2.97 (dd, *J* = 7.9, 14.8 Hz, 1H, H-3eq), 2.4 (s, 6H, NAc), 2.06 (s, 6H, 2OAc), 2.00 (s, 3H, 1OAc), 1.85 ppm (dd, *J* = 5.9, 14.8 Hz, 1H, H-3ax); ^{13}C NMR (100 MHz, $CDCl_3$): δ = 173.6 (CO), 170.7 (CO), 170.0 (CO), 169.9 (CO), 166.7 (CO), 103.2 (C-2), 79.1 (CH), 78.8 (CH), 70.9 (CH), 67.1 (CH), 62.0 (CH_2), 60.2 (CH), 53.4 (OCH_3), 35.8 (CH_2), 27.1, 21.1, 21.0, 20.9 (each CH_3) ppm; HRMS (ESI): *m/z* calcd for $C_{20}H_{27}NO_{12}$ ($[M+Na]^+$): 496.1431, found: 496.1432.

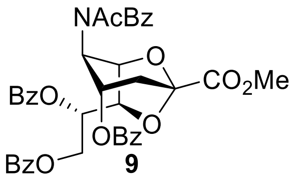

9

Methyl 2,7-anhydro-4,8,9-tri-*O*-benzoyl-5-(*N*-benzoylacetamido)-3,5-dideoxy-D-glycero-α-D-galacto-2-nonulopyranosidonate (9): Compound **2** (176 mg, 0.58 mmol, 1 equiv) and DMAP (250 mg, 2.41 mmol, 0.2 equiv) were dissolved in dry pyridine (3.5 mL). To this solution, benzoic chloride (1.17 mL) was added dropwise and kept for 15 h at room temperature. After the reaction was complete, the solvent was evaporated through the addition of toluene (azeotropic conditions). Then, the crude product was dissolved in a saturated $CuSO_4$ pentahydrate solution and extracted three times with EtOAc. The extract was dried over anhydrous $MgSO_4$, filtered, and evaporated to dryness in vacuo. The crude product was purified by silica gel column chromatography with 40% EtOAc in hexane to give **9** (201.5 mg, 57%) as colorless syrup. $[\alpha]^{25}_{D}$ +78 (c = 0.5, $CHCl_3$); ^1H NMR (400 MHz, $CDCl_3$): δ = 8.05-7.26 (m, 20H, Ar), 5.80-5.79 (m, 1H, H-4), 5.37-5.35 (m, 1H, H-8), 4.89 (br d, *J* = 2.2 Hz, 1H, H-9a), 4.87 (br s, 1H, H-6), 4.77 (d, *J* = 8.1 Hz, 1H, H-7), 4.72 (s, 1H, H-5), 4.63 (dd, *J* = 4.1, 12.4 Hz, 1H, H-9b), 3.74 (s, 3H, OCH_3), 2.95 (dd, *J* = 7.0, 15.2 Hz, 1H, H-3eq), 2.12 (dd, *J* = 2.6, 15.4 Hz, 1H, H-3ax), 1.86 ppm (s, 3H, NAc); ^{13}C NMR (100 MHz, $CDCl_3$): δ = 174.2 (CO), 172.2 (CO), 166.8 (CO), 166.2 (CO), 165.63 (CO), 165.6 (CO), 135.8 (C) , 133.9 (C), 133.5 (C) , 133.4 (C), 133.2 (CH), 130.0 (CH), 130.0 (CH), 129.8 (CH), 129.5 (CH), 129.3 (CH), 128.6 (CH), 103.9 (C-2), 79.4 (CH), 77.6 (CH), 71.8 (CH), 68.3 (CH), 62.5 (CH_2), 59.3 (CH), 53.1 (OCH_3), 34.7 (CH_2), 26.9 (CH_3) ppm; HRMS (ESI): *m/z* calcd for $C_{40}H_{35}NO_{12}$ ($[M+Na]^+$): 744.2057, found: 744.2060.

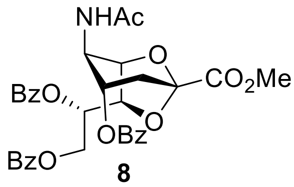

8

Methyl 5-acetamido-2,7-anhydro-4,8,9-tri-*O*-benzoyl-3,5-dideoxy-ᴅ-glycero-α-ᴅ-galacto-2-nonulopyranosidonate (8): Compound **9** (26.5 mg, 0.037 mmol, 1 equiv) was dissolved in dry Ac_2O (104 μL, 1.1 mmol, 30 equiv). $Cu(OTf)_2$ (2.6 mg, 0.008 mmol, 0.2 equiv) was added to the reaction mixture and stirred for 2 h at 70 °C. The progress of the reaction was monitored by TLC analysis. Upon completion, the mixture was azeotropically distilled with toluene at 35 °C and directly purified by flash column chromatography (40% EtOAc in hexane) to afford **8** (12 mg, 47%) as colorless syrup. $[\alpha]^{25}_{D}$ +62 (c = 1.35, $CHCl_3$); IR ($CHCl_3$) *v* 2924, 1751, 1720, 1657, 1602, 1584, 1451, 1274, 1095, 1070, 710 cm^{-1}; ^1H NMR (400 MHz, $CDCl_3$): δ = 8.03-7.91 (6H, Ar), 7.55-7.33 (7H, Ar), 7.15-7.11 (2H, Ar), 6.23 (d, *J* = 8.9 Hz, 1H, NH), 5.43-5.42 (m, 1H, H-8), 5.20 (d, *J* = 3.9 Hz, 1H, H-4), 5.06 (d, *J* = 7.7 Hz, 1H, H-7), 4.83 (br d, *J* = 12.4 Hz, 1H, H-9a), 4.64 (br s, 1H, H-6), 4.62 (d, *J* = 4.2 Hz, 1H, H-9b), 4.41 (d, *J* = 8.7 Hz, 1H, H-5), 3.77 (s, 3H, OCH_3), 2.36 (dd, *J* = 5.1, 16.0 Hz, 1H, H-3eq), 2.32 (s, 1H, H-3ax), 2.01 ppm (s, 3H, NAc); ^{13}C NMR (100 MHz, $CDCl_3$): δ = 169.3 (CO), 166.8 (CO), 166.3 (CO), 165.7 (CO), 165.3 (CO), 133.7 (C), 133.3 (C), 130.1 (C), 129.8 (CH), 129.7 (CH), 129.5 (CH), 128.7 (CH), 128.6 (CH), 104.2 (C-2), 78.8 (CH), 76.0 (CH), 71.6 (CH), 69.0 (CH), 62.6 (CH_2), 53.3 (OCH_3), 49.1 (CH), 33.7 (CH_2), 23.3 (CH_3) ppm; HRMS (ESI): *m/z* calcd for $C_{33}H_{31}NO_{11}$ ($[M+Na]^+$): 640.1795, found: 640.1802.

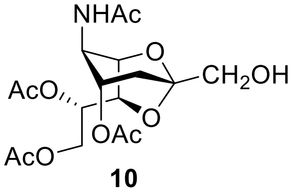

10

Hydroxymethyl 5-acetamido-4,8,9-tri-*O*-acetyl-2,7-anhydro-3,5-dideoxy-D-glycero-α-D-galacto-2-octulopyranoside (10): Compound **5** (111.9 mg, 0.276 mmol, 1 equiv) was dissolved in THF and heated to 100 °C. Then, $LiBH_4$ (10.13 µL, 0.414 mmol, 1.5 equiv) and MeOH (16.75 µL, 0.414 mmol) were added to the solution at the same temperature, and this was refluxed for 15 minutes. After the reaction was complete, the solution was neutralized by a drop of 1M HCl (aq) and concentrated in vacuo. The crude product was purified using column chromatography with 2% MeOH in $CHCl_3$ to afford **10**, a colorless syrup, in 50% yield (51.8 mg). $[\alpha]^{28}_D$ +75 (c = 0.28, $CHCl_3$); IR ($CHCl_3$) *v* 3338, 1740, 1658, 1541, 1434, 1373, 1228, 1144, 1057 cm^{-1}; ^1H NMR (400 MHz, $CDCl_3$): δ = 6.29 (d, *J* = 8.8 Hz, 1H, NH), 4.92-4.88 (m, 1H, H-8), 4.83 (d, *J* = 5.0 Hz, 1H, H-4), 4.53-4.48 (overlap, 2H, H-7, H-9a), 4.23 (s, 1H, H-6), 4.15-4.08 (overlap, 2H, H-5, H-9b), 3.62 (s, 2H, CH_2), 2.51 (broad s, OH), 2.07 (s, 6H, 2OAc), 2.05 (s, 3H, 1OAc), 2.04-1.97 (overlap, 4H, H-3eq, 1OAc), 1.86 ppm (t, *J* = 8.9 Hz, 1H, H-3ax); ^{13}C NMR (100 MHz, $CDCl_3$): δ = 170.9 (CO), 170.4 (CO), 169.7 (CO), 169.5 (CO), 107.8 (C-2), 78.1 (CH), 74.9 (CH), 71.4 (CH), 69.0 (CH), 64.9 (CH_2), 62.9 (CH_2), 49.5 (CH), 32.5 (CH_2), 23.3, 21.4, 21.1, 20.8 ppm (each CH_3). HRMS (ESI): *m/z* calcd for $C_{17}H_{25}NO_{10}$ ($[M+Na]^+$): 426.1376, found: 426.1375.

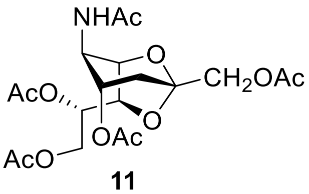

11

Methylene 5-acetamido-1,4,8,9-tetra-*O*-acetyl-2,7-anhydro-3,5-dideoxy-D-glycero-α-D-galacto-2-octulopyranoside (11): Compound **10** (59.2 mg, 0.147 mmol, 1 equiv) was dissolved in dry pyridine (1.5 mL), and DMAP (3.6 mg, 0.029 mmol, 0.2 equiv) and Ac_2O (55 µL, 40 equiv) were and stirred overnight at room temperature. After the reaction was complete, the solvent was evaporated through the addition of toluene (azeotropic conditions). The crude product was purified by silica gel column chromatography with 90% EtOAc in hexane to give **11** in 82% (53.7 mg) yield. $[\alpha]^{28}_{D}$ +76 (c = 0.97, $CHCl_3$); IR ($CHCl_3$) *v* 1741, 1660, 1537, 1372, 1223, 1054 cm^{-1}; ^1H NMR (400 MHz, $CDCl_3$): δ = 6.06 (d, *J* = 8.9 Hz, 1H, NH), 4.87-4.82 (overlap, 2H, H-8, H-4), 4.52 (d, *J* = 8.6 Hz, 1H, H-7), 4.49 (dd, *J* = 2.5, 12.4 Hz, 1H, H-9a), 4.27 (d, *J* = 12.3 Hz, 1H, H-1), 4.23 (broad s, 1H, H-6), 4.18-4.13 (overlap, 2H, H-5, H-9b), 4.06 (d, *J* = 12.3 Hz, 1H, H-1), 2.11, 2.08, 2.07, 2.06, 2.01 (each s, 15H, 5OAc), 1.99-1.89 (overlap, 2H, H-3eq, H-3ax) ppm; ^{13}C NMR (100 MHz, $CDCl_3$): δ = 170.6 (CO), 170.4 (CO), 170.2 (CO), 169.6 (CO), 169.3 (CO), 106.5 (C-2), 78.3 (CH), 74.8 (CH), 71.2 (CH), 68.7 (CH), 64.5 (CH_2), 62.3 (CH_2), 49.3 (CH), 32.8 (CH_2), 23.3, 21.4, 21.1, 20.8 (each CH_3) ppm. HRMS (ESI): *m/z* calcd for $C_{19}H_{27}NO_{11}$ ($[M+Na]^+$): 468.1476, found: 468.1474.

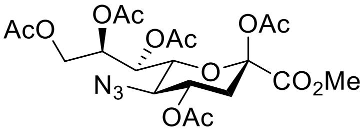

12

Methyl 2,4,7,8,9-penta-*O*-acetyl-5-azido-3,5-dideoxy-ᴅ-glycero-β-ᴅ-galacto-2-nonulopyranosidonate (12): Compound **6** (36 mg, 0.09 mmol, 1 equiv) was dissolved in dry Ac_2O (250 μL, 2.66 mmol, 30 equiv). $SnCl_4$ (5.2 μL, 0.044 mmol, 0.5 equiv) was added to the reaction mixture, and this was stirred for 19 h at room temperature. The progress of the reaction was monitored by TLC analysis. Upon completion, the solvent was removed with toluene (azeotropic distillation) at 35 °C and directly purified by flash column chromatography (40% EtOAc in hexane) to afford **12** (24.84 mg, 54%) and **13** (15.87 mg, 34%) as colorless syrups. $[\alpha]^{25}_{D}$ -56 (c = 0.87, $CHCl_3$); IR ($CHCl_3$) *v* 2116, 1746, 1438, 1372, 1222, 1199, 1166, 1085 cm^{-1}; ^1H NMR (500 MHz, $CDCl_3$): δ = 5.49 (dd, *J* = 1.6, 6.9 Hz, 1H, H-7), 5.17-5.15 (overlap, 2H, H-4, H-8), 4.39 (dd, *J* = 2.5, 12.6 Hz, 1H, H-9a), 4.16 (dd, *J* = 5.3, 12.6 Hz, 1H, H-9b), 3.74 (dd, *J* = 1.7, 10.7 Hz, 1H, H-6), 3.74 (s, 3H, OCH_3), 3.33 (t, *J* = 10.0 Hz, 1H, H-5), 2.63 (dd, *J* = 5.1, 13.5 Hz, 1H, H-3eq), 2.17, 2.10, 2.08, 2.03, 2.02 (each s, 15H, 5OAc), 1.88 ppm (dd, *J* = 11.61, 13.5 Hz, 1H, H-3ax); ^{13}C NMR (125 MHz, $CDCl_3$): δ =170.8 (CO), 170.0 (CO), 169.8 (CO), 169.7 (CO), 168.3 (CO), 166.3 (CO), 97.2 (C-2), 71.7 (CH), 70.4 (CH), 70.0 (CH), 68.4 (CH), 61.9 (CH_2), 59.9 (CH), 53.3 (OCH_3), 35.5 (CH_2), 21.0, 20.9, 20.9, 20.8, 20.8 (each CH_3) ppm; HRMS (ESI): *m/z* calcd for $C_{20}H_{27}N_3O_{13}$ ($[M+Na]^+$): 540.1442, found: 540.1434.

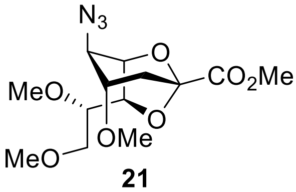

21

Methyl 2,7-anhydro-5-azido-4,8,9-tri-*O*-methyl-3,5-dideoxy-ᴅ-glycero-α-ᴅ-galacto-2-nonulopyranosidonate (21): Compound **3** (38.1 mg, 0.132 mmol, 1 equiv) was dissolved in dry DMF (2 mL) and cooled to 0 °C. Then, NaH (60%, 26.3 mg, 0.659 mmol, 5 equiv) was added slowly, and this was stirred for 30 min until the evolution of hydrogen gas ceased. Methyl iodide (49.2 µL, 0.79 mmol, 6 equiv) was added dropwise over 15 minutes. The reaction mixture was stirred overnight at room temperature. Upon completion of the reaction, the mixture was quenched with ice-cold water and diluted with EtOAc (5 mL). The desired product was extracted with EtOAc (three times) from the aqueous phase and separated. Then, the combined organic layers were dried over anhydrous $MgSO_4$, filtered, and concentrated. Silica gel column chromatography (hexane:EtOAc, 1:1, v/v) afforded compound **21** as colorless syrup (17 mg, 40%). $[\alpha]^{25}_{D}$ +57 (c = 1.85, $CHCl_3$); IR ($CHCl_3$) *v* 2933, 2101, 1752, 1441, 1309, 1262, 1193, 1091, 1048, 743 cm^{-1}; ^1H NMR (500 MHz, $CDCl_3$): δ = 4.69 (s, 1H, H-6), 4.36 (d, *J* = 9.1 Hz, 1H, H-7), 3.79 (s, 3H, OCH_3), 3.66 (dd, *J* = 2.4, 10.7 Hz, 1H, H-9a), 3.52 (br dd, *J* = 1.3, 5.9 Hz, 1H, H-4), 3.48 (dd, *J* = 3.8, 10.7 Hz, 1H, H-9b), 3.44 (br s, 1H, H-5), 3.42, 3.36, 3.34 (each s, 9H, OMe), 3.18-3.15 (m, 1H, H-8), 2.24 (d, *J* = 15.1 Hz, 1H, H-3eq), 2.15 (dd, *J* = 5.7, 15.2 Hz, 1H, H-3ax); ^{13}C NMR (125 MHz, $CDCl_3$): δ = 167.3 (CO), 103.6 (C-2), 79.8 (CH), 78.3 (CH), 76.1 (CH), 75.8 (CH), 69.9 (CH_2), 59.5 (CH), 58.5 (OCH_3), 58.0 (OCH_3)), 57.7 (OCH_3), 57.1 (OCH_3), 33.6

(CH_2) ppm; HRMS (ESI): *m/z* calcd for $C_{13}H_{21}N_3O_7$ ($[M+Na]^+$): 354.1277, found: 354.1268.

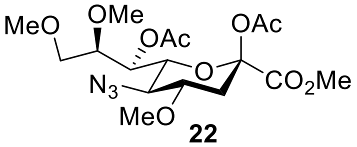

22

Methyl 2,7-di-*O*-acetyl-5-azido-4,8,9-tri-*O*-methyl-3,5-dideoxy-D-glycero-β-D-galacto-2-nonulopyranosidonate (22): Compound **21** (16.3 mg, 0.0.05 mmol, 1 equiv) was dissolved in dry Ac_2O (150 μL, 1.48 mmol, 30 equiv). $SnCl_4$ (2.9 μL, 0.025 mmol, 0.6 equiv) was added to the reaction mixture, and this was stirred for 2 h at room temperature. The progress of the reaction was monitored by TLC analysis. Upon completion, the mixture was azeotroped with toluene at 35 °C and directly purified by flash column chromatography (50% EtOAc in hexane) to give **22** (14.14 mg, 66%) and glycal **23** (6 mg, 27%) as colorless syrups. $[\alpha]^{25}_{D}$ -133 (c = 0.29, $CHCl_3$); IR ($CHCl_3$) *v* 2929, 2115, 1750, 1556, 1441, 1271, 1232, 1205, 1163, 1101, 929 cm^{-1}; ^1H NMR (500 MHz, $CDCl_3$): δ = 5.34 (dd, *J* = 1.5, 9.2 Hz, 1H, H-7), 3.81 (dd, *J* = 1.5, 10.9 Hz, 1H, H-6), 3.77 (s, 3H, OCH_3), 3.65-3.60 (m, 1H, H-4), 3.57 (dd, *J* = 2.9, 10.7 Hz, 1H, H-9a), 3.54-3.50 (m, 1H, H-8), 3.45, 3.32, 3.30 (each s, 9H, OMe), 3.27 (dd, *J* = 3.8, 10.8 Hz, 1H, H-9b), 3.13 (dd, *J* = 1.4, 10.8 Hz, 1H, H-5), 2.59 (dd, *J* = 5.0, 13.5 Hz, 1H, H-3eq), 2.11, 2.08 (each s, 6H, 2OAc), 1.66 (dd, *J* = 2.2, 13.4 Hz, 1H, H-3ax); ^{13}C NMR (125 MHz, $CDCl_3$): δ = 170.0 (CO), 168.4 (CO), 167.4 (CO), 97.4 (C-2), 77.9 (CH), 76.6 (CH), 71.1 (CH), 70.3 (CH_2), 69.1 (CH), 61.4 (CH), 59.5 (OCH_3), 57.6 (OCH_3), 57.4 (OCH_3), 53.2 (OCH_3), 35.7 (CH_2), 21.0, 20.8 (each CH_3) ppm; HRMS (ESI): *m/z* calcd for $C_{17}H_{27}N_3O_{10}$ ($[M+Na]^+$): 456.1594, found: 456.1594.

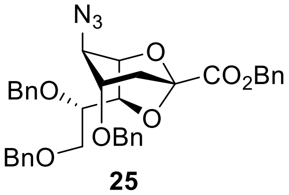

25

Benzyl 2,7-anhydro-5-azido-4,8,9-tri-*O*-benzyl-3,5-dideoxy-ᴅ-glycero-α-ᴅ-galacto-2-nonulopyranosidonate (25): Compound **3** (50 mg, 0.173 mmol, 1 equiv) was dissolved in dry DMF (2 mL), and cooled to 0 °C. Then, NaH (60%) (41 mg, 1.04 mmol, 6 equiv) was added slowly, and this was stirred for 30 min until hydrogen gas evolution ceased. Benzyl bromide (123.4 µL, 1.04 mmol, 6 equiv) was added dropwise over 15 minutes. The reaction mixture was stirred overnight at room temperature. Upon completion of the reaction, the mixture was quenched with ice water and diluted with CH_2Cl_2 (10 mL). The desired product was extracted with CH_2Cl_2 (three times) from the aqueous phase and separated. Then, the combined organic layer was dried over anhydrous $MgSO_4$, filtered, and concentrated. Silica gel column chromatography (9:1, hexane:EtOAc, v/v) afforded compound **25** as colorless syrup (45.3 mg, 50%). $[\alpha]^{25}_D$ +28 (c = 0.5, $CHCl_3$); IR ($CHCl_3$) *v* 2919, 2099, 1749, 1586, 1496, 1454, 1260, 1204, 1156, 1089, 737, 697 cm^{-1}; ^1H NMR (600 MHz, $CDCl_3$): δ = 7.32-7.25 (m, 20H, Ar), 5.24 (d, *J* = 12.3 Hz, 1H, OCH_2), 5.18 (d, *J* = 12.3 Hz, 1H, OCH_2), 4.71-4.69 (overlap, 2H, H-6, OCH_2), 4.57-4.46 (overlap, 6H, H-7, OCH_2), 3.37-3.71 (overlap, 2H, H-4, H-9a), 3.58 (dd, *J* = 4.7, 10.8 Hz, 1H, H-9b), 3.54-3.52 (m, 1H, H-8), 3.46 (s, 1H, H-5), 2.28 (d, *J* = 15.2 Hz, 1H, H-3eq), 2.18 ppm (dd, *J* = 5.8, 15.1 Hz, 1H, H-3ax); ^{13}C NMR (150 MHz, $CDCl_3$): δ = 166.7 (CO), 138.5, 138.2, 137.4, 135.2 (each C), 128.7 (CH), 128.7 (CH), 128.6 (CH), 128.5 (CH), 128.3 (CH), 128.2 (CH), 128.0 (CH), 128.0 (CH), 127.8 (CH), 127.7 (CH), 127.7 (CH), 103.7 (C-2), 78.2 (CH), 78.2 (CH), 76.5 (CH), 73.5 (OCH_2), 73.3 (CH), 72.7 (OCH_2), 71.5 (OCH_2), 69.1 (CH_2), 67.6 (OCH_2), 59.4 (CH),

33.6 ppm (CH_2); HRMS (ESI): *m/z* calcd for $C_{37}H_{37}N_3O7$ ([M+Na]$^+$): 658.2529, found: 658.2523.

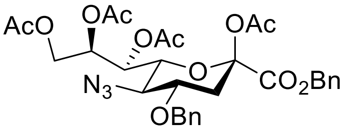

26

Benzyl 2,7,8,9-tetra-*O*-acetyl-5-azido-4-*O*-benzyl-3,5-dideoxy-ᴅ-glycero-β-ᴅ-galacto-2-nonulopyranosidonate (26): Compound **25** (20 mg, 0.03 mmol, 1 equiv) was dissolved in dry Ac_2O (100 µL, 0.94 mmol, 35 equiv). $SnCl_4$ (1 µL, 0.008 mmol, 0.25 equiv) was added to the reaction mixture, and this was stirred for 0.5 h at room temperature. The progress of the reaction was monitored by TLC analysis. Upon completion, the solvent was removed using toluene (azeotropic distillation) at 35 °C and directly purified by flash column chromatography (25% EtOAc in hexane) to give **26** (6.1 mg, 27%) and **27** (11.96 mg, 52%) as colorless syrups. Compound **26**: $[\alpha]^{33}_D$ -19 (c = 0.175, $CHCl_3$); IR ($CHCl_3$) *v* 2924, 2115, 1751, 1371, 1218, 1056, 773, 698 cm^{-1}; ^1H NMR (400 MHz, $CDCl_3$): δ = 7.36-7.29 (m, 10H, Ar), 5.50 (d, *J* = 6.4 Hz, 1H, H-7), 5.21-5.13 (m, 3H, H-8, OCH_2), 4.64 (d, *J* = 11.1 Hz, 1H, OCH_2), 4.58 (d, *J* = 11.1 Hz, 1H, OCH_2), 4.39 (d, *J* = 12.7 Hz, 1H, H-9a), 4.16 (dd, *J* = 5.1, 12.5 Hz, 1H, H-9b), 3.83-3.77 (m, 1H, H-4), 3.66 (d, *J* = 10.7 Hz, 1H, H-6), 3.26 (dd, *J* = 6.1, 16.2 Hz, 1H, H-5), 2.66 (dd, *J* = 4.9, 13.6 Hz, 1H, H-3eq), 2.11, 2.03, 2.01, 1.99 (each s, 12H, 4OAc), 1.82 (t, *J* = 12.6 Hz, 1H, H-3ax); ^{13}C NMR (100 MHz, $CDCl_3$): δ = 170.8 (CO), 170.0 (CO), 169.7 (CO), 168.3 (CO), 166.0 (CO), 137.1 (C), 135.1 (C), 128.7 (CH), 128.7 (CH), 128.6 (CH), 128.3 (CH), 128.3 (CH), 128.1 (CH), 97.9 (C-2), 75.5 (CH), 72.0 (CH), 72.0 (OCH_2), 70.4 (CH), 68.6 (CH), 68.0 (OCH_2), 62.0 (CH), 61.2 (CH_2), 35.4 (CH_2), 20.9 (CH_3), 20.8 ppm (CH_3); HRMS (ESI): *m/z* calcd for $C_{31}H_{35}N_3O_{12}$ ([M+Na]$^+$): 664.2118, found: 664.2121.

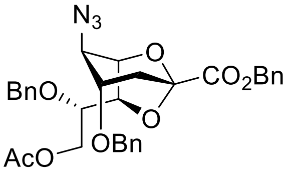

27

Benzyl 9-*O*-acetyl-2,7-anhydro-5-azido-4,8-di-*O*-benzyl-3,5-dideoxy-D-glycero-α-D-galacto-2-nonulopyranosidonate (27): $[\alpha]^{28}_{D}$ +20 (c = 0.26, $CHCl_3$); IR ($CHCl_3$) *v* 2953, 2920, 2852, 2101, 1742, 1455, 1378, 1305, 1247, 1099, 1089, 1050, 741, 699 cm^{-1}; ^{1}H NMR (600 MHz, $CDCl_3$): δ = 7.34-7.26 (m, 15H, Ar), 5.24 (d, *J* = 12.2 Hz, 1H, OCH_2), 5.18 (d, *J* = 12.2 Hz, 1H, OCH_2), 4.67 (d, *J* = 11.1 Hz, 1H, OCH_2), 4.62-4.59 (overlap, 2H, H-7, H-9a), 4.56 (d, *J* = 11.9 Hz, 1H, OCH_2), 4.51-4.48 (overlap, 2H, H-6, OCH_2), 4.43 (d, *J* = 11.2 Hz, 1H, OCH_2), 4.02 (dd, *J* = 1.4, 5.6 Hz, 1H, H-9b), 3.74 (dd, *J* = 3.5, 12.2 Hz, 1H, H-4), 3.50-3.47 (m, 2H, H-8, H-5), 2.31 (d, *J* = 15.1 Hz, 1H, H-3eq), 2.18 (dd, *J* = 5.6, 15.2 Hz, 1H, H-3ax), 1.99 (s, 3H, 1OAc) ppm; ^{13}C NMR (150 MHz, $CDCl_3$): δ = 170.9 (CO), 166.52 (CO), 137.5 (C), 137.4 (C), 135.1 (C), 128.8 (CH), 128.8 (CH), 128.7 (CH), 128.4 (CH), 128.4 (CH), 128.1 (CH), 127.7 (CH), 103.81 (C-2), 78.4 (CH), 75.9 (CH), 72.4 (OCH_2), 71.6 (OCH_2), 67.8 (OCH_2), 61.2 (CH_2), 59.3 (CH), 33.3 (CH_2), 21.0 (CH_3) ppm; HRMS (ESI): *m/z* calcd for $C_{32}H_{33}N_3O_8$ ($[M+Na]^+$): 610.2165, found: 610.2173.

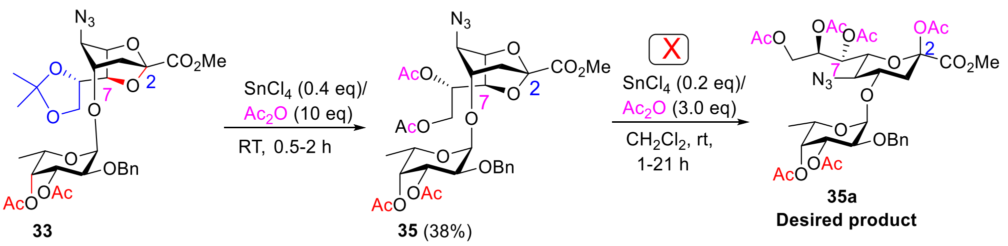

Methyl 8,9-di-*O*-acetyl-2,7-anhydro-5-azido-3,5-dideoxy-4-*O*-(3,4-di-*O*-acetyl-2-*O*-benzyl-α-ʟ-fucopyranosyl)-ᴅ-glycero-α-ᴅ-galacto-2-nonulopyranosidonate (35): Disaccharide **33** (29.2 mg, 0.045 mmol, 1 equiv) was dissolved in dry Ac_2O (42.5 µL, 0.45 mmol, 10 equiv). $SnCl_4$ (2.1 µL, 0.018 mmol, 0.4 equiv) was added to the solution, and this was kept for 0.5 to 2 h at rt. Upon completion, the reaction was azeotroped with toluene at 35 °C. Purification of the crude product with flash column chromatography using 50% EtOAc in hexane gave **35** (12.8 mg, 38%) as colorless syrup. Furthermore, disaccharide **35** was subjected to an acetolysis reaction with a reduced amount of $SnCl_4$ and Ac_2O in CH_2Cl_2, but the reaction did not proceed even with an increased reaction time. $[\alpha]^{28}_{D}$ -5.61 (c = 0.41, $CHCl_3$); IR ($CHCl_3$) *v* 2922, 2104, 1746, 1371, 1243, 1223, 1096, 1079, 1048 cm^{-1}; ^1H NMR (500 MHz, $CDCl_3$): δ = 7.37-7.29 (m, 5H, Ar), 5.24 (br d, *J* = 3.3 Hz, 1H, H-4), 5.19 (dd, *J* = 3.3, 10.5 Hz, 1H, H-3), 4.96-4.93 (m, 1H, H'-8), 4.76-4.71 (overlap, 3H, H'-7, H-1, OCH_2), 4.52-4.47 (overlap, 3H, H'-6, H'9a, OCH_2), 4.22 (q, *J* = 6.4, 13.2 Hz, 1H, H-5), 4.1 (dd, *J* = 4.6, 12.5 Hz, 1H, H'-9b), 3.83-3.80 (overlap, 5H, OCH_3, H-2, H'-4), 3.21 (br s, 1H, H'-5), 2.28 (d, *J* = 15.0 Hz, 1H, H'-3eq), 2.21 (dd, *J* = 5.5, 15.4 Hz, 1H, H'-3ax), 2.14, 2.06, 2.00, 1.95 (each s, 12H, 4OAc), 1.07 (d, *J* = 6.6 Hz, 3H, 5-CH_3) ppm; ^{13}C NMR (125 MHz, $CDCl_3$): δ = 170.9 (CO), 170.6 (CO), 170.1 (CO), 169.9 (CO), 166.7 (CO), 138.2 (C), 128.9 (CH), 128.5 (CH), 128.1 (CH), 103.7 (C'-2), 99.0 (CH-1), 77.9 (CH), 75.2 (CH), 74.5 (CH), 74.4 (OCH_2), 71.6 (CH), 71.4 (CH), 70.2 (CH), 65.6 (CH), 61.9 (CH_2), 58.9 (CH), 53.2

(OCH_3), 34.4 (CH_2), 21.1, 20.9, 20.8, 20.7,15.9 (each CH_3); HRMS (ESI): *m/z* calcd for $C_{31}H_{39}N_3O_{15}$ ($[M+Na]^+$): 716.2279, found: 716.2277.

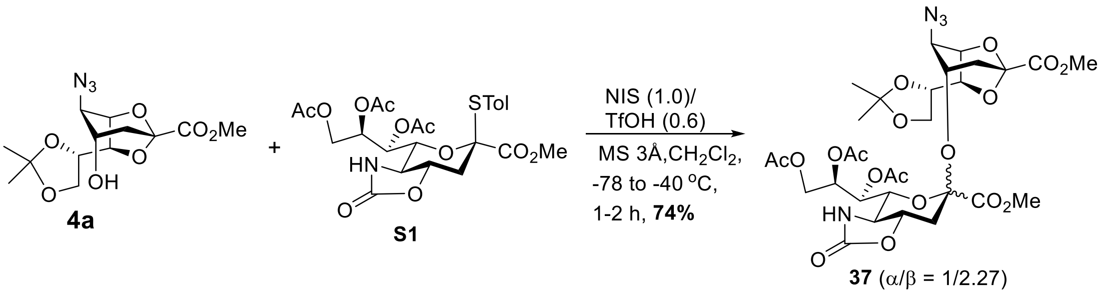

Methyl 2,7-anhydro-5-azido-3,5-dideoxy-8,9-*O*-isopropylidene-4-*O*-(methyl 7,8,9-tri-*O*-acetyl-5-*N*,4-*O*-carbonyl-3,5-dideoxy-ᴅ-glycero-α-ᴅ-galacto-2-nonulopyranosidonate)-ᴅ-glycero-α/β-ᴅ-galacto-2-nonulopyranosidonate (37): Donor **S1** [1] (46.6 mg, 0.086 mmol, 1.2 equiv) and acceptor **4a** (23.7 mg, 0.072 mmol, 1 equiv) were azeotroped with toluene at 35 °C and dissolved in dry CH_2Cl_2 (1.5 mL). Freshly activated 3 Å molecular sieve (120 mg) was added, and the mixture was stirred at rt for 1 h under N_2 atmosphere. The reaction mixture was cooled to −40 °C and NIS (26.0 mg, 0.115 mmol, 1 equiv) and TfOH (3.8 µL, 0.043 mmol, 0.6 equiv) were added, and this was stirred for 1 h. The completion of the reaction was confirmed by TLC analysis. The reaction was then allowed to warm to 0 °C for 0.5 h. The reaction was quenched with Et_3N, diluted with CH_2Cl_2, filtered through Celite, and further neutralized with a saturated aqueous solution of $NaHCO_3$. The organic layer was washed with a 10% aq $Na_2S_2O_3$ solution, treated with brine, dried over anhydrous $MgSO_4$, filtered, and concentrated in vacuo. Purification of the crude product by flash column chromatography on silica gel (EtOAc:hexanes, 1:4, v/v) afforded disaccharide **37** (α/β = 1.0/2.27, 39.2 mg, 74%) as colorless syrup. Compound **37β**: $[\alpha]^{19}_D$ +15.75 (c = 0.64, $CHCl_3$); IR ($CHCl_3$) *v* 2924, 2854, 2105, 1746, 1454, 1374, 1223, 1157, 1071, 760 $cm^{-1;}$ ^1H NMR (500

MHz, $CDCl_3$): δ = 5.37 (s, 1H, NH'), 5.30 (m, 1H, H'-8), 5.12 (dd, *J* = 2.0, 6.5 Hz, 1H, H'-7), 4.67 (br s, 1H, H-6), 4.60 (dd, *J* = 1.8, 12.7 Hz, 1H, H'-9a), 4.47 (td, *J* = 3.7,12.2 Hz, 1H, H'-4), 4.19-4.14 (overlap, 3H, H-7, H-4, H'-9b), 4.08 (dd, *J* = 5.9, 8.6 Hz, 1H, H-9a), 3.99 (dd, *J* = 2.0, 9.8 Hz, 1H, H'-6), 3.97-3.89 (overlap, 2H, H-8, H-9b), 3.84 (s, 3H, CO_2Me'), 3.80 (s, 3H, CO_2Me), 3.47 (br s, 1H, H-5), 3.07 (t, *J* = 10.7 Hz, 1H, H'-5), 2.65 (dd, *J* = 3.8, 12.3 Hz, 1H, H'-3eq), 2.25 (dd, *J* = 5.8, 15.1 Hz, 1H, H-3eq), 2.16 (s, 3H, 1OAc), 2.15 (t, *J* = 7.3 Hz, 1H, H'-3ax), 2.10 (s, 3H, 1OAc), 2.01 (s, 3H, 1OAc), 1.88 (d, *J* = 15.0 Hz, 1H, H-3ax), 1.40 (s, 3H, CH_3), 1.31 ppm (s, 3H, CH_3); ^{13}C NMR (125 MHz, $CDCl_3$): δ = 171.2 (CO), 170.7 (CO), 170.3 (CO), 167.3 (CO), 166.9 (CO), 159.2 (CO), 110.0 (C), 103.1 (C-2), 100.2 (C'-2), 78.5 (CH), 78.4 (CH), 76.0 (CH), 75.1 (CH), 74.0 (CH), 70.1 (CH), 69.5 (CH), 69.4 (CH), 67.2 (CH_2), 61.9 (CH_2), 59.8 (CH), 58.1 (CH), 53.6 (OMe), 53.3 (OMe), 37.9 (CH_2), 35.7 (CH_2), 27.1, 25.3, 21.2, 20.8, 20.8 (each CH_3) ppm; HRMS (ESI): *m/z* calcd for $C_{30}H_{40}N_4O_{18}$ ($[M+Na]^+$): 767.2235, found: 767.2242. Compound **37α:** $[\alpha]^{20}_D$ +17.60 (c = 0.40, $CHCl_3$); IR ($CHCl_3$) *v* 2970, 2928, 2106, 1739,1723, 1436, 1366, 1228, 1217, 1096, 1064 cm^{-1}; ^1H NMR (500 MHz, $CDCl_3$): δ = 5.49 (dt, *J* = 2.1, 10.05 Hz, 1H, H'-8), 5.36 (s, 1H, NH'), 5.10 (dd, *J* = 1.6, 10.1 Hz, 1H, H'-7), 4.74 (br s, 1H, H-6), 4.34-4.20 (overlap, 4H, H'-6, H'-9a, H'-9b, H-7), 4.10-4.02 (overlap, 3H, H'-4, H-9a, H-4), 4.00-3.96 (m, 1H, H-8), 3.92 (dd, *J* = 4.9, 8.57 Hz, 1H, H-9b), 3.80 (s, 3H, CO_2Me'), 3.78 (s, 3H, CO_2Me), 3.54 (br s, 1H, H-5), 3.01 (t, *J* = 10.9 Hz, 1H, H'-5), 2.91 (dd, *J* = 3.6, 12.3 Hz, 1H, H'-3eq), 2.20-2.13 (overlap, 2H, H-3eq, H-3ax), 2.15 (s, 3H, 1OAc), 2.15 (s, 3H, 1OAc), 2.04 (s, 3H, 1OAc), 2.00 ppm (t, *J* = 12.8 Hz, 1H, H'-3ax), 1.40 (s, 3H, CH_3), 1.32 ppm (s, 3H, CH_3); ^{13}C NMR (125 MHz, $CDCl_3$): δ = 171.4 (CO), 170.7 (CO), 169.7 (CO), 168.6 (CO), 166.9 (CH), 159.2 (CO), 110.0 (C), 103.1 (C-2), 100.2 (C'-2), 78.5 (CH), 78.4 (CH), 76.0 (CH), 75.1 (CH), 74.0 (CH), 70.1 (CH), 69.5 (CH), 69.4 (CH),

67.2 (CH_2), 61.9 (CH_2), 59.8 (CH), 58.1 (CH), 53.6 (OMe), 53.3 (OMe), 37.9 (CH_2), 35.7 (CH_2), 27.1, 25.30, 21.2, 20.8, 20.8 ppm (each CH_3); HRMS (ESI): *m/z* calcd for $C_{30}H_{40}N_4O_{18}$ ($[M+Na]^+$): 767.2230, found: 767.2222.

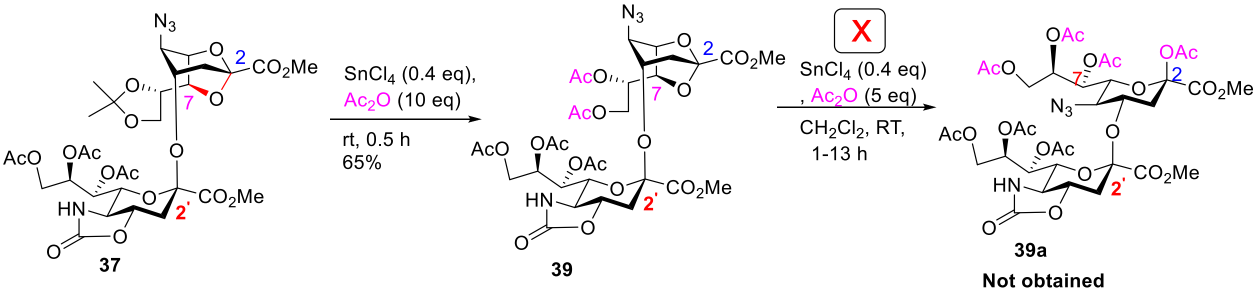

Methyl 8,9-di-*O*-acetyl-2,7-anhydro-5-azido-3,5-dideoxy-4-*O*-(methyl 7,8,9-tri-*O*-acetyl-5-*N*,4-*O*-carbonyl-3,5-dideoxy-ᴅ-glycero-α-ᴅ-galacto-2-nonulopyranosidonate)-ᴅ-glycero-β-ᴅ-galacto-2-nonulopyranosidonate (39): Compound **37** (18.6 mg, 0.025 mmol, 1 equiv) was dissolved in dry Ac_2O (23.6 µL, 0.25 mmol, 10 equiv). $SnCl_4$ (1.2 µL, 0.001 mmol, 0.4 equiv) was added dropwise to the solution, and this was kept for 0.5 h at rt. Upon completion, the reaction mixture was azeotropically distilled at 35 °C after the addition of toluene. Purification of the crude product by flash column chromatography using 50% EtOAc in hexane gave **39** (12.8 mg, 65%) as colorless syrup. Furthermore, disaccharide **39** was subjected to an acetolysis reaction with a reduced amount of Ac_2O in CH_2Cl_2, but the reaction did not proceed even with increased reaction time. $[\alpha]^{20}_D$ +0.89 (c = 0.26, $CHCl_3$); IR ($CHCl_3$) *v* 2954, 2919, 2850, 2107, 1738, 1463, 1376, 1228, 1217, 1091, 1019, 761 cm^{-1}; ^1H NMR (500 MHz, $CDCl_3$): δ = 5.38 (br s, 1H, NH'), 5.30 (td, *J* = 1.9, 6.5 Hz, 1H, H'-8), 5.13 (dd, *J* = 2.2, 6.5 Hz, 1H, H'-7), 4.94-4.92 (m, 1H, H-8), 4.58 (dd, *J* = 1.9, 12.7 Hz, 1H, H'-9a), 4.54-4.45 (overlap, 4H, H'-4, H-7, H-6, H-9a), 4.20-4.13 (overlap, 3H, H-4, H-9b, H'-9b), 4.04 (dd, *J* = 2.2, 9.8 Hz, 1H, H'-6), 3.86 (s, 3H, CO_2Me'), 3.81 (s, 3H, CO_2Me), 3.53 (br

s, 1H, H-5), 3.09 (t, *J* = 10.9 Hz, 1H, H'-5), 2.61 (dd, *J* = 3.8, 12.17 Hz, 1H, H'-3eq), 2.28 (dd, *J* = 5.7, 15.2 Hz, 1H, H-3eq), 2.18 (t, *J* = 13.9 Hz, 1H, H'-3ax), 2.16 (s, 3H, 1Ac), 2.12 (s, 3H, 1Ac), 2.09 (s, 3H, 1Ac), 2.05 (s, 3H, 1Ac), 2.02 (s, 3H, 1Ac), 1.91 ppm (d, *J* = 15.1 Hz, 1H, H-3ax); ^{13}C NMR (125 MHz, $CDCl_3$): δ = 171.2 (CO), 170.7 (CO), 170.7 (CO), 170.4 (CO), 170.0 (CO), 167.4 (CO), 166.6 (CO), 159.0 (CO),103.3 (C-2), 100.5 (C'-2), 78.1 (CH), 75.9 (CH), 75.2 (CH), 74.0 (CH), 71.1 (CH), 70.1 (CH), 69.8 (CH), 69.4 (CH), 61.9 (CH_2), 59.8 (CH), 58.3 (CH), 53.7 (OMe), 53.4 (OMe), 38.1 (CH_2), 35.2 (CH_2), 21.2, 21.1, 20.8, 20.8 ppm (each CH_3); HRMS (ESI): *m/z* calcd for $C_{31}H_{40}N_4O_{20}$ ($[M+Na]^+$): 811.2128, found: 811.2133.

NMR spectra

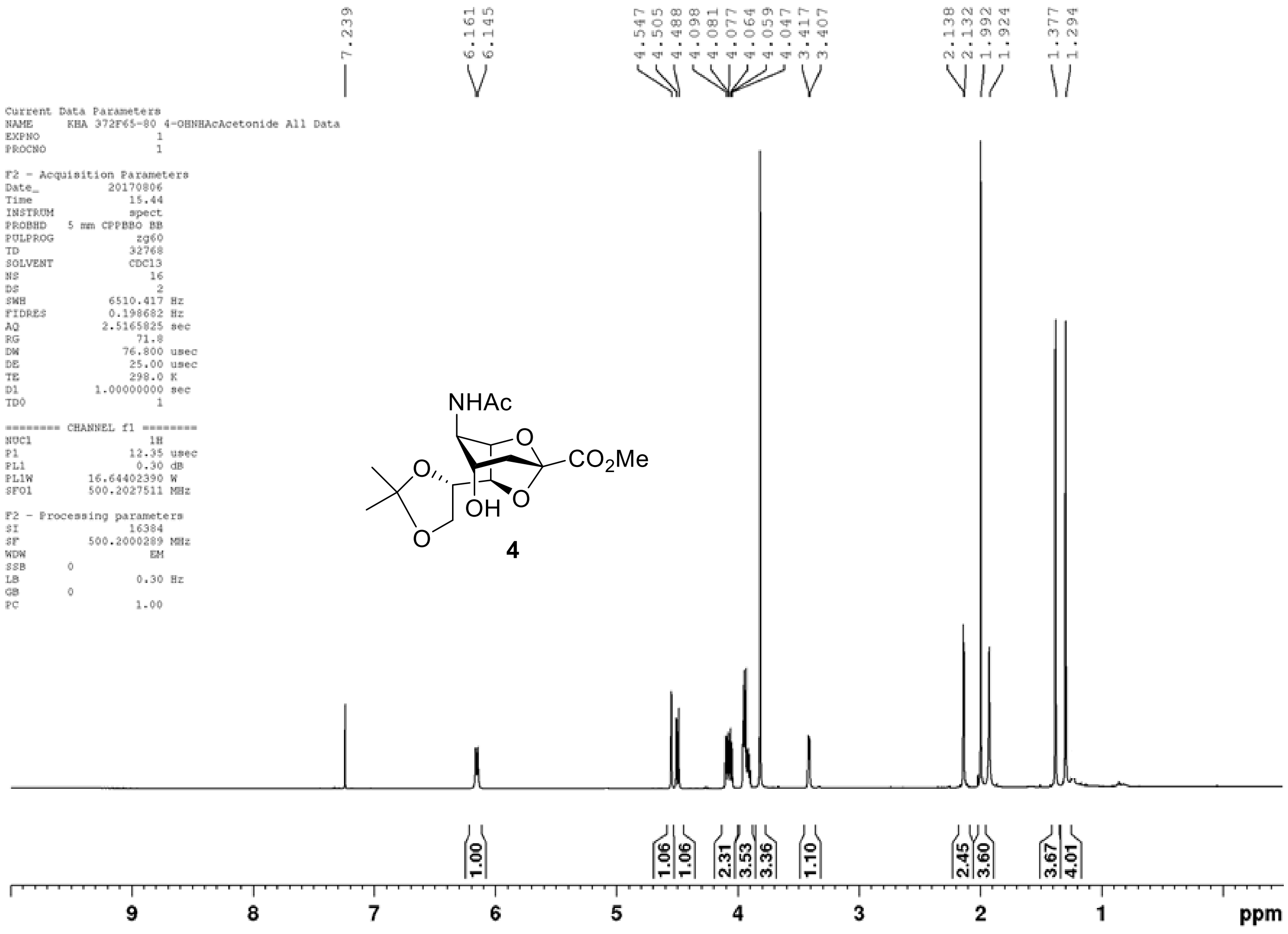

The ^{1}H NMR spectrum of compound **4**.

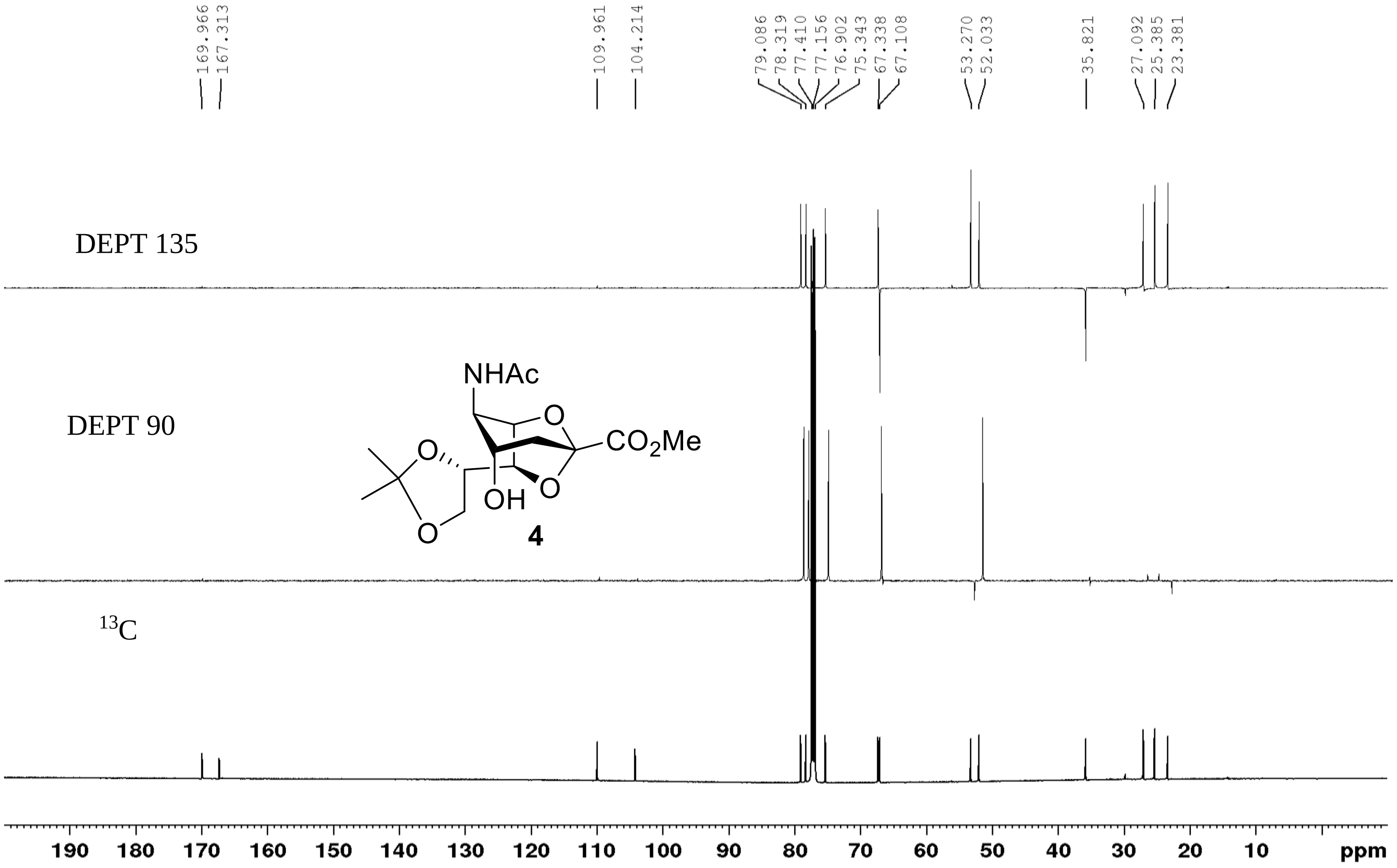

The ^{13}C NMR spectrum of compound **4**.

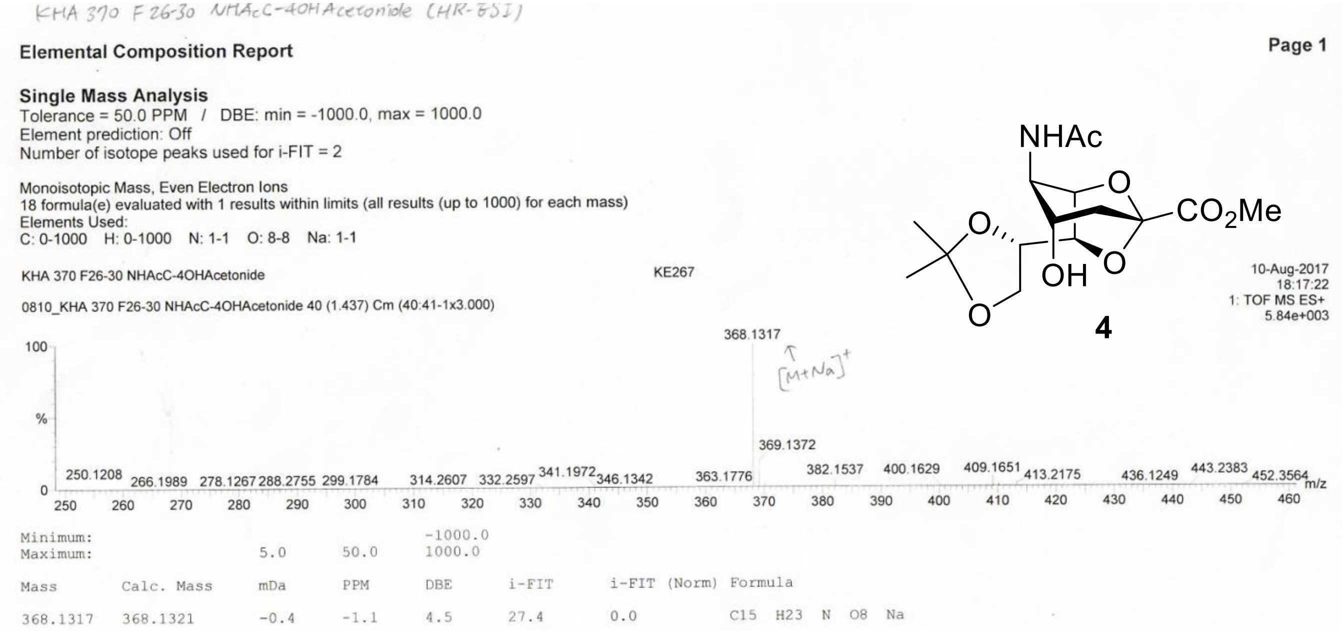

The HRMS spectrum of compound **4**.

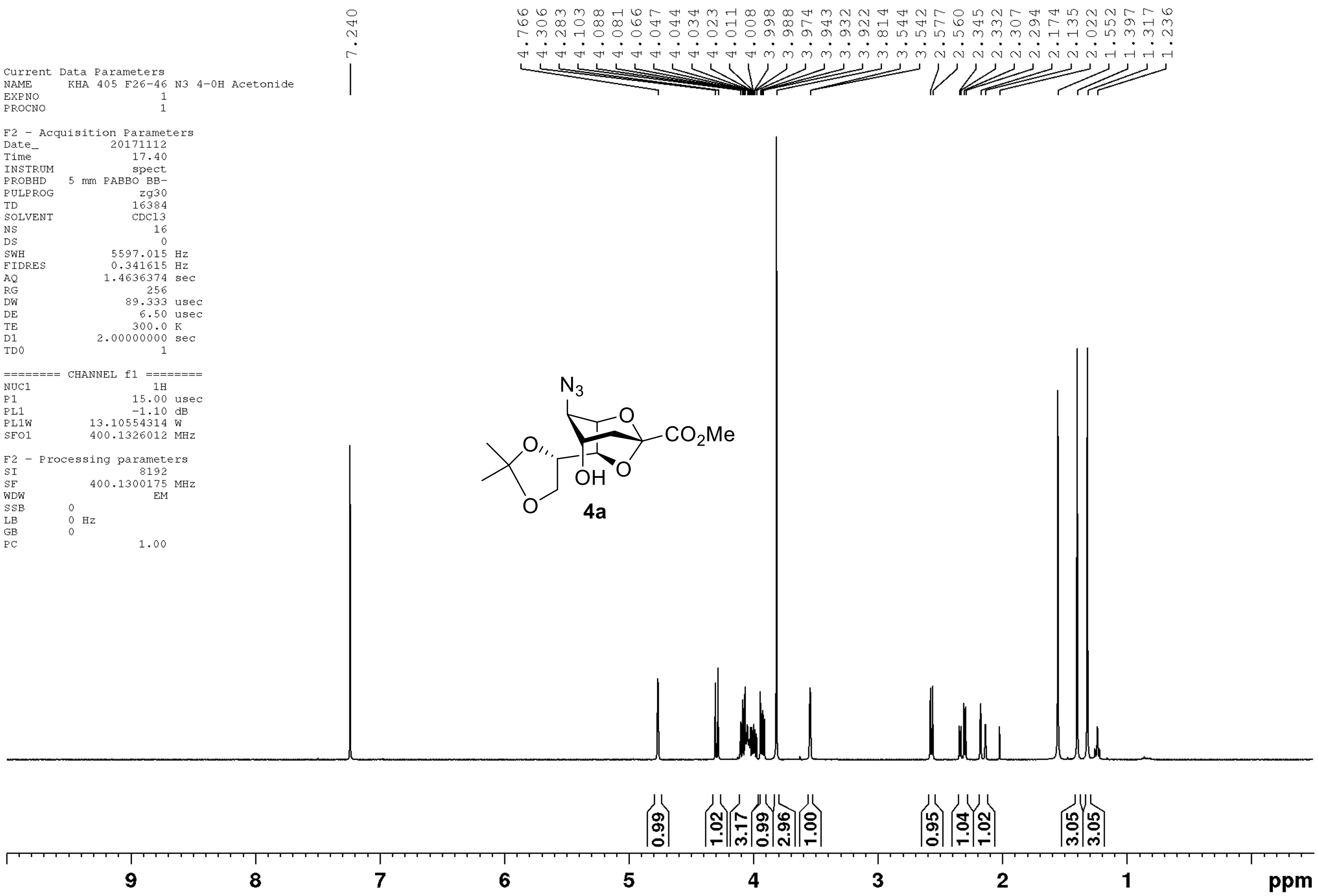

^{1}H NMR spectrum of compound **4a** (500 MHz, $CDCl_3$, 25 °C).

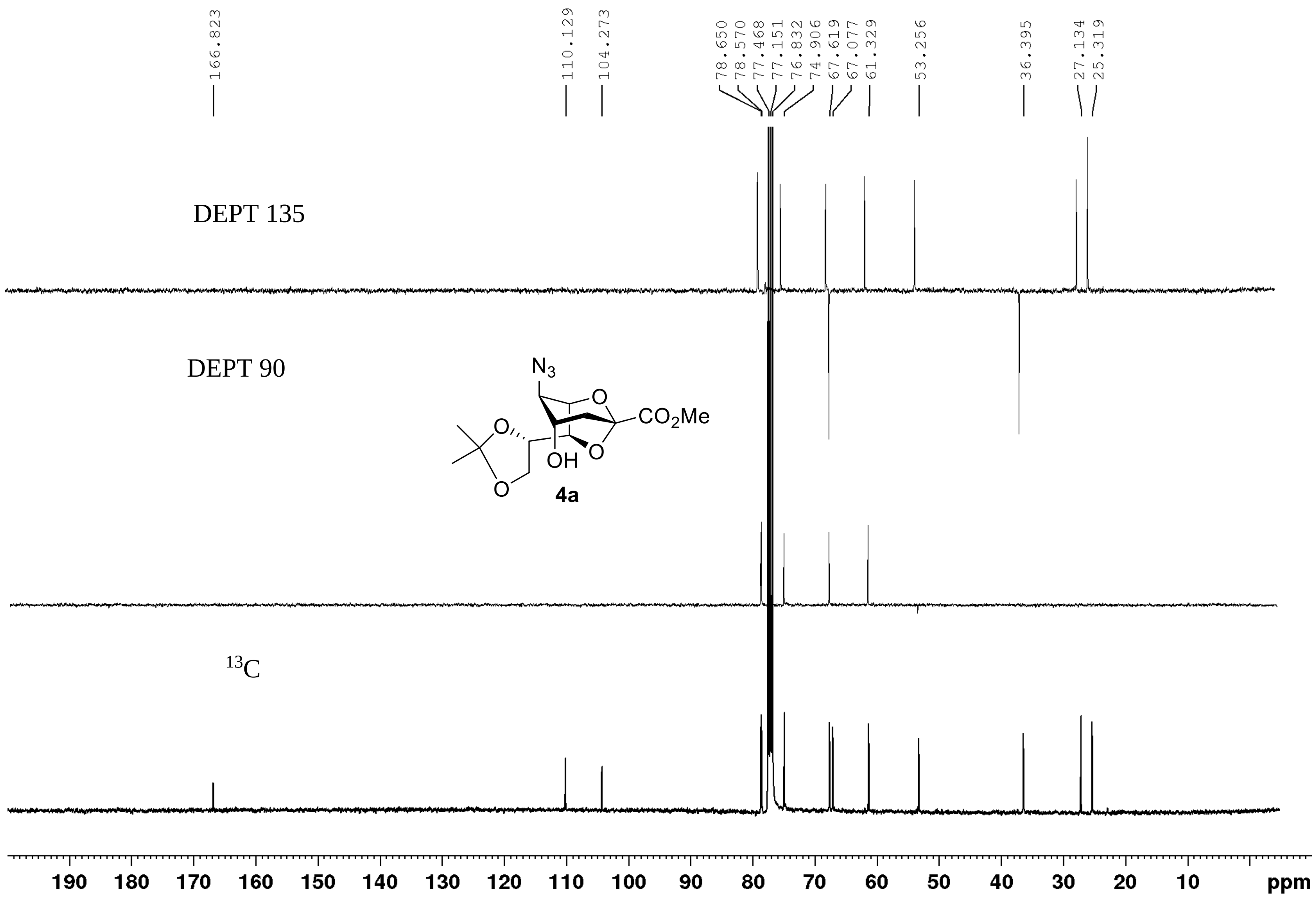

^{13}C NMR spectrum of compound **4a** (125 MHz, $CDCl_3$, 25 °C).

Elemental Composition Report

Page 1

Single Mass Analysis

Tolerance = 50.0 PPM / DBE: min = -1000.0, max = 1000.0
Element prediction: Off
Number of isotope peaks used for i-FIT = 2

Monoisotopic Mass, Even Electron Ions
15 formula(e) evaluated with 1 results within limits (all results (up to 1000) for each mass)
Elements Used:
C: 0-1000 H: 0-1000 N: 3-3 O: 7-7

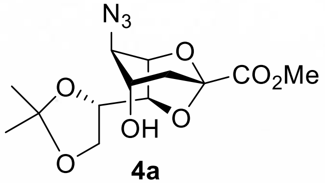

4a

KHA 371 F28-34 N3C-4OHAcetonide

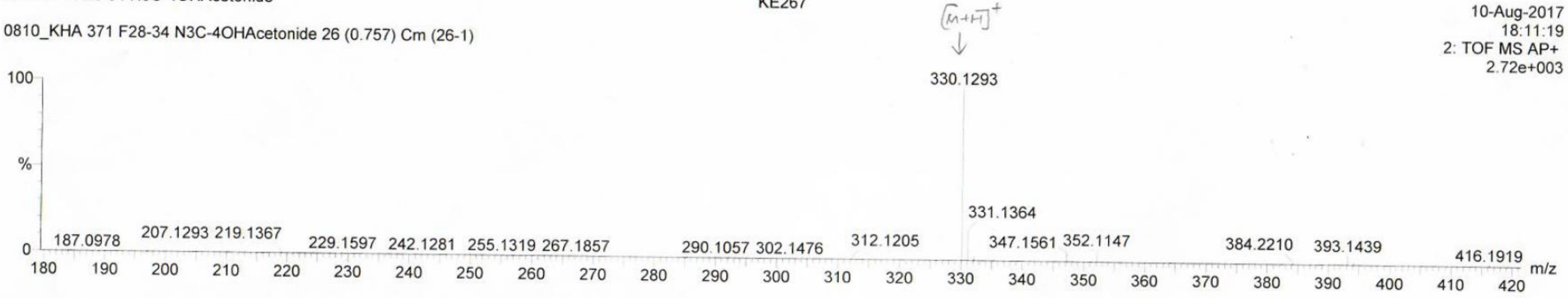

Minimum:				-1000.0			
Maximum:		5.0	50.0	1000.0			
Mass	Calc. Mass	mDa	PPM	DBE	i-FIT	i-FIT (Norm)	Formula
330.1293	330.1301	-0.8	-2.4	5.5	25.2	0.0	C13 H20 N3 O7

The HRMS spectrum of compound **4a**.

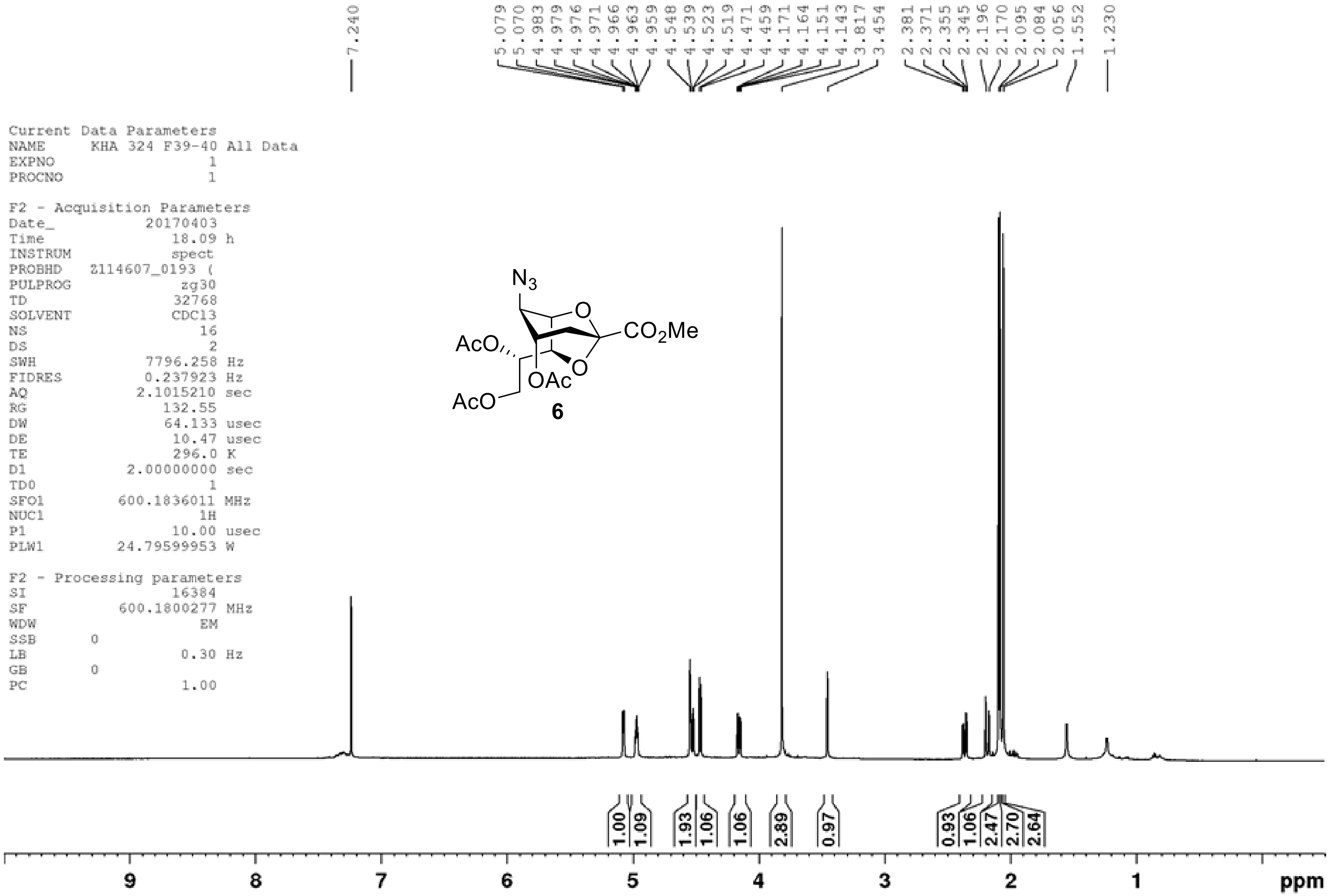

The ^{1}H NMR spectrum of compound **6**.

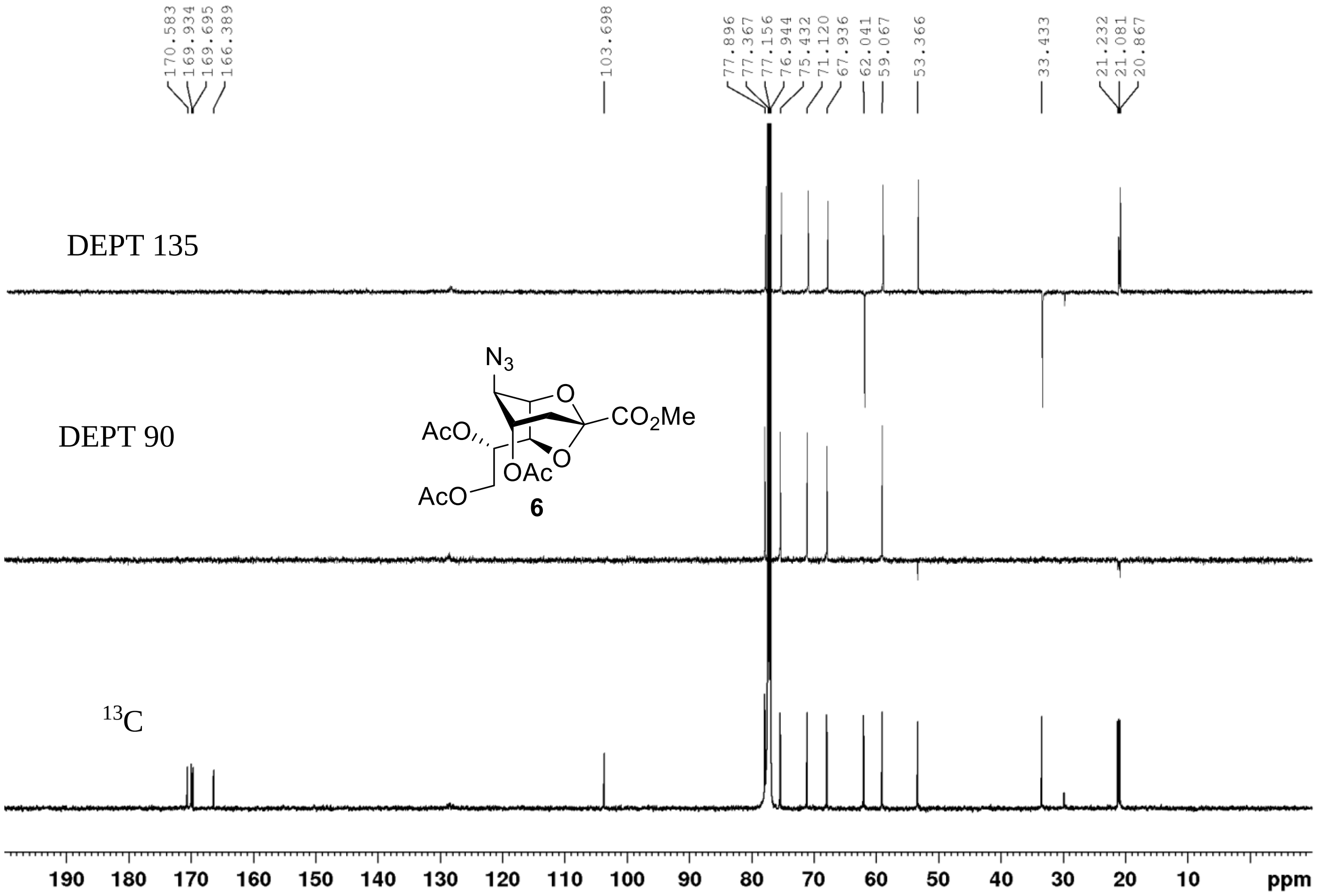

The ^{13}C NMR spectrum of compound **6**.

Elemental Composition Report **Page 1**

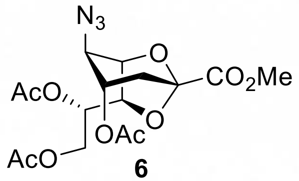

6

Single Mass Analysis

Tolerance = 30.0 PPM / DBE: min = -1000.0, max = 1000.0
Element prediction: Off
Number of isotope peaks used for i-FIT = 2

Monoisotopic Mass, Even Electron Ions
33 formula(e) evaluated with 1 results within limits (all results (up to 1000) for each mass)
Elements Used:
C: 1-2000 H: 0-2000 N: 3-3 O: 10-11 Na: 1-1

KHA 249B F15-16 3OAc N3 KE267 05-Oct-2016 17:47:45

1005_KHA 249B F15-16 3OAc N3 39 (3.134) Cm (39-1x10.000) 1: TOF MS ES+ 2.47e+003

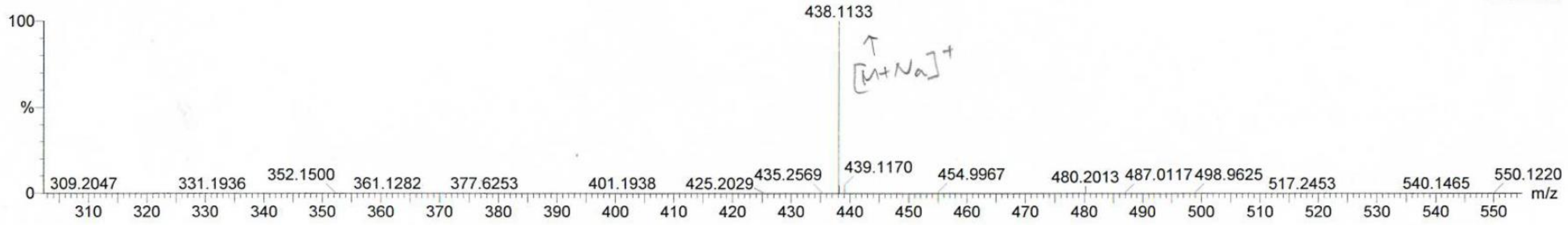

Mass	Calc. Mass	mDa	PPM	DBE	i-FIT	i-FIT (Norm)	Formula
Minimum:				-1000.0			
Maximum:		5.0	30.0	1000.0			
438.1133	438.1125	0.8	1.8	7.5	27.5	0.0	C16 H21 N3 O10 Na

The HRMS spectrum of compound **6**.

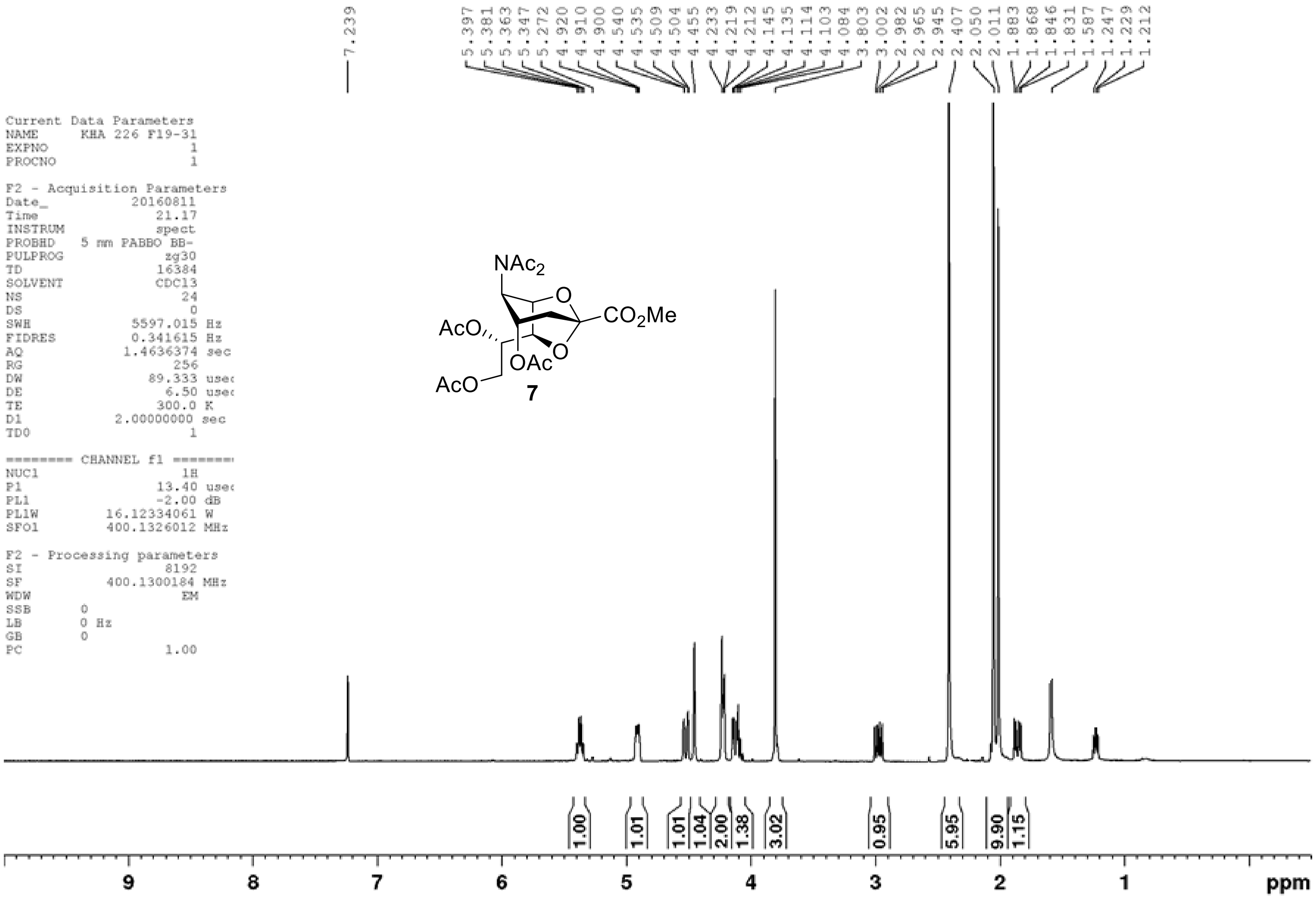

The ^{1}H NMR spectrum of compound **7**.

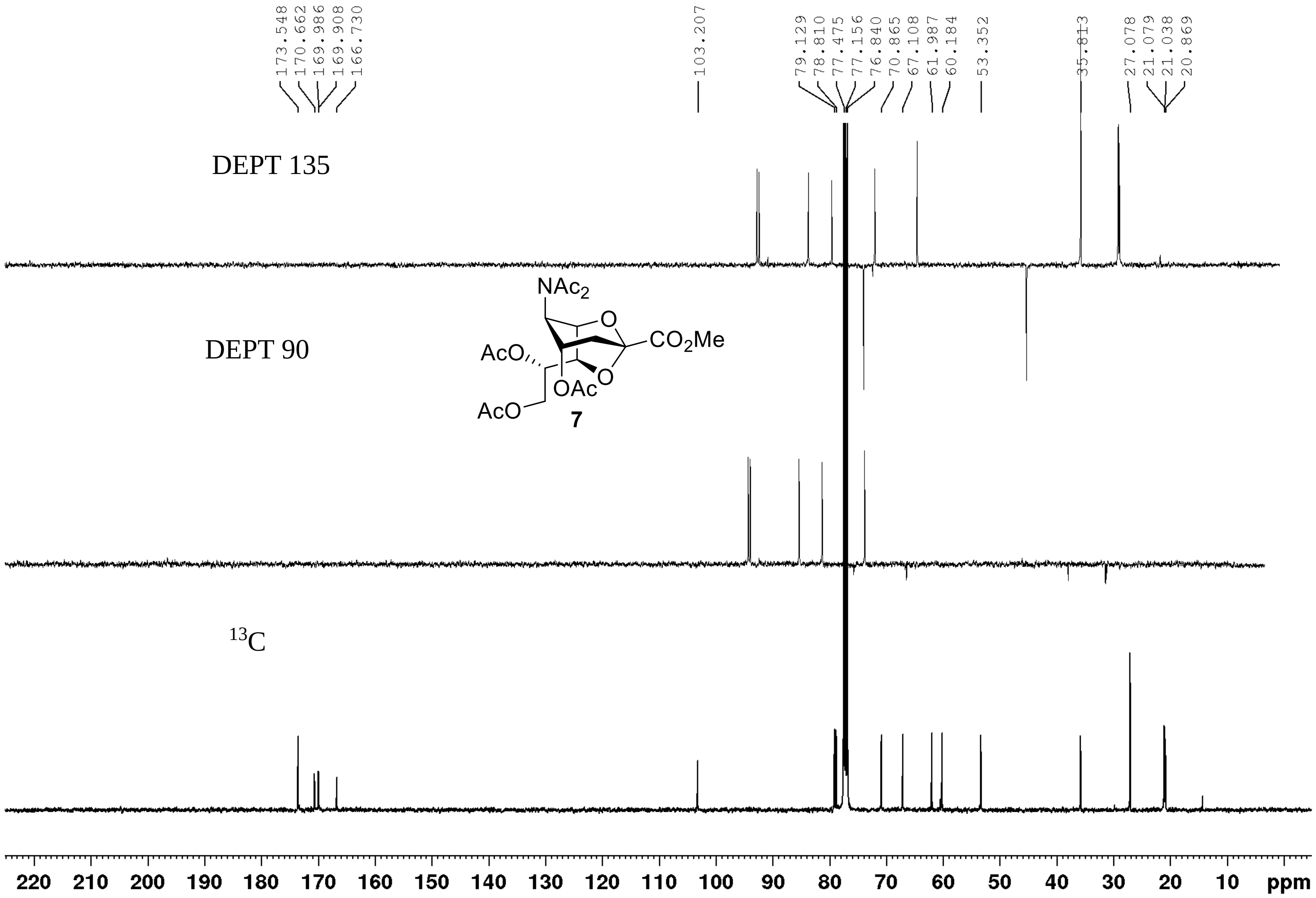

The ^{13}C NMR spectrum of compound **7**.

Elemental Composition Report Page 1

Single Mass Analysis
Tolerance = 50.0 PPM / DBE: min = -1000.0, max = 1000.0
Element prediction: Off
Number of isotope peaks used for i-FIT = 2

Monoisotopic Mass, Even Electron Ions
22 formula(e) evaluated with 1 results within limits (all results (up to 1000) for each mass)
Elements Used:
C: 1-1000 H: 0-4000 N: 1-1 O: 12-12 Na: 1-1

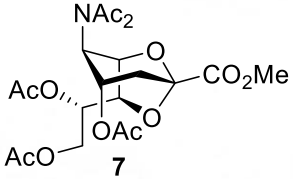

7

KHA 224 F13-16 NAc2 KE267 09-Sep-2016
17:09:02
0909_KHA 224 F13-16 NAc2 6 (0.479) Cm (6-7) 1: TOF MS ES+
5.74e+003

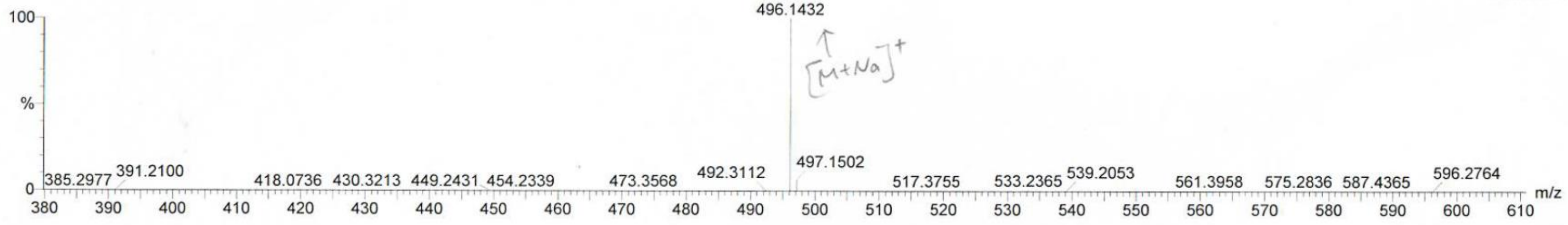

Minimum:				-1000.0			
Maximum:		5.0	50.0	1000.0			
Mass	Calc. Mass	mDa	PPM	DBE	i-FIT	i-FIT (Norm)	Formula
496.1432	496.1431	0.1	0.2	7.5	25.6	0.0	C20 H27 N O12 Na

The HRMS spectrum of compound **7**.

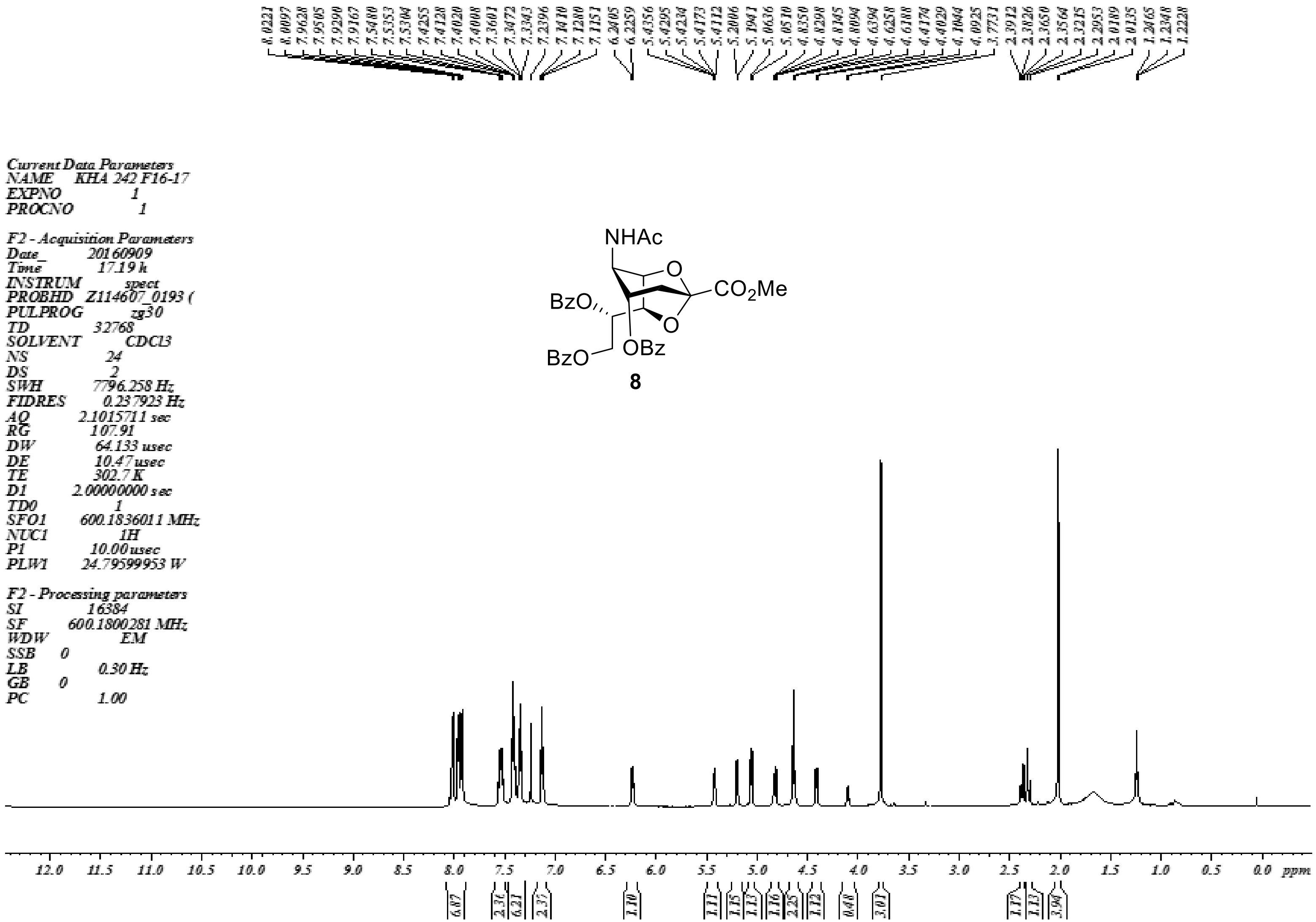

The ^{1}H NMR spectrum of compound **8**.

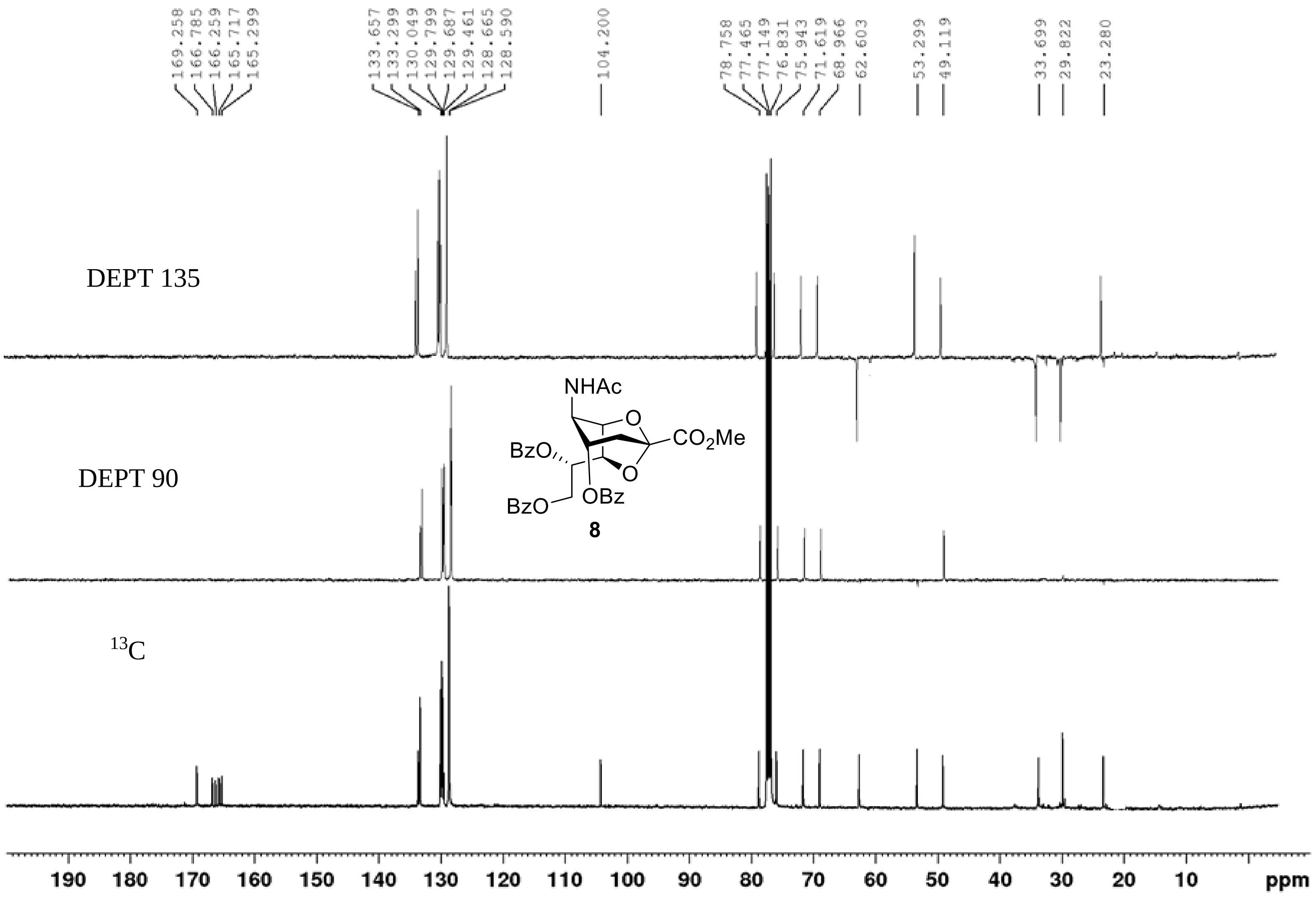

The ^{13}C NMR spectrum of compound **8**.

Elemental Composition Report **Page 1**

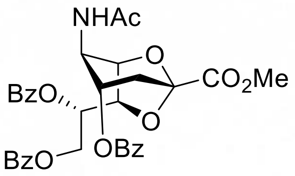

8

Single Mass Analysis
Tolerance = 50.0 PPM / DBE: min = -1000.0, max = 1000.0
Element prediction: Off
Number of isotope peaks used for i-FIT = 2

Monoisotopic Mass, Even Electron Ions
35 formula(e) evaluated with 1 results within limits (all results (up to 1000) for each mass)
Elements Used:
C: 1-1000 H: 0-4000 N: 1-1 O: 11-11 Na: 1-1

KHA 238 F14-16 3OBzNHAc KE267 09-Sep-2016 17:05:05

0909_KHA 238 F14-16 3OBzNHAc 17 (1.387) Cm (17-26x2.000) 1: TOF MS ES+ 2.11e+002

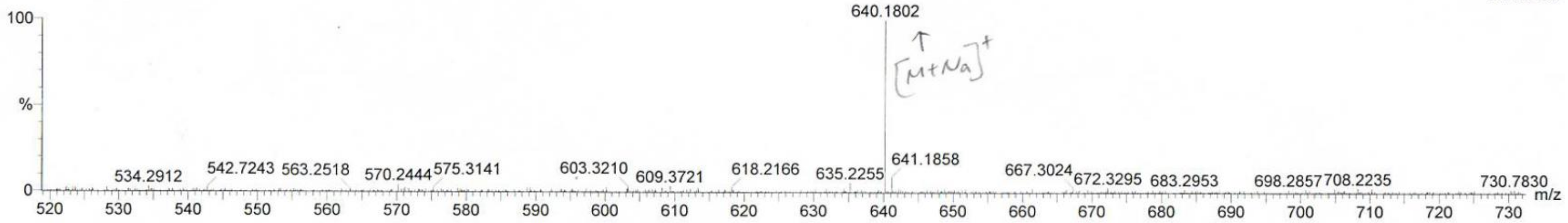

Minimum:				-1000.0			
Maximum:		5.0	50.0	1000.0			
Mass	Calc. Mass	mDa	PPM	DBE	i-FIT	i-FIT (Norm)	Formula
640.1802	640.1795	0.7	1.1	18.5	25.5	0.0	C33 H31 N O11 Na

The HRMS spectrum of compound **8**.

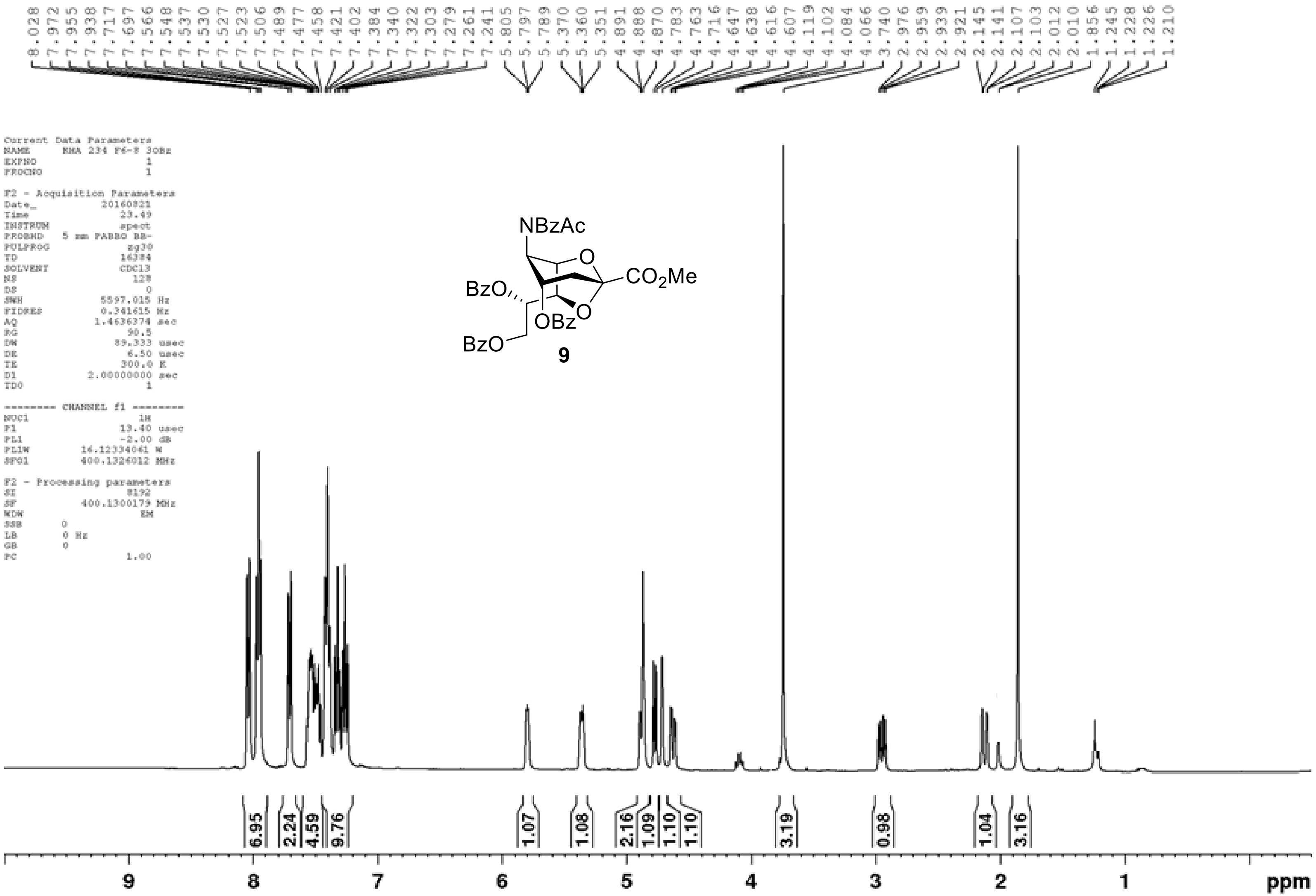

The ^{1}H NMR spectrum of compound **9**.

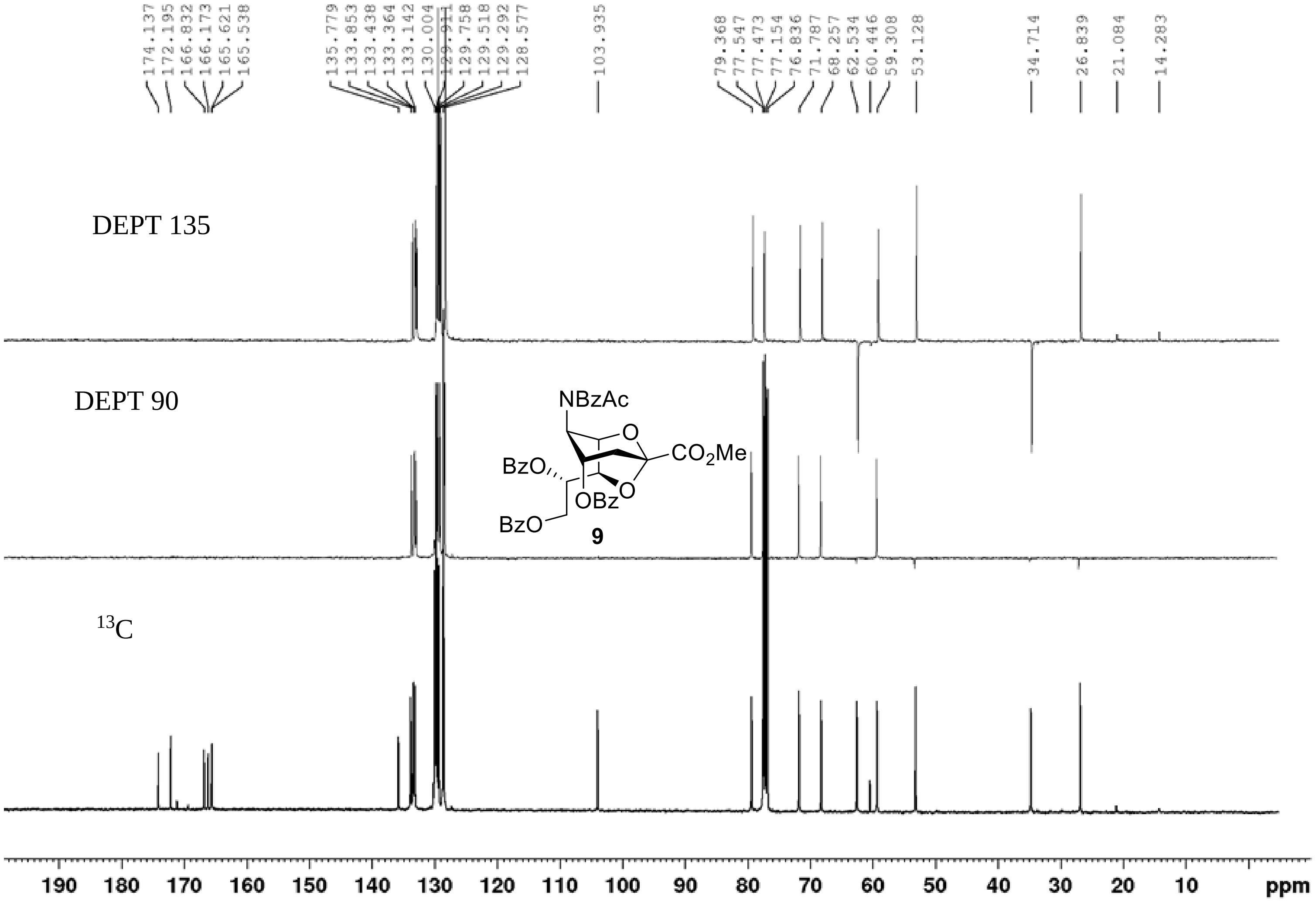

The ^{13}C NMR spectrum of compound **9**.

KHA 234 F6-8 3OBzNBzAc (HR-ESI)

Elemental Composition Report

Page 1

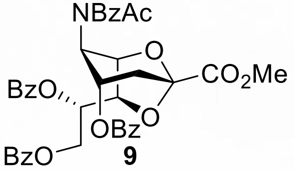

9

Single Mass Analysis

Tolerance = 4.0 PPM / DBE: min = -1000.0, max = 1000.0
Element prediction: Off
Number of isotope peaks used for i-FIT = 2

Monoisotopic Mass, Even Electron Ions
44 formula(e) evaluated with 1 results within limits (all results (up to 1000) for each mass)
Elements Used:
C: 0-10000 H: 0-10000 N: 1-1 O: 12-12 Na: 1-1

KHA 234 F6-8 3OBzNBzAc KE267 11-May-2017 14:22:53

0511_KHA 234 F6-8 3OBzNBzAc 28 (0.999) Cm (25:28) 1: TOF MS ES+ 6.67e+002

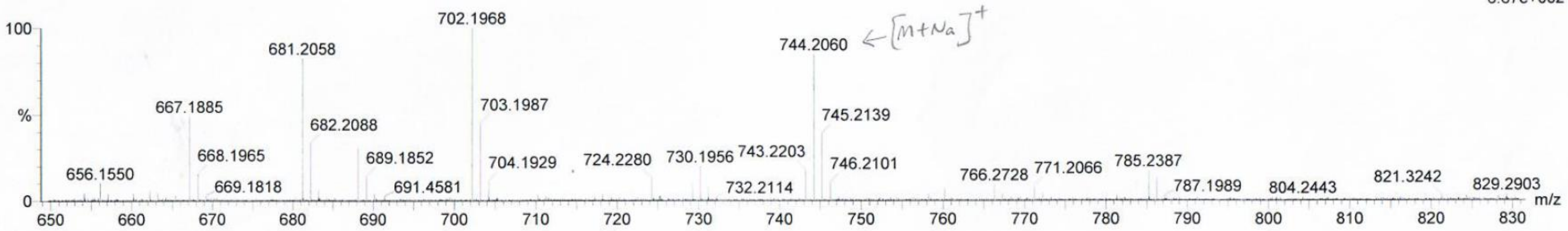

Minimum:				-1000.0			
Maximum:		5.0	4.0	1000.0			
Mass	Calc. Mass	mDa	PPM	DBE	i-FIT	i-FIT (Norm)	Formula
744.2060	744.2057	0.3	0.4	23.5	55.5	0.0	C40 H35 N O12 Na

The HRMS spectrum of compound **9**.

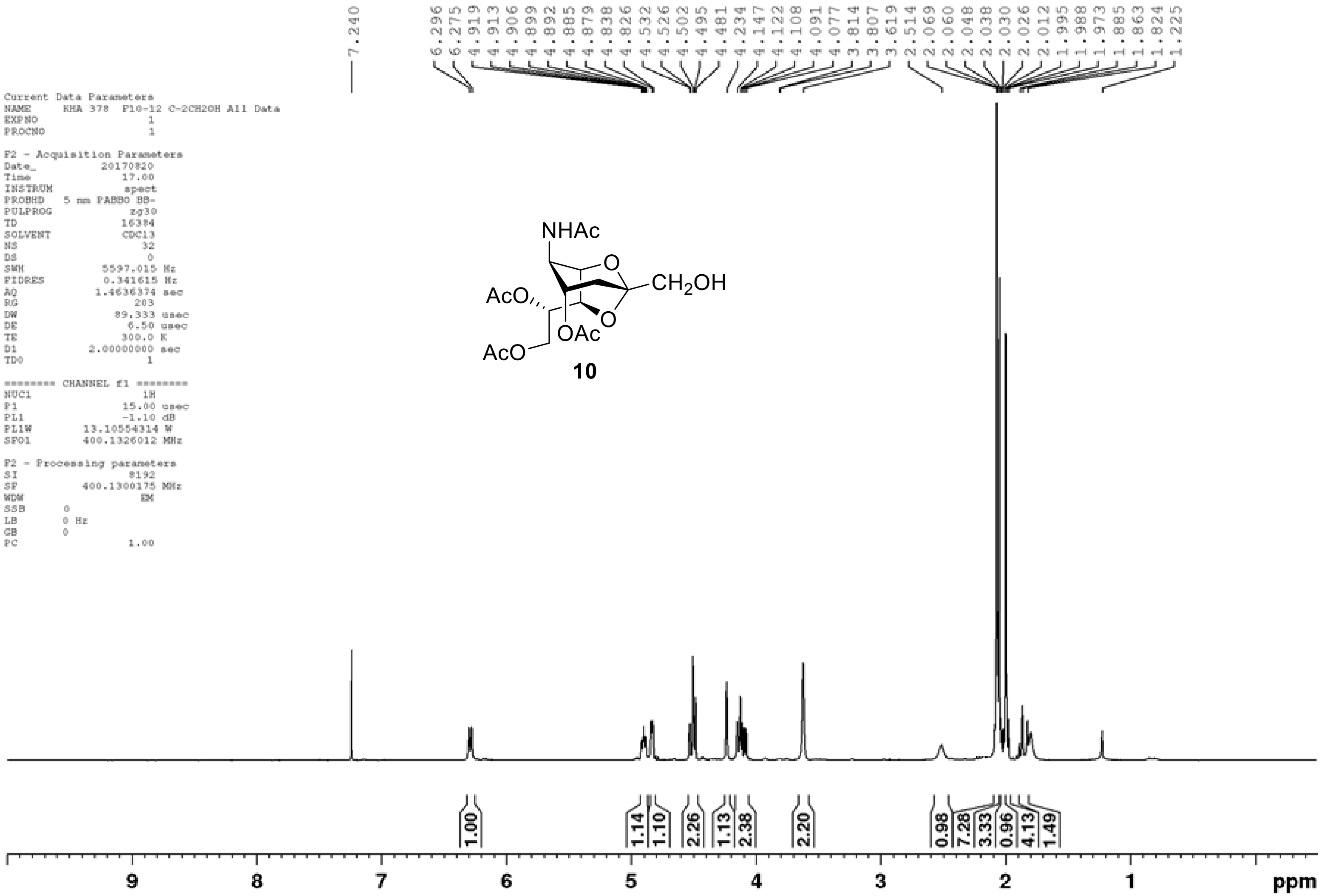

The ^{1}H NMR spectrum of compound **10**.

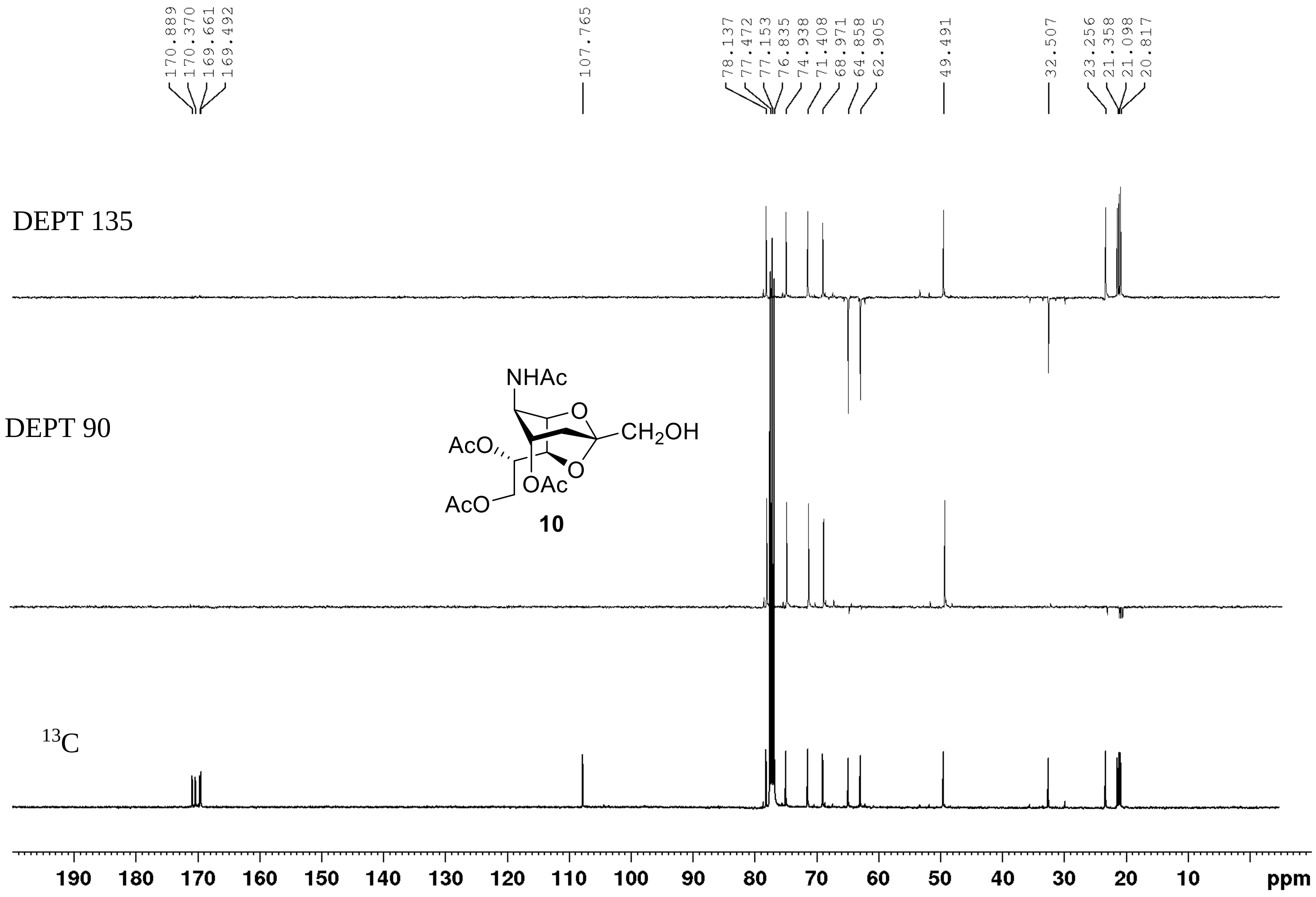

The ^{13}C NMR spectrum of compound **10**.

KHA 378 F10-12C-2CH2OH (HR-ESI)

Elemental Composition Report

Page 1

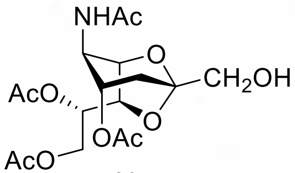

10

Single Mass Analysis

Tolerance = 5.0 PPM / DBE: min = -1.5, max = 50.0
Element prediction: Off
Number of isotope peaks used for i-FIT = 3

Monoisotopic Mass, Even Electron Ions
8 formula(e) evaluated with 1 results within limits (up to 50 best isotopic matches for each mass)
Elements Used:
C: 0-500 H: 0-1000 N: 1-1 O: 10-10 Na: 0-1
KHA 378 F10-11 C-2CH2OH
0828_KHA 378 F10-11 C-2CH2OH 49 (1.786)

1: TOF MS ES+
3.05e+004

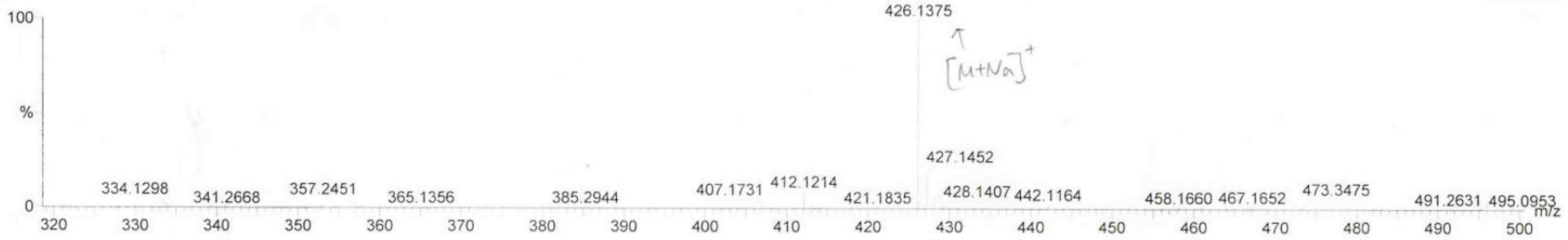

Minimum:				-1.5			
Maximum:		5.0	5.0	50.0			
Mass	Calc. Mass	mDa	PPM	DBE	i-FIT	i-FIT (Norm)	Formula
426.1375	426.1376	-0.1	-0.2	5.5	84.5	0.0	C17 H25 N O10 Na

The HRMS spectrum of compound **10**.

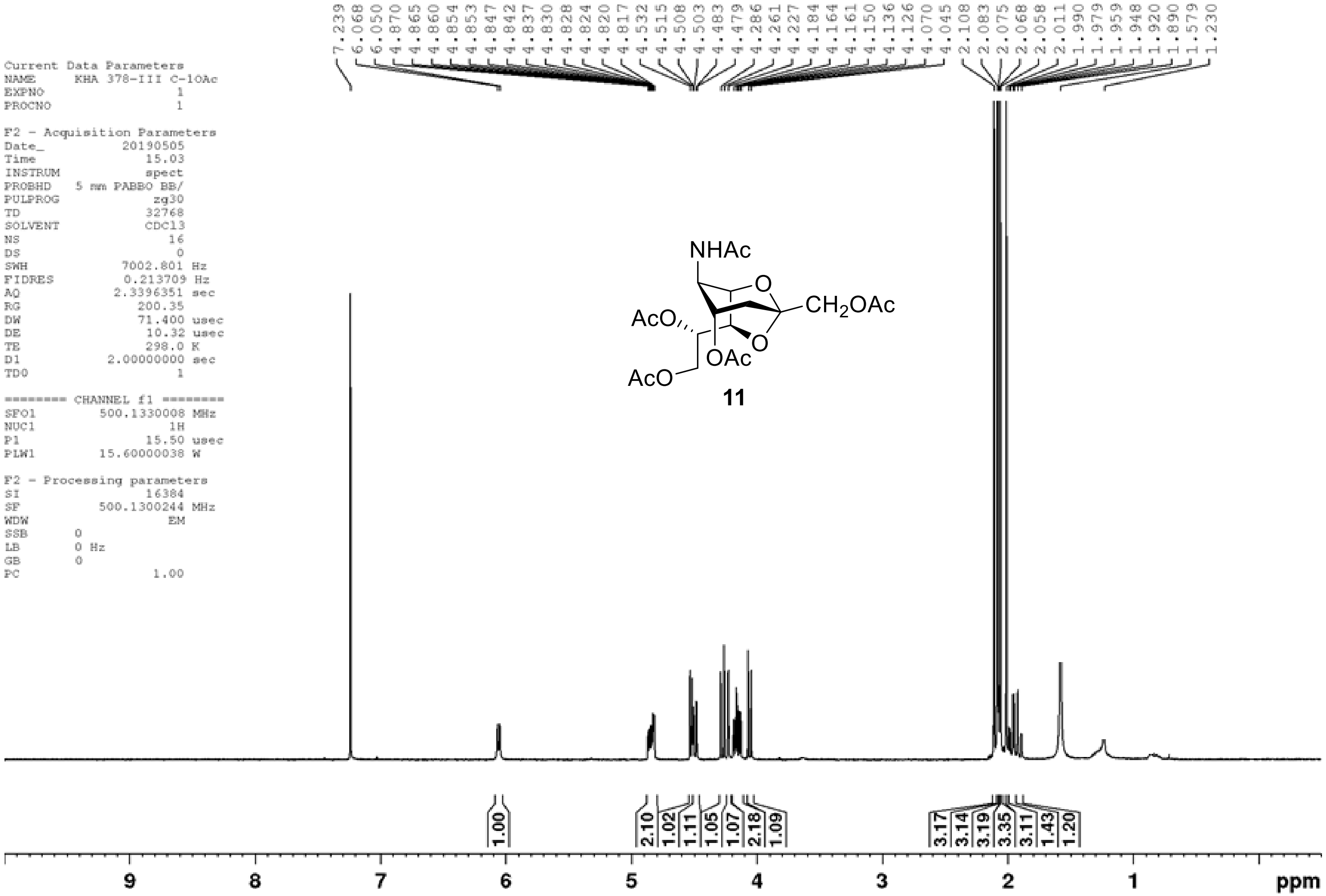

The ^{1}H NMR spectrum of compound **11**.

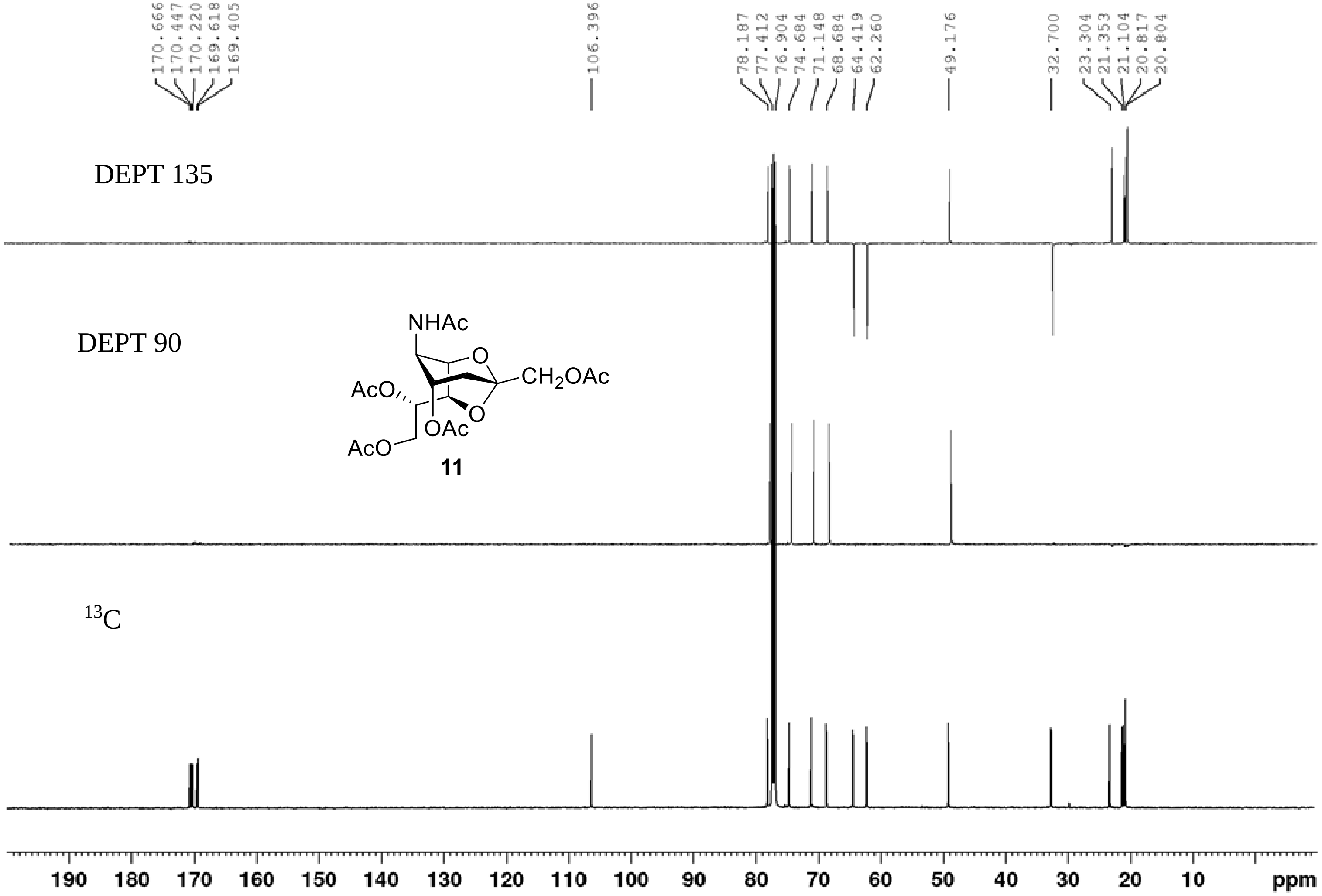

The ^{13}C NMR spectrum of compound **11**.

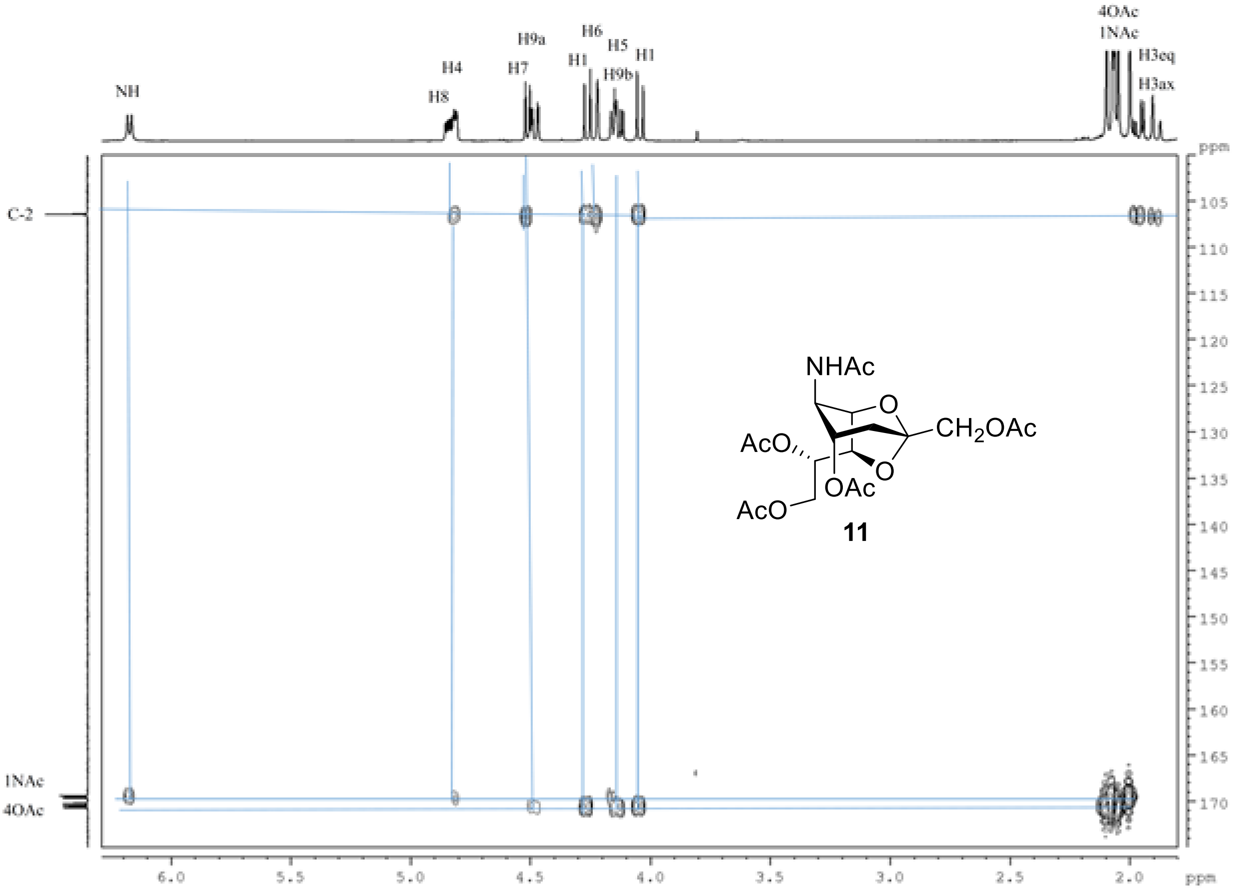

The HMBC NMR spectrum of compound **11**.

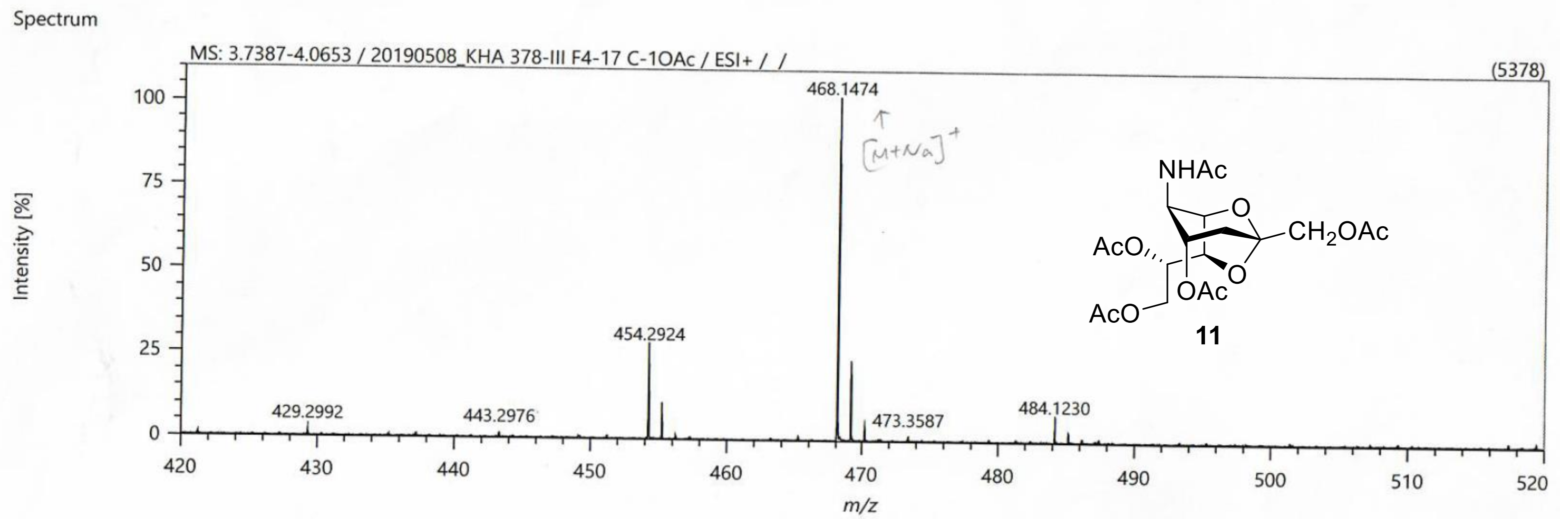

Elemental Composition

Parameters	
Tolerance:	±20.00 ppm
Electron:	Odd/Even
Charge:	+1
DBE:	-1.5 - 999.0

Elements Set 1:

Symbol	C	H	O	Na	Cl	F	N	S
Min	0	0	11	1	0	0	1	0
Max	400	1000	11	1	0	0	1	0

Results

Mass	Formula	Calculated Mass	Mass Difference [mDa]	Mass Difference [ppm]	DBE
468.14741	C19 H27 N O11 Na	468.14763	-0.22	-0.47	6.5

1NAc

4OAc

The HRMS spectrum of compound **11**.

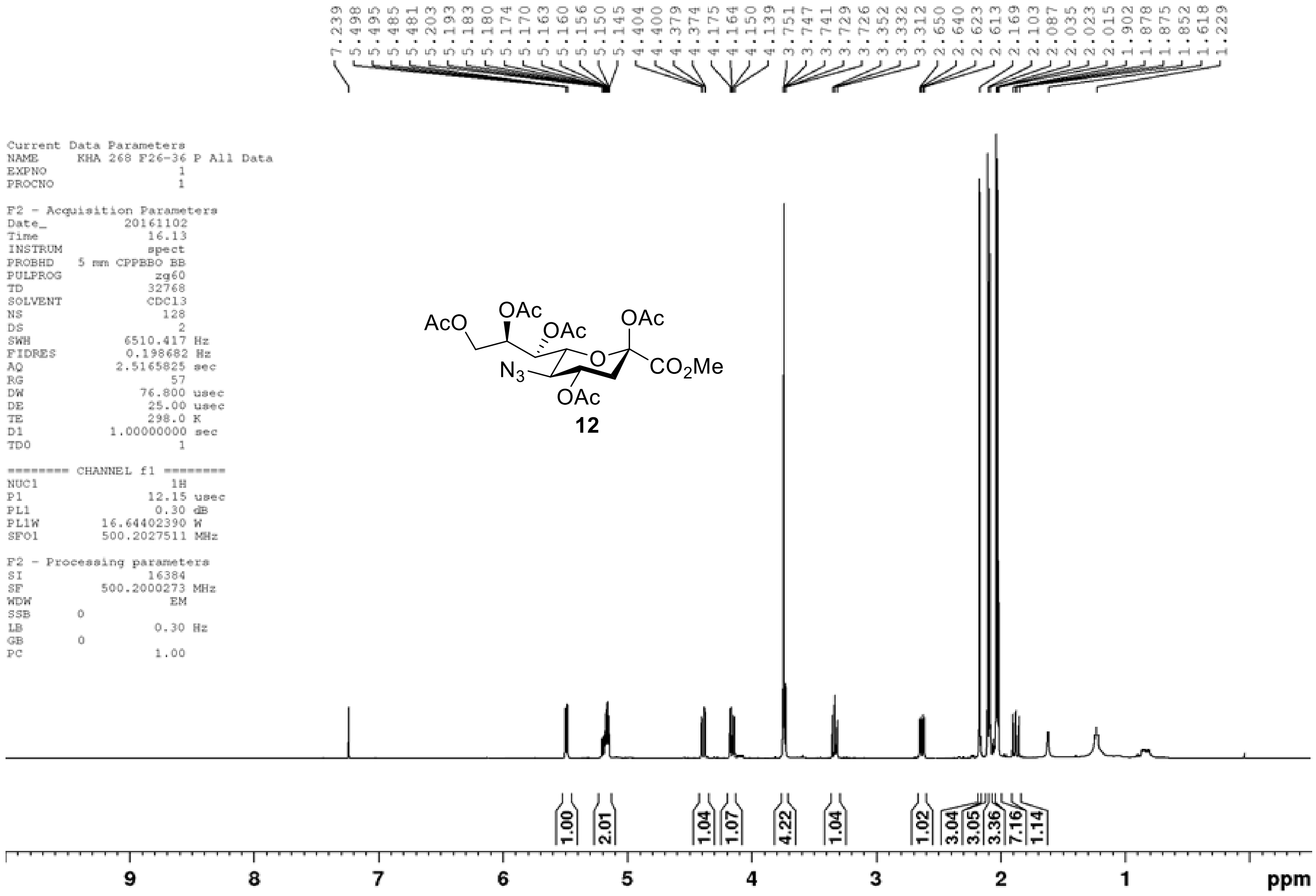

The ^1H NMR spectrum of compound **12**.

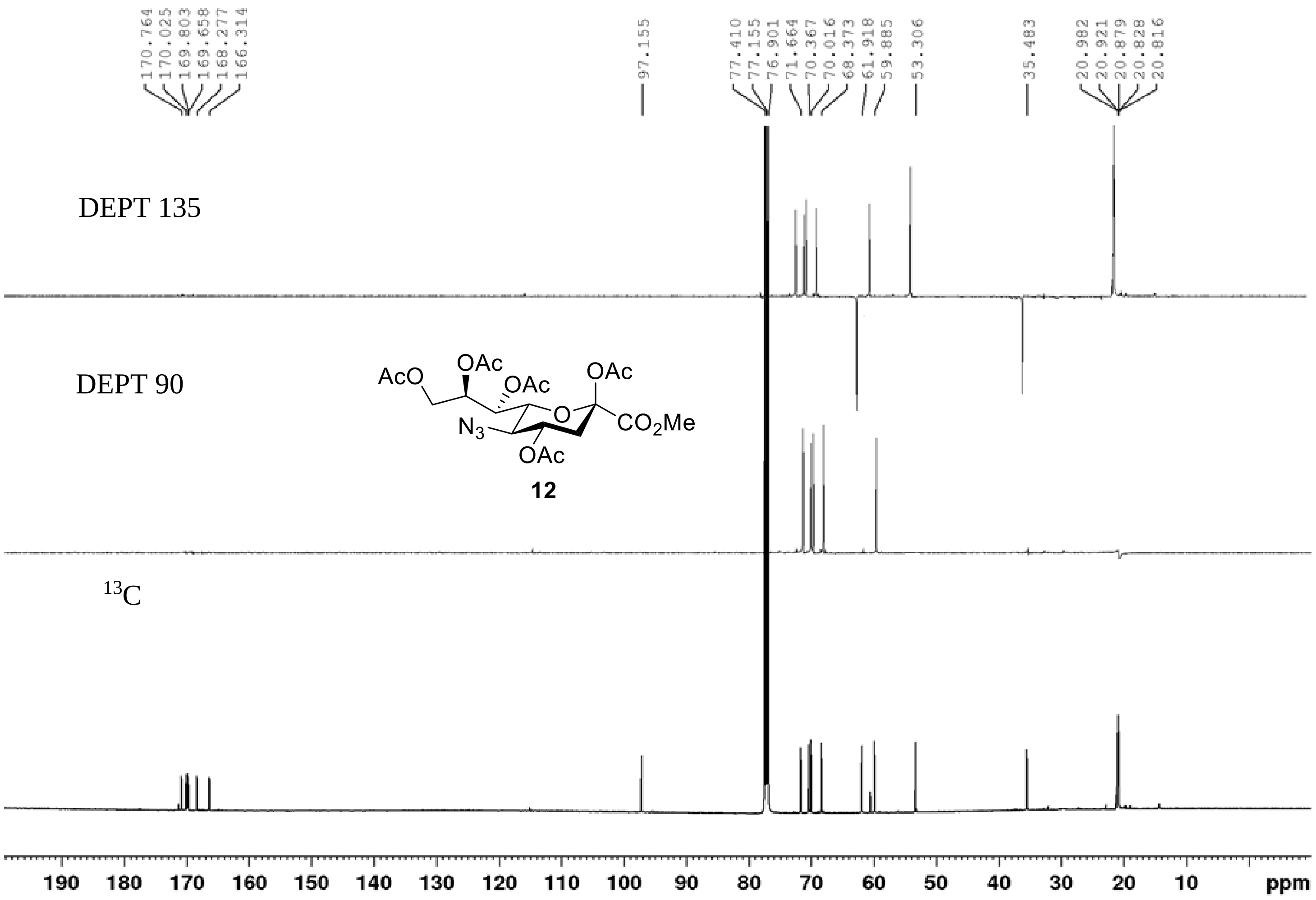

The ^{13}C NMR spectrum of compound **12**.

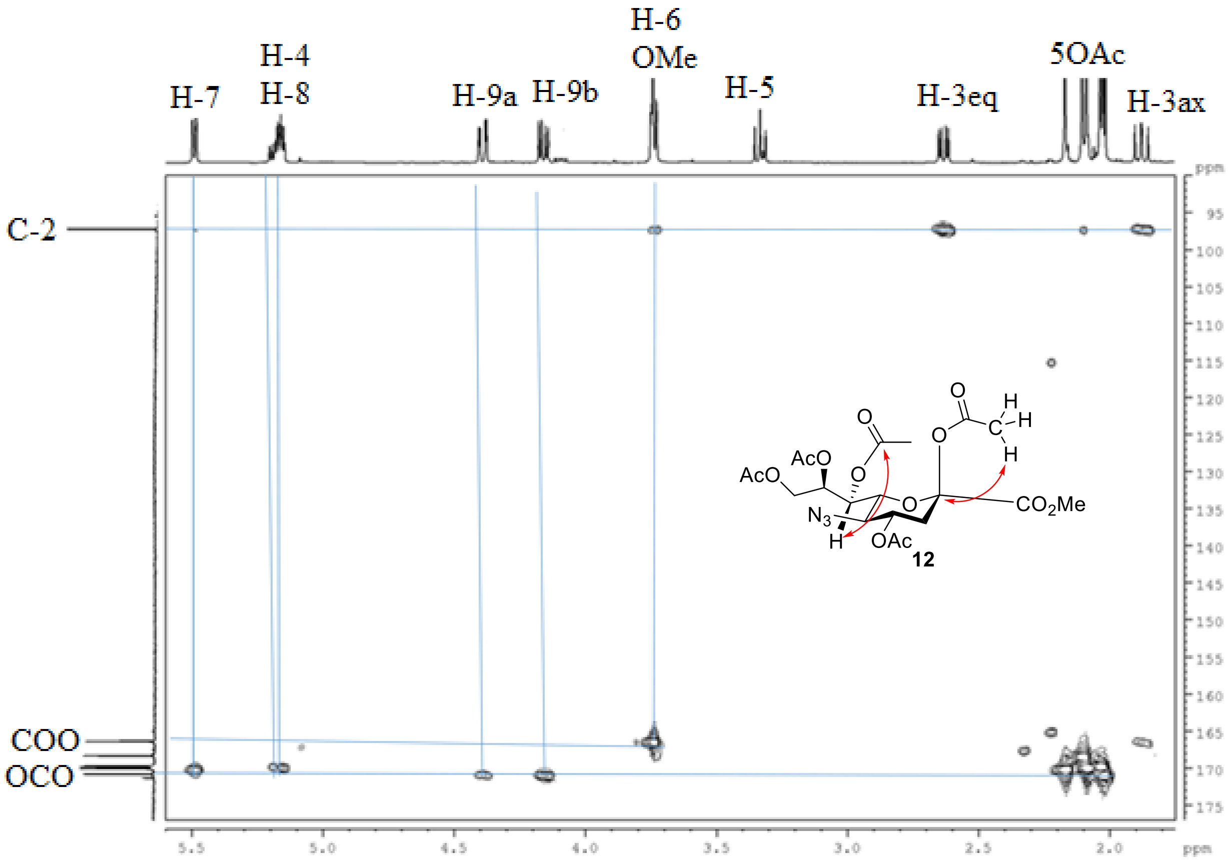

The HMBC NMR spectrum of compound **12**.

Elemental Composition Report Page 1

Single Mass Analysis
Tolerance = 100.0 PPM / DBE: min = -1000.0, max = 1000.0
Element prediction: Off
Number of isotope peaks used for i-FIT = 2

Monoisotopic Mass, Even Electron Ions
23 formula(e) evaluated with 1 results within limits (all results (up to 1000) for each mass)
Elements Used:
C: 0-1000 H: 0-1000 N: 3-3 O: 13-13 Na: 1-1

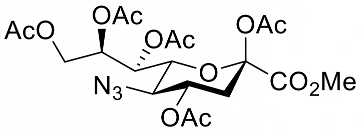

12

KHA 268 F26-36 5OAc N3 KE267 08-Nov-2016 16:02:21

1108_KHA 268 F26-36 5OAc N3 39 (3.135) Cm (39:41) 1: TOF MS ES+ 7.07e+004

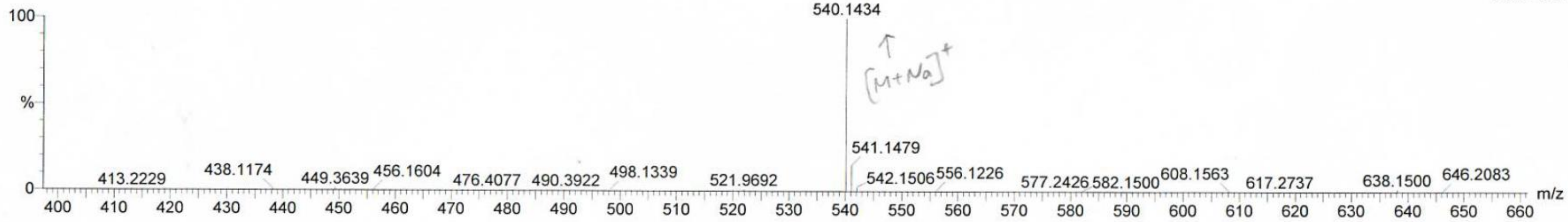

Minimum:				-1000.0			
Maximum:		5.0	100.0	1000.0			
Mass	Calc. Mass	mDa	PPM	DBE	i-FIT	i-FIT (Norm)	Formula
540.1434	540.1442	-0.8	-1.5	8.5	27.4	0.0	C20 H27 N3 O13 Na

The HRMS spectrum of compound **12**.

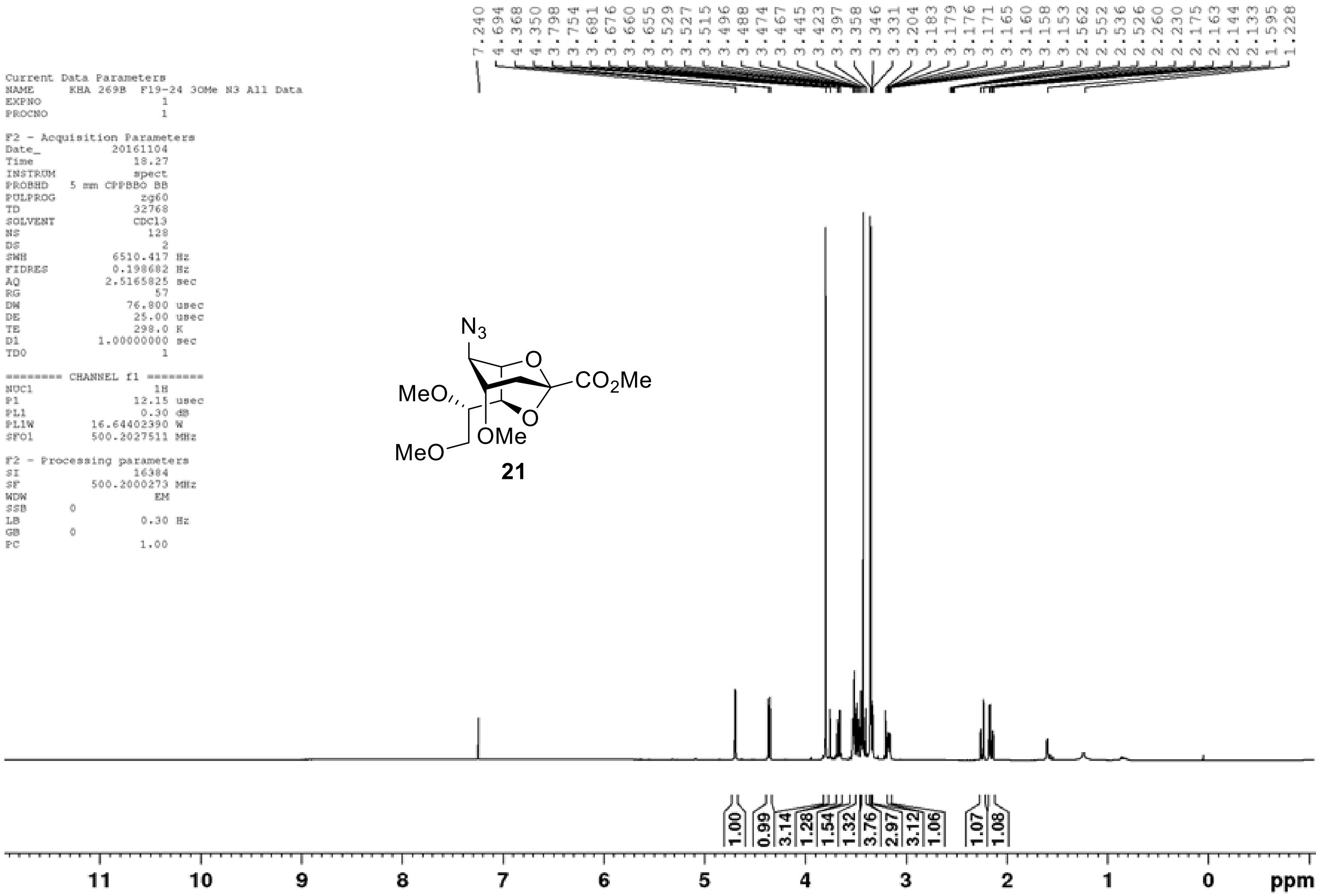

The ^1H NMR spectrum of compound **21**.

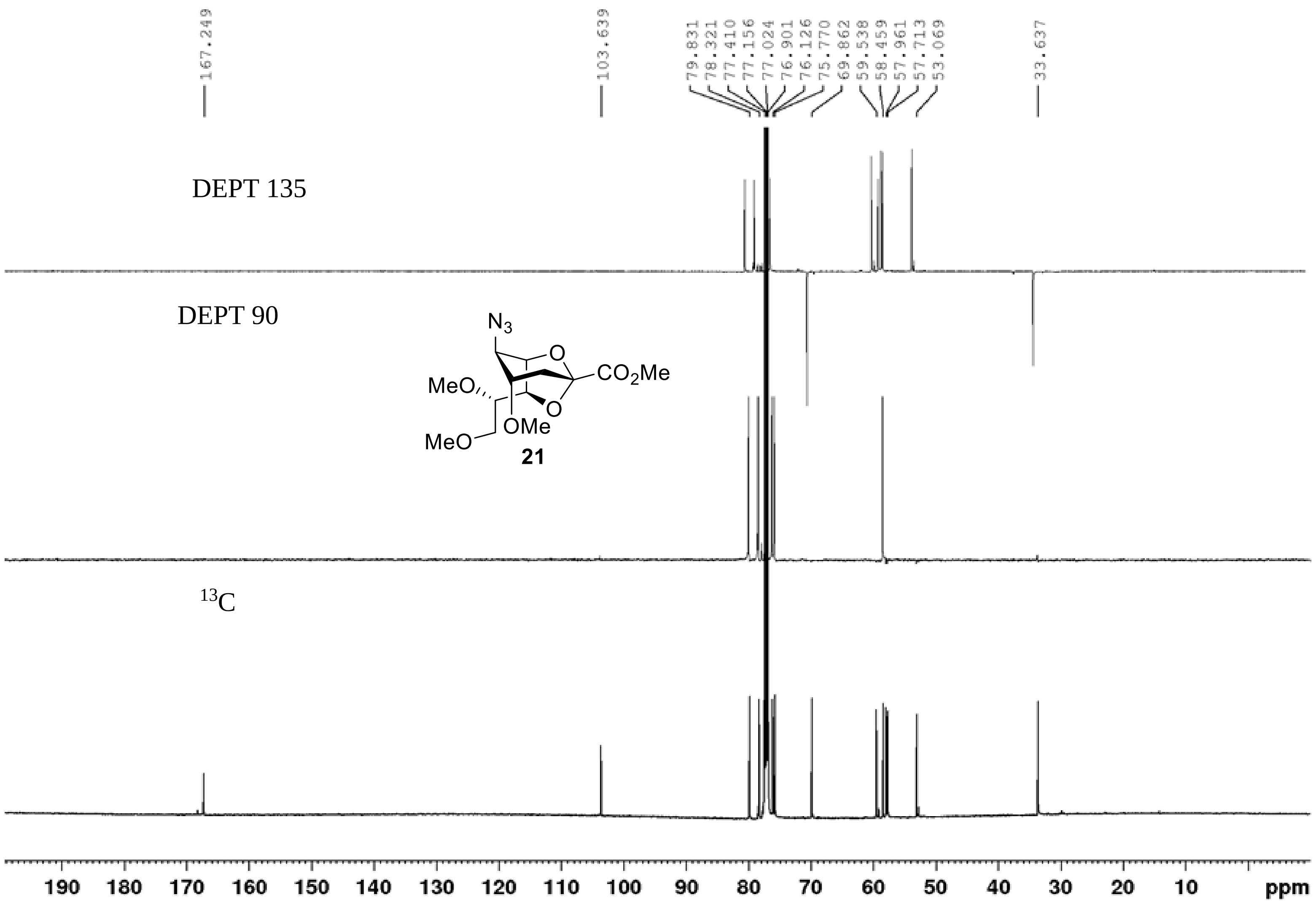

The ^{13}C NMR spectrum of compound **21**.

Elemental Composition Report **Page 1**

Single Mass Analysis
Tolerance = 50.0 PPM / DBE: min = -1000.0, max = 1000.0
Element prediction: Off
Number of isotope peaks used for i-FIT = 2

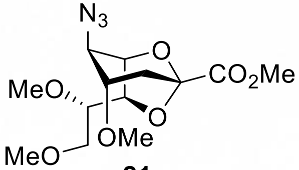

21

Monoisotopic Mass, Even Electron Ions
15 formula(e) evaluated with 1 results within limits (all results (up to 1000) for each mass)
Elements Used:
C: 0-1000 H: 0-1000 N: 3-3 O: 7-7 Na: 1-1

KHA 269B F19-243OMe N3 KE267 08-Nov-2016 16:57:10

1108_KHA 269B F19-243OMe N3 42 (3.395) Cm (42-5) 1: TOF MS ES+ 1.31e+004

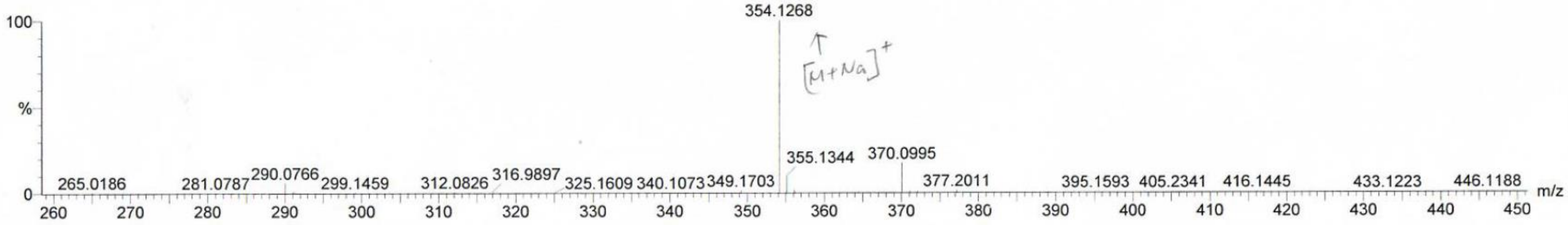

Minimum:				-1000.0			
Maximum:		5.0	50.0	1000.0			

Mass	Calc. Mass	mDa	PPM	DBE	i-FIT	i-FIT (Norm)	Formula
354.1268	354.1277	-0.9	-2.5	4.5	19.2	0.0	C13 H21 N3 O7 Na

The HRMS spectrum of compound **21**.

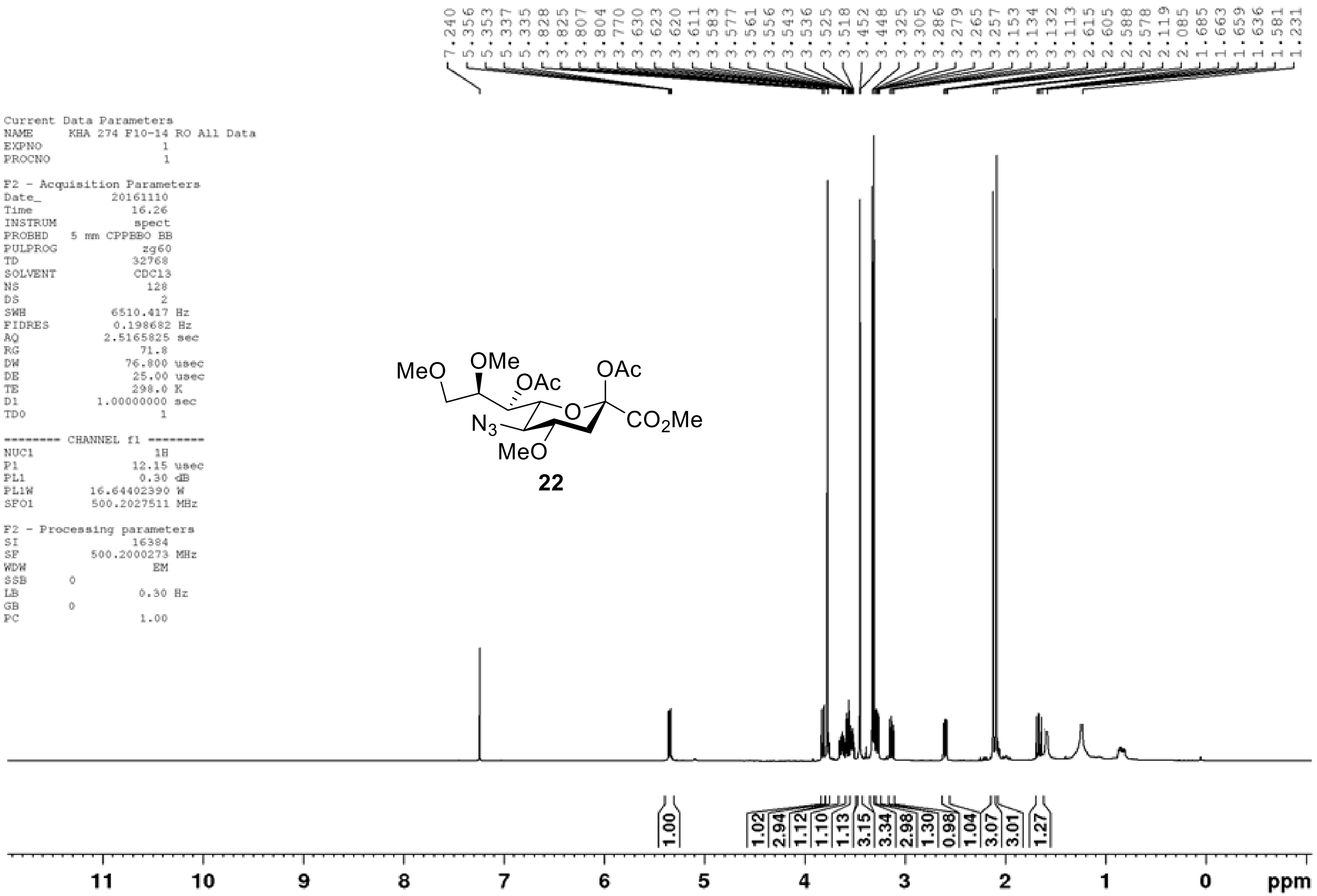

The ^1H NMR spectrum of compound **22**.

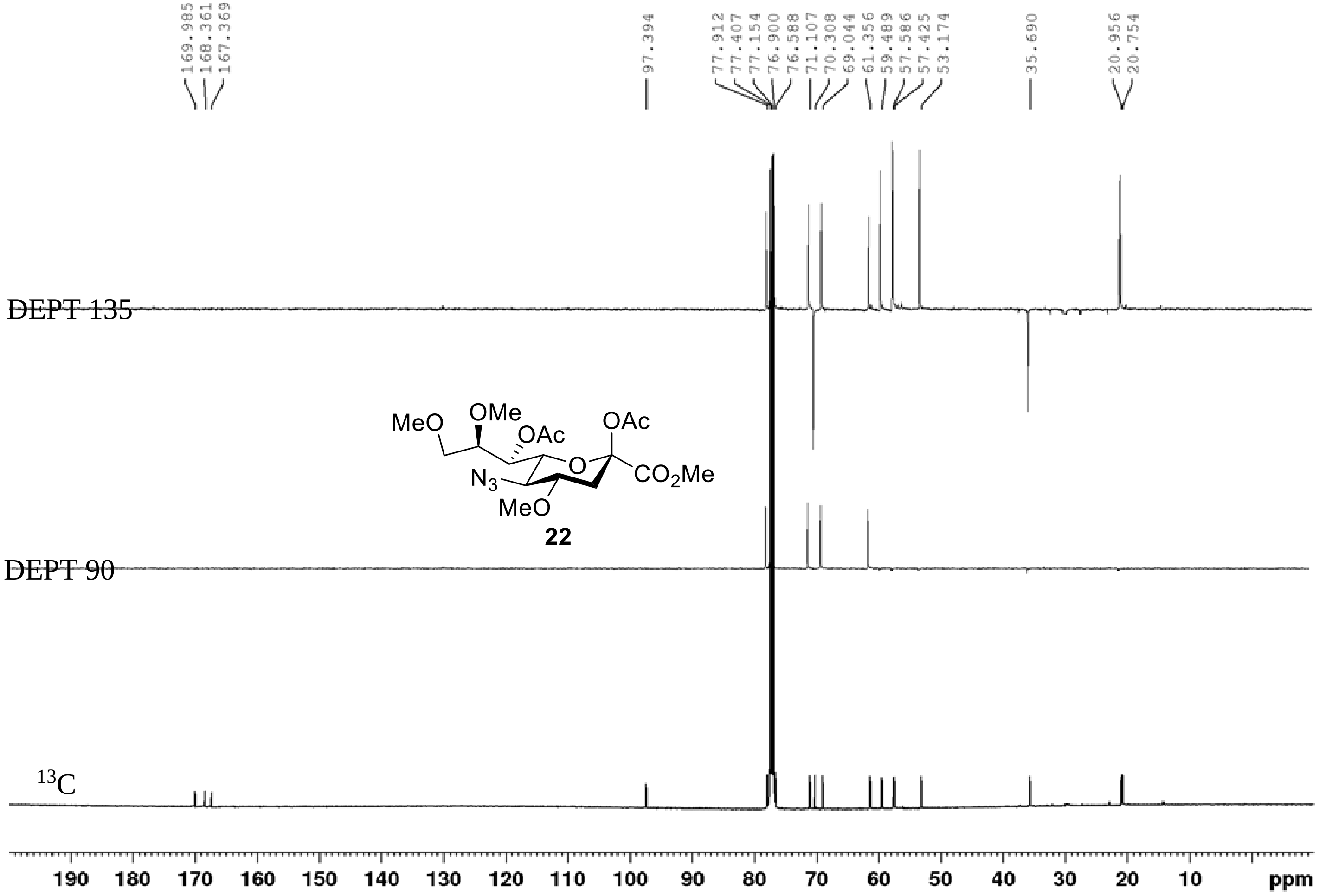

The ^{13}C NMR spectrum of compound **22**.

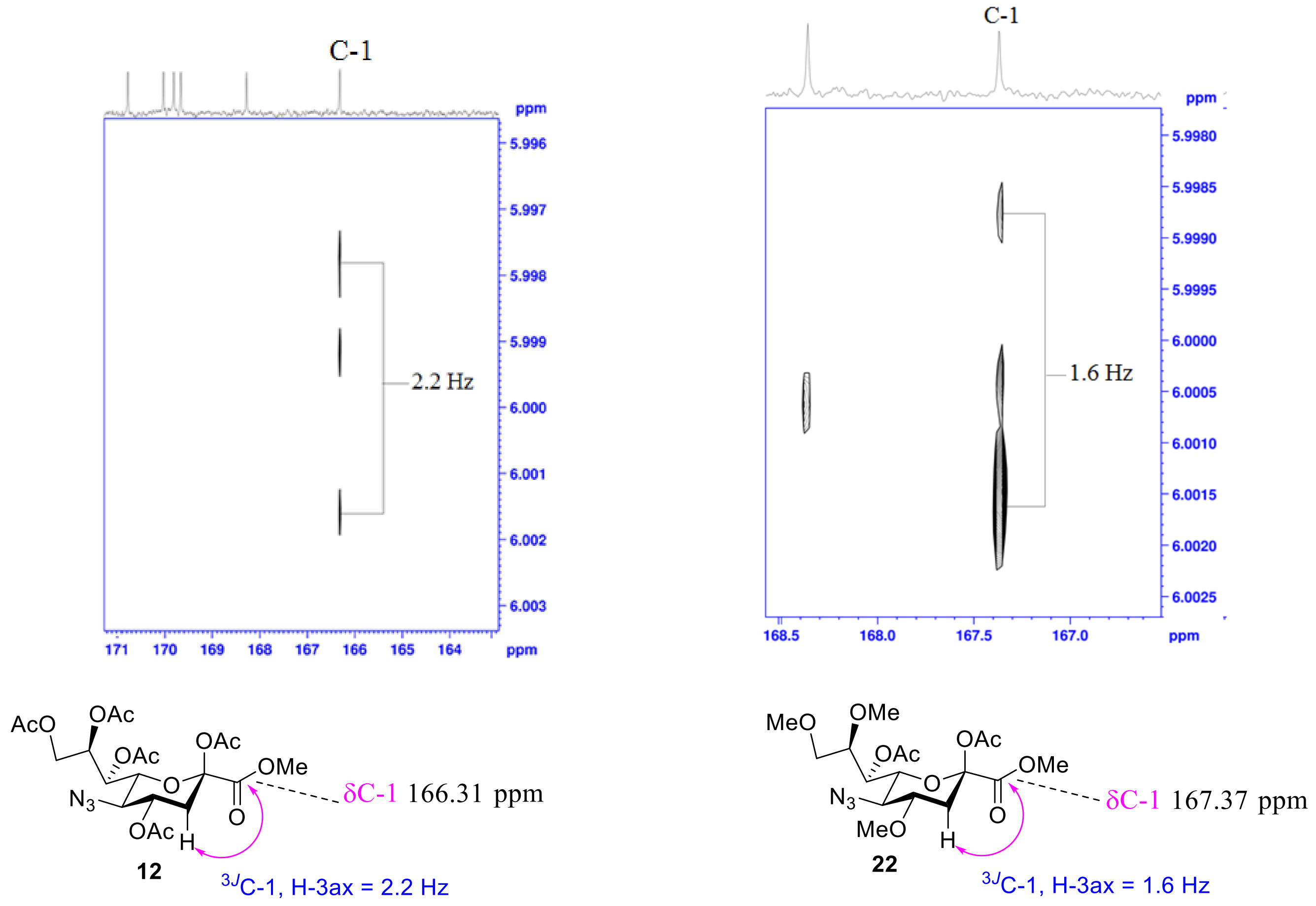

The 2D Selective Heteronuclear *J*-Resolved Spectrum of compounds **12** and **22**.

Elemental Composition Report **Page 1**

Single Mass Analysis
Tolerance = 20.0 PPM / DBE: min = -1000.0, max = 1000.0
Element prediction: Off
Number of isotope peaks used for i-FIT = 2

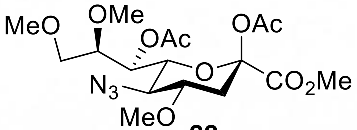

22

Monoisotopic Mass, Even Electron Ions
20 formula(e) evaluated with 1 results within limits (all results (up to 1000) for each mass)
Elements Used:
C: 0-1000 H: 0-1000 N: 3-3 O: 10-10 Na: 1-1

KHA 274 F10-14RO KE267 17-Nov-2016 17:33:59

1117_KHA 274 F10-14RO 56 (4.495) Cm (56-1x20.000) 1: TOF MS ES+ 2.05e+004

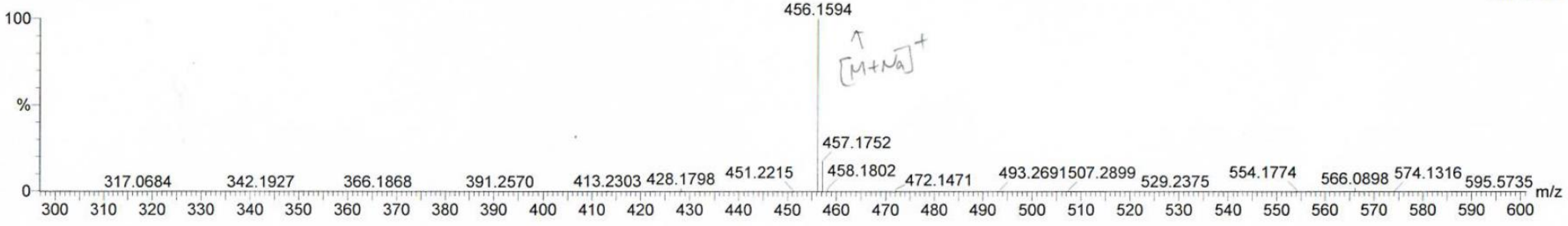

Minimum:				-1000.0			
Maximum:		5.0	20.0	1000.0			
Mass	Calc. Mass	mDa	PPM	DBE	i-FIT	i-FIT (Norm)	Formula
456.1594	456.1594	0.0	0.0	5.5	24.5	0.0	C17 H27 N3 O10 Na

The HRMS spectrum of compound **22**.

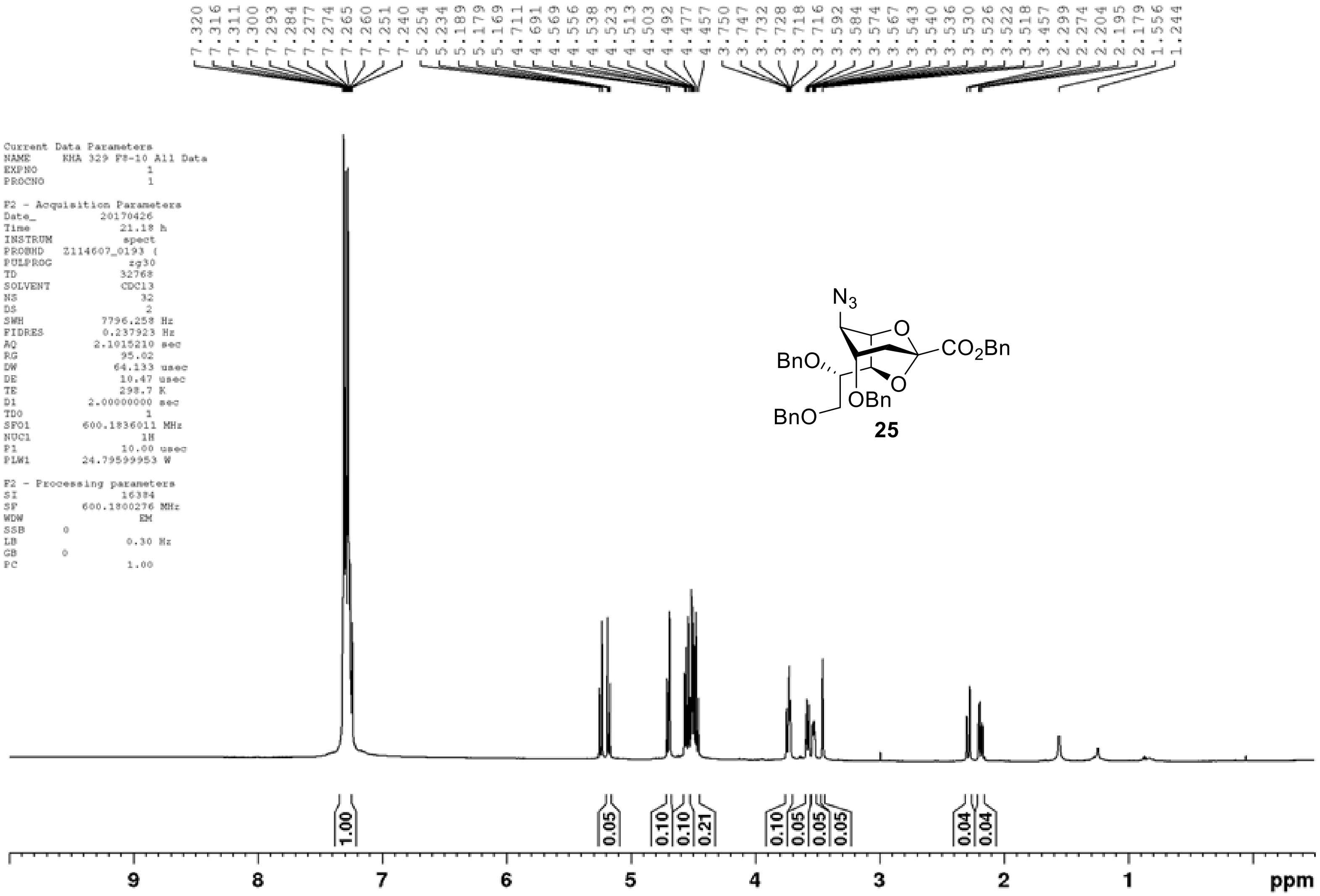

The ^{1}H NMR of compound **25**.

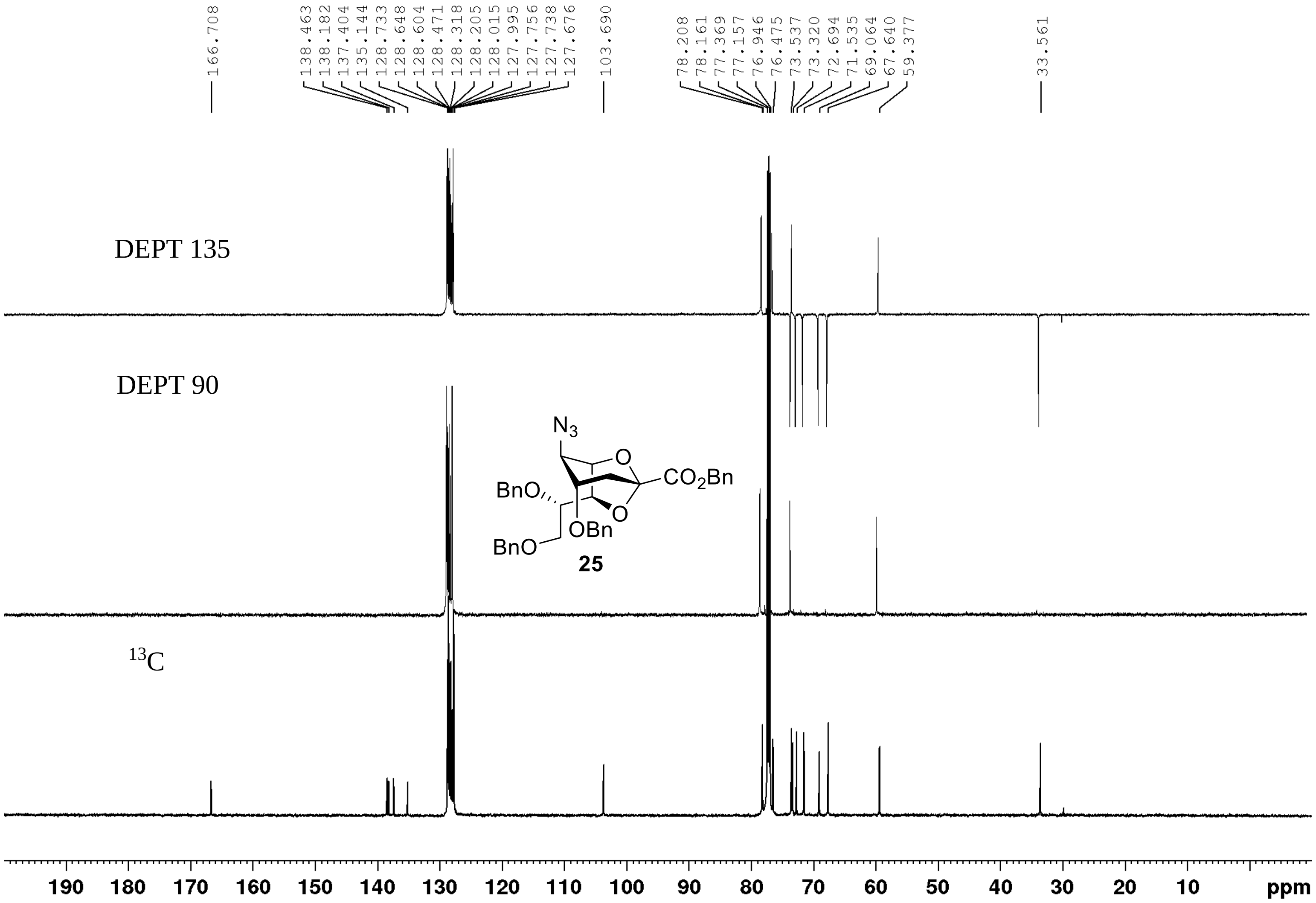

The ^{13}C NMR spectrum of compound **25**.

Elemental Composition Report Page 1

Single Mass Analysis
Tolerance = 5.0 PPM / DBE: min = -1000.0, max = 1000.0
Element prediction: Off
Number of isotope peaks used for i-FIT = 2

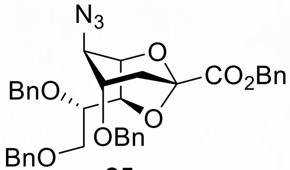

25

Monoisotopic Mass, Even Electron Ions
41 formula(e) evaluated with 1 results within limits (all results (up to 1000) for each mass)
Elements Used:
C: 0-10000 H: 0-10000 N: 3-3 O: 7-7 Na: 1-1
KHA 329 F8-10 4OBnN3 KE267 09-May-2017
13:46:36
0509_KHA 329 F8-10 4OBnN3 147 (5.331) Cm (147-1x10.000) 1: TOF MS ES+
1.10e+003

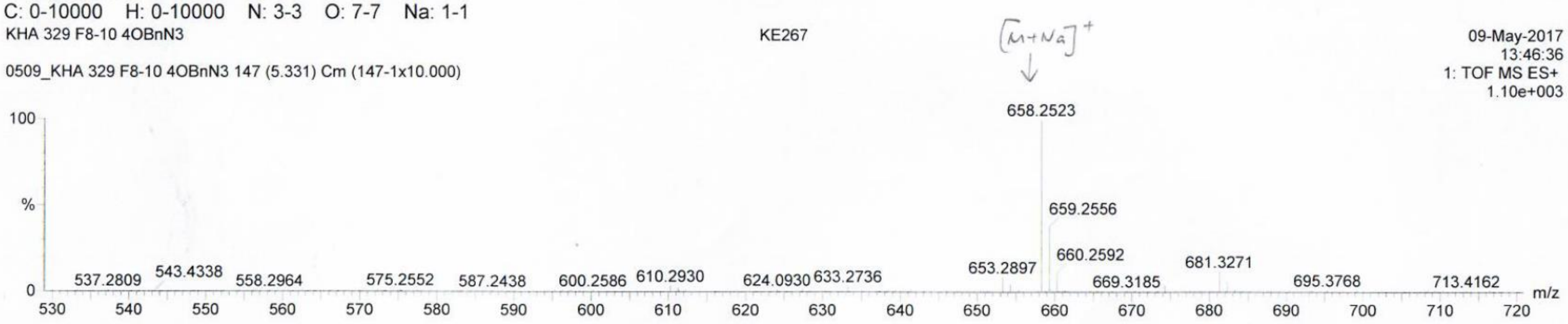

Minimum:				-1000.0			
Maximum:		5.0	5.0	1000.0			
Mass	Calc. Mass	mDa	PPM	DBE	i-FIT	i-FIT (Norm)	Formula
658.2523	658.2529	-0.6	-0.9	20.5	19.6	0.0	C37 H37 N3 O7 Na

The HRMS spectrum of compound **25**.

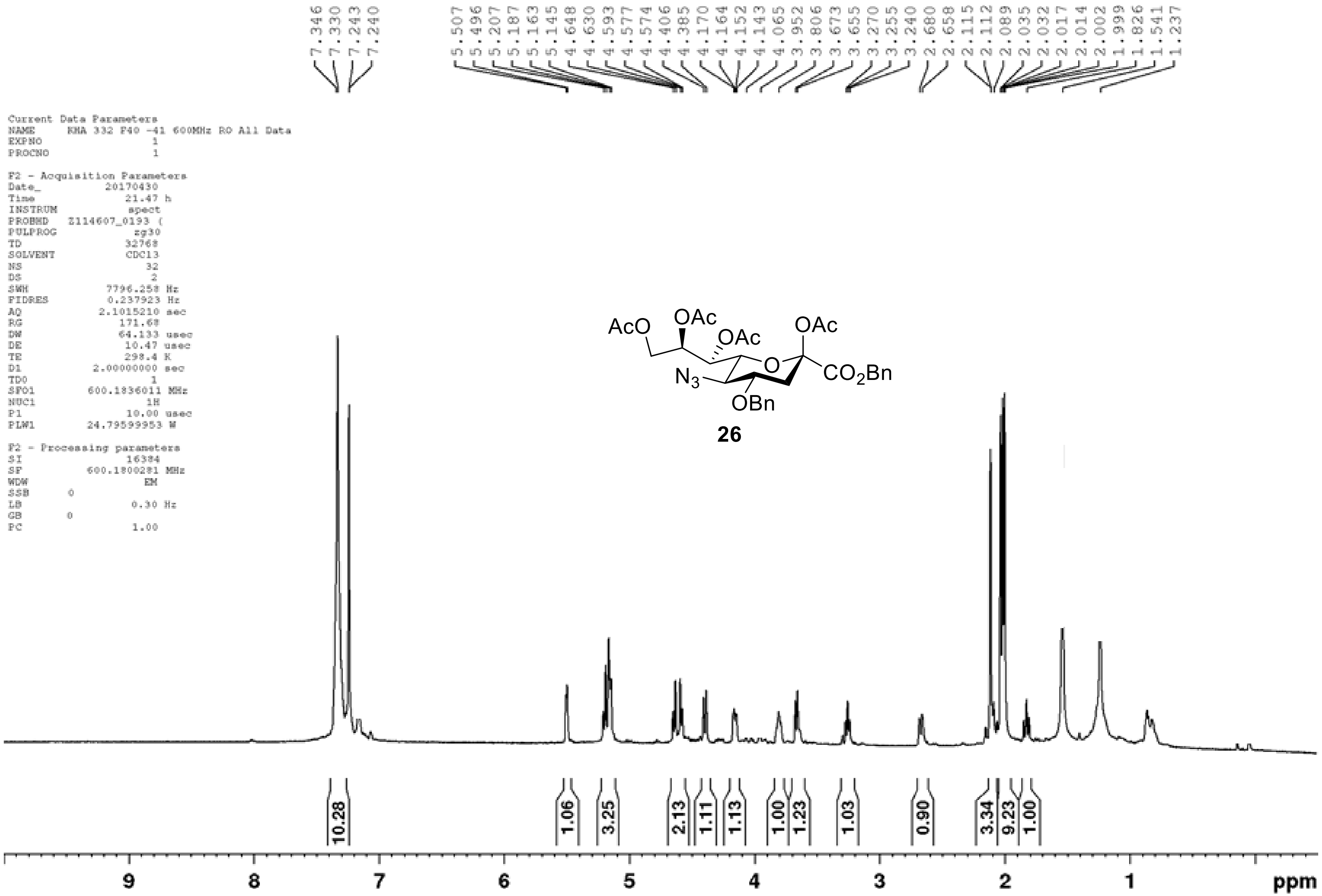

The ^{1}H NMR spectrum of compound **26**.

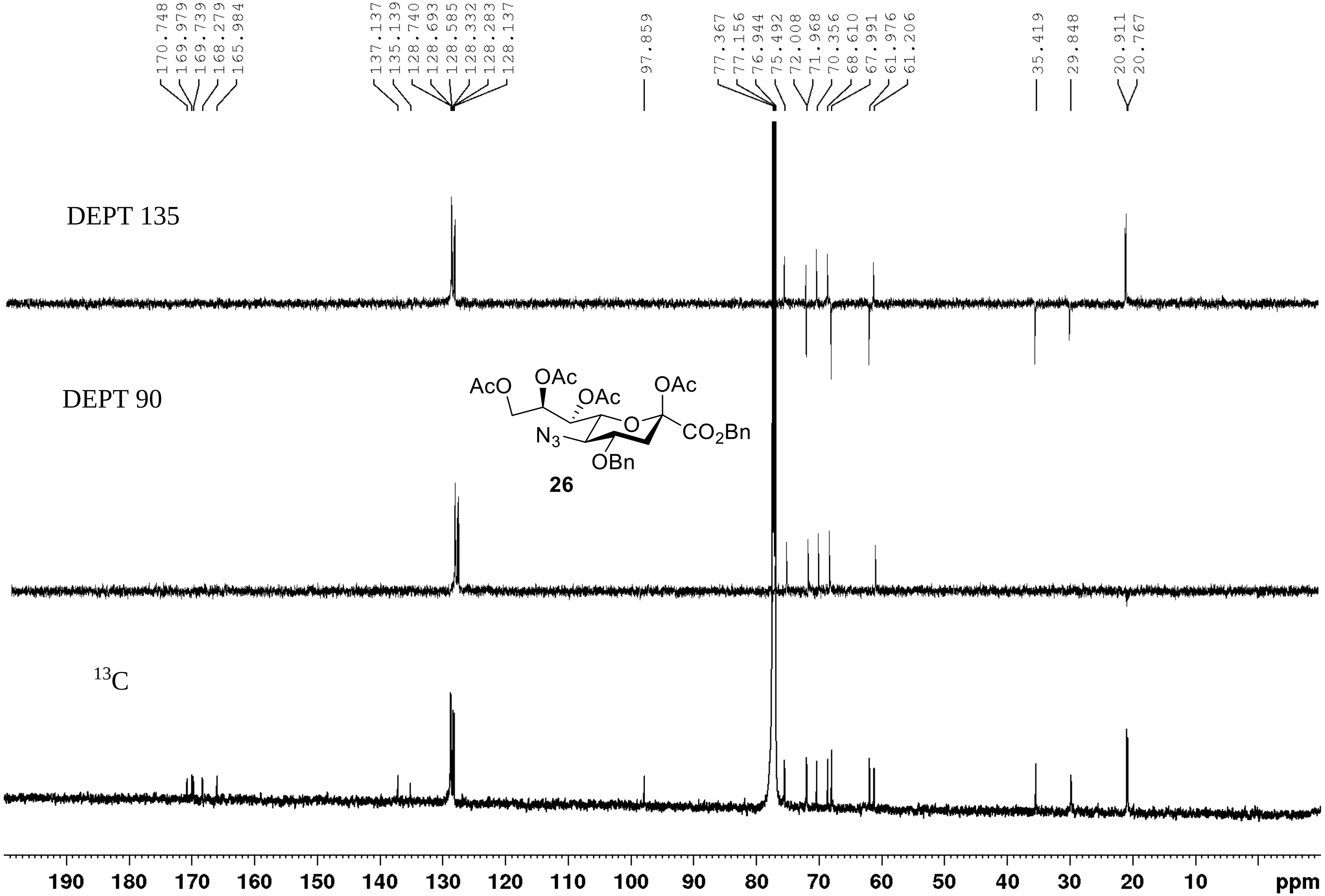

The ^{13}C NMR spectrum of compound **26**.

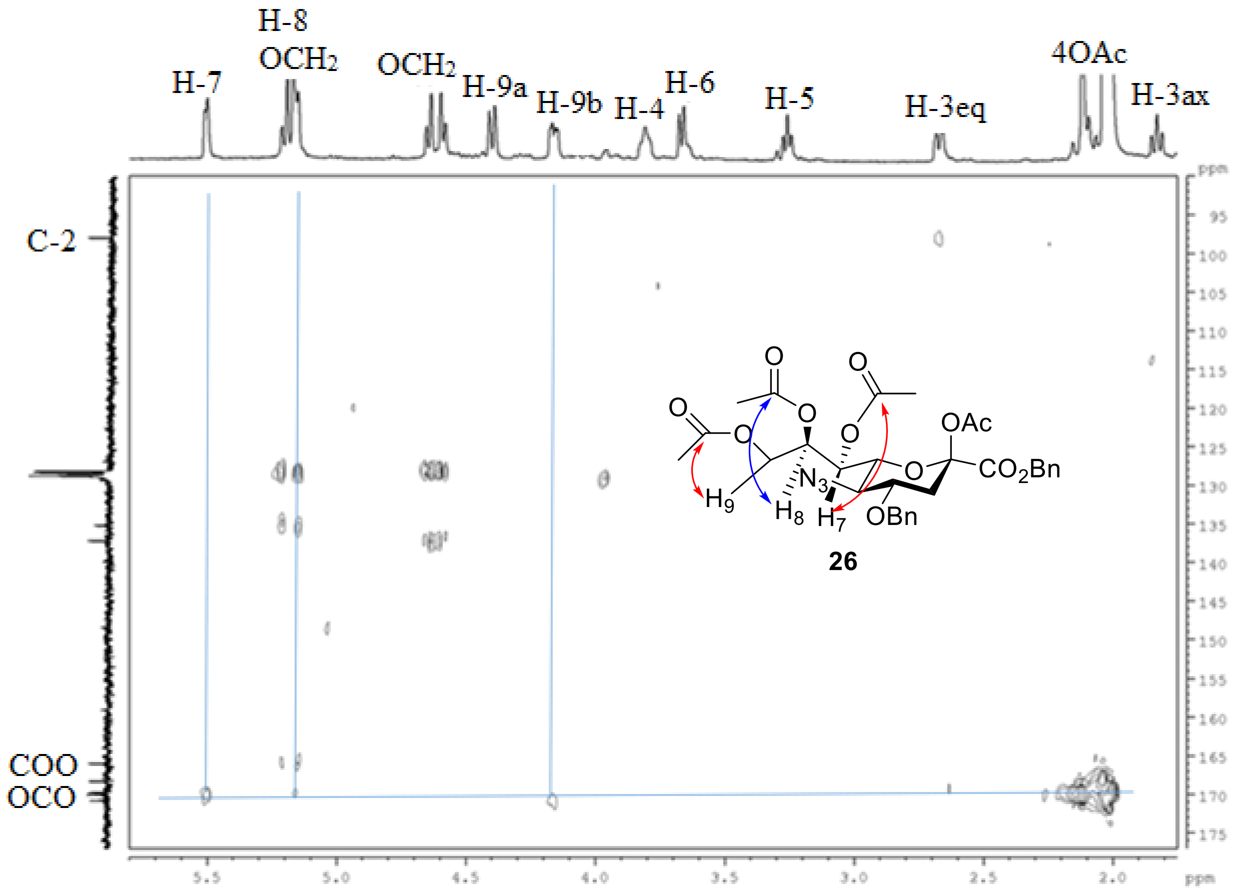

The HMBC NMR spectrum of compound **26**.

Elemental Composition Report Page 1

Single Mass Analysis
Tolerance = 20.0 PPM / DBE: min = -1000.0, max = 1000.0
Element prediction: Off
Number of isotope peaks used for i-FIT = 2

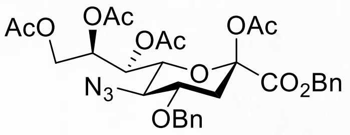

26

Monoisotopic Mass, Even Electron Ions
35 formula(e) evaluated with 1 results within limits (all results (up to 1000)) for each mass)
Elements Used:
C: 0-1000 H: 0-1000 N: 3-3 O: 12-12 Na: 1-1

KHA 332 F40-41 4OBn2OAcN3 KE267 29-Jun-2017 17:06:36

0629_KHA 332 F40-41 4OBn2OAcN3 46 (1.671) Cm (46-1x5.000) 1: TOF MS ES+ 6.95e+004

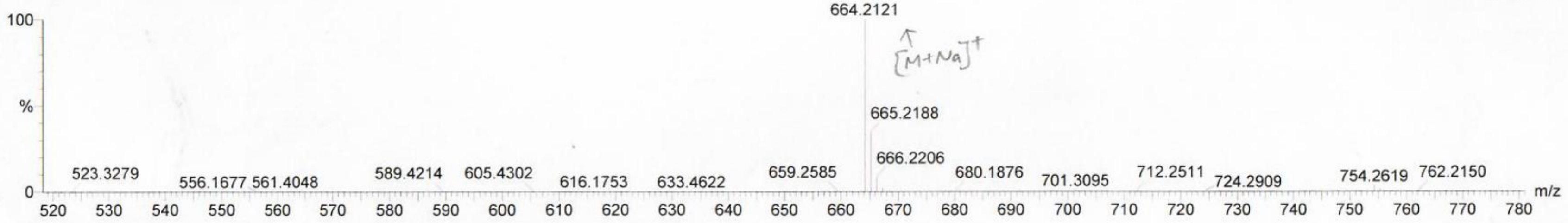

Minimum:				-1000.0			
Maximum:		5.0	20.0	1000.0			
Mass	Calc. Mass	mDa	PPM	DBE	i-FIT	i-FIT (Norm)	Formula
664.2121	664.2118	0.3	0.5	15.5	34.3	0.0	C31 H35 N3 O12 Na

The HRMS spectrum of compound **26**.

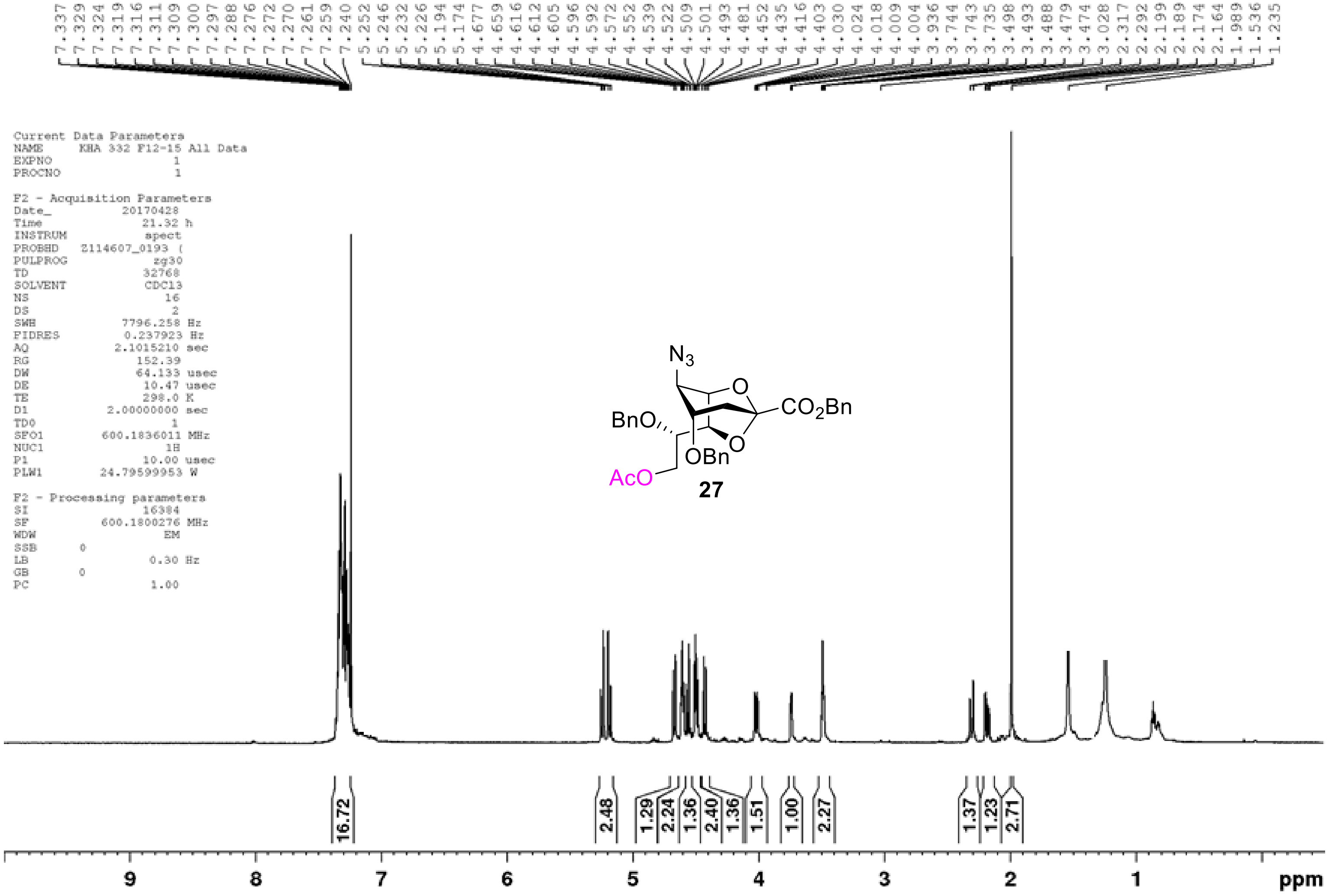

The ^1H NMR spectrum of compound **27**.

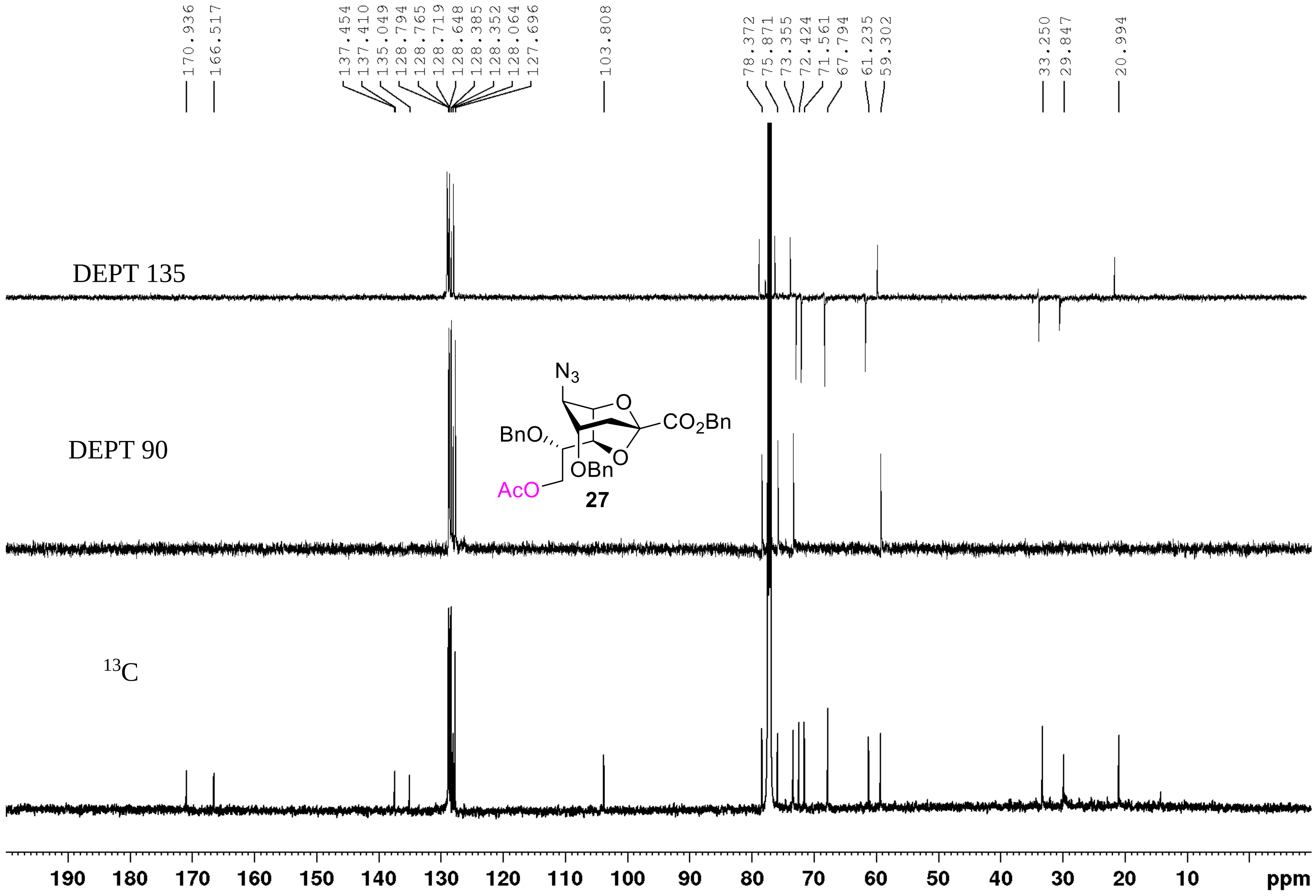

The ^{13}C NMR spectrum of compound **27**.

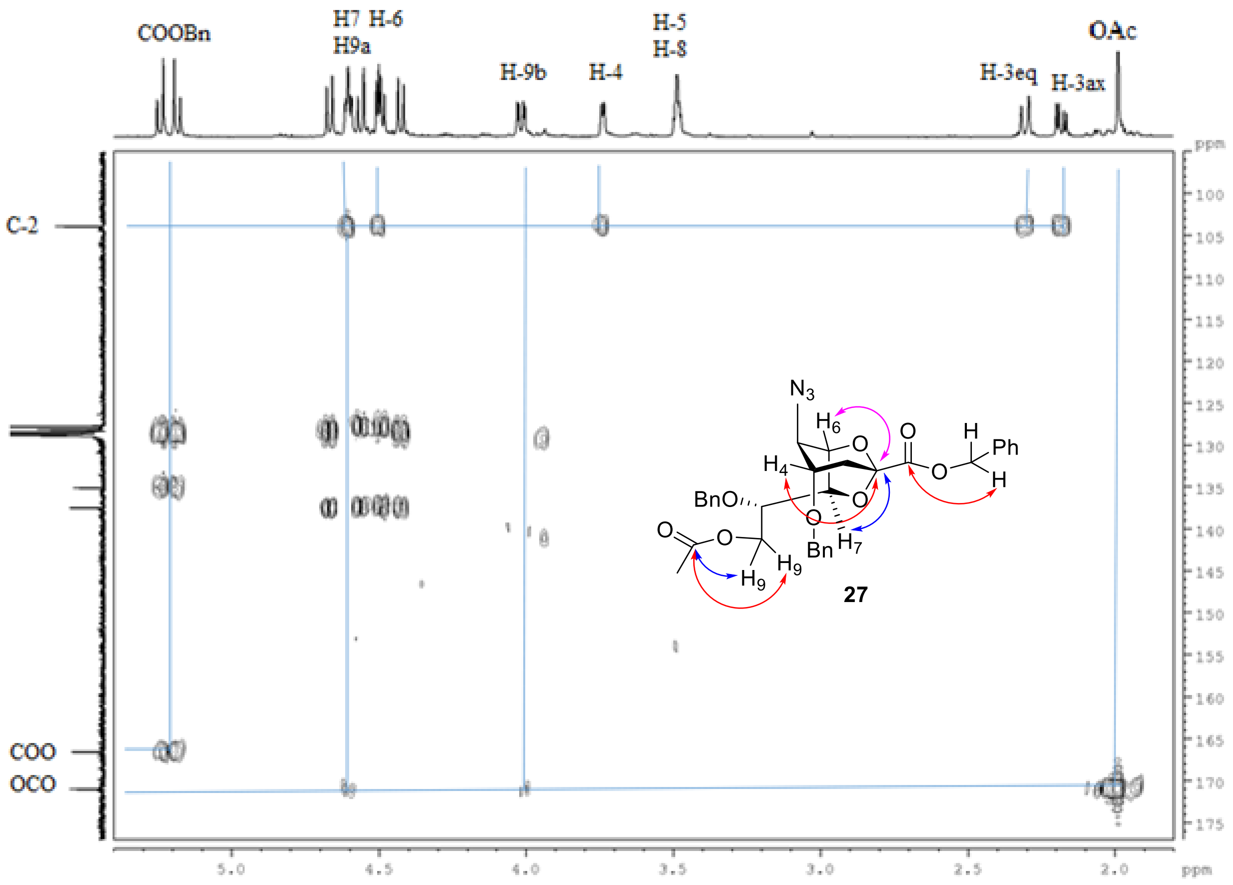

The HMBC NMR spectrum of compound **27**.

Elemental Composition Report **Page 1**

Single Mass Analysis
Tolerance = 50.0 PPM / DBE: min = -1000.0, max = 1000.0
Element prediction: Off
Number of isotope peaks used for i-FIT = 2

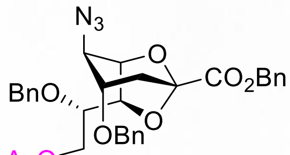

27

Monoisotopic Mass, Even Electron Ions
35 formula(e) evaluated with 1 results within limits (all results (up to 1000) for each mass)
Elements Used:
C: 0-10000 H: 0-10000 N: 3-3 O: 8-8 Na: 1-1
KHA 332 F12-15 3OBn1OAcN3 KE267 09-May-2017
13:54:38
0509_KHA 332 F12-15 3OBn1OAcN3 44 (1.582) Cm (44-1x10.000) 1: TOF MS ES+
6.72e+002

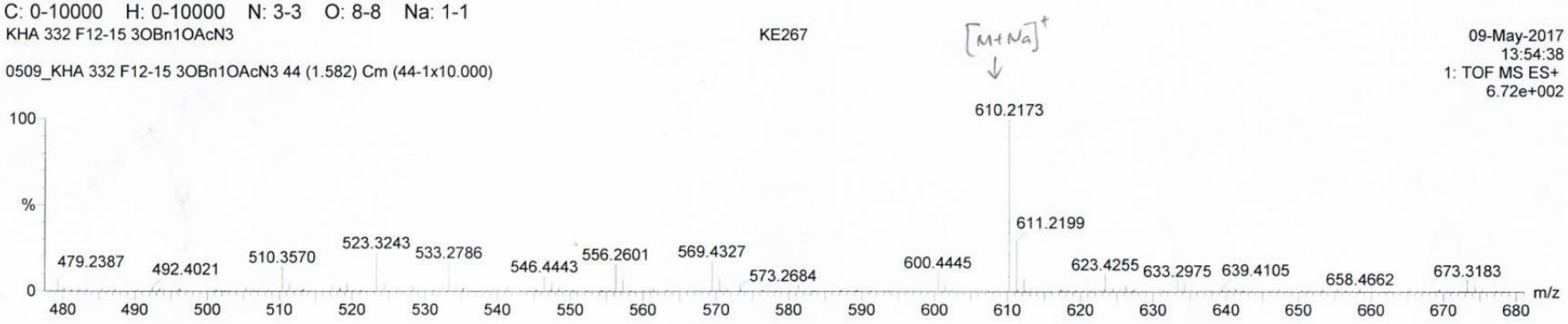

	Mass	Calc. Mass	mDa	PPM	DBE	i-FIT	i-FIT (Norm)	Formula
Minimum:					-1000.0			
Maximum:			5.0	50.0	1000.0			
	610.2173	610.2165	0.8	1.3	17.5	25.4	0.0	C32 H33 N3 O8 Na

The HRMS spectrum of compound **27**.

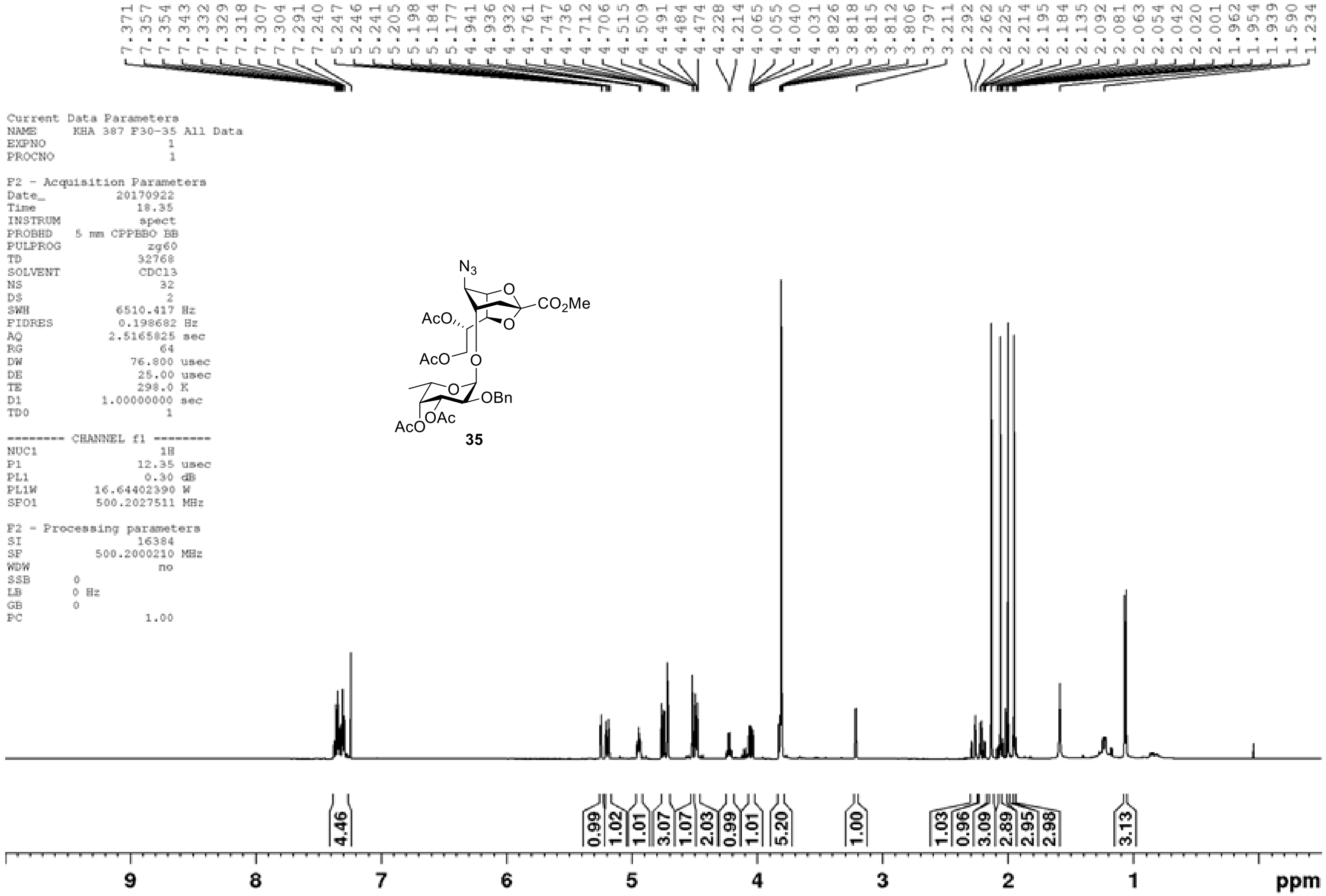

The ^1H NMR spectrum of compound **35**.

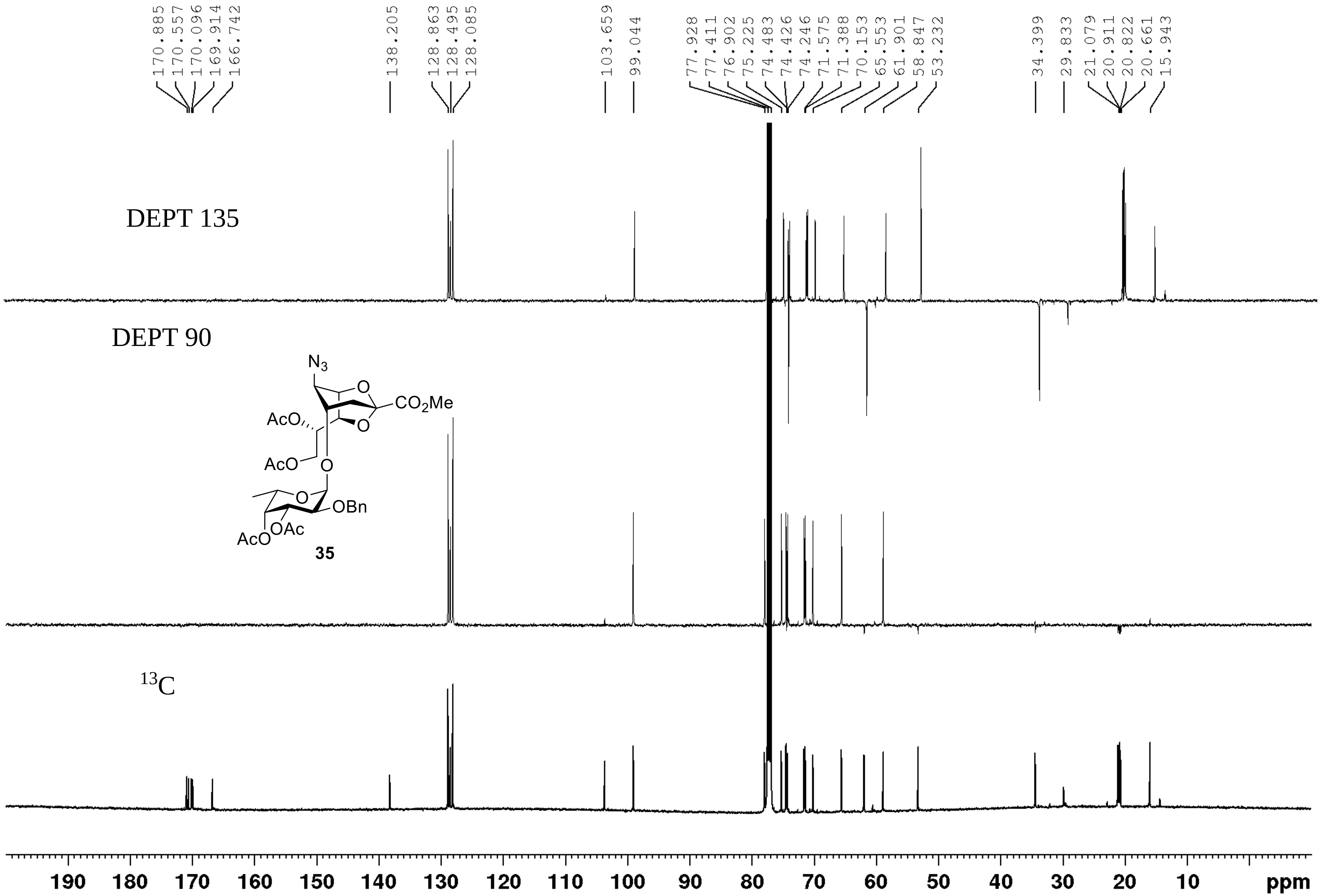

The ^{13}C NMR spectrum of compound **35**.

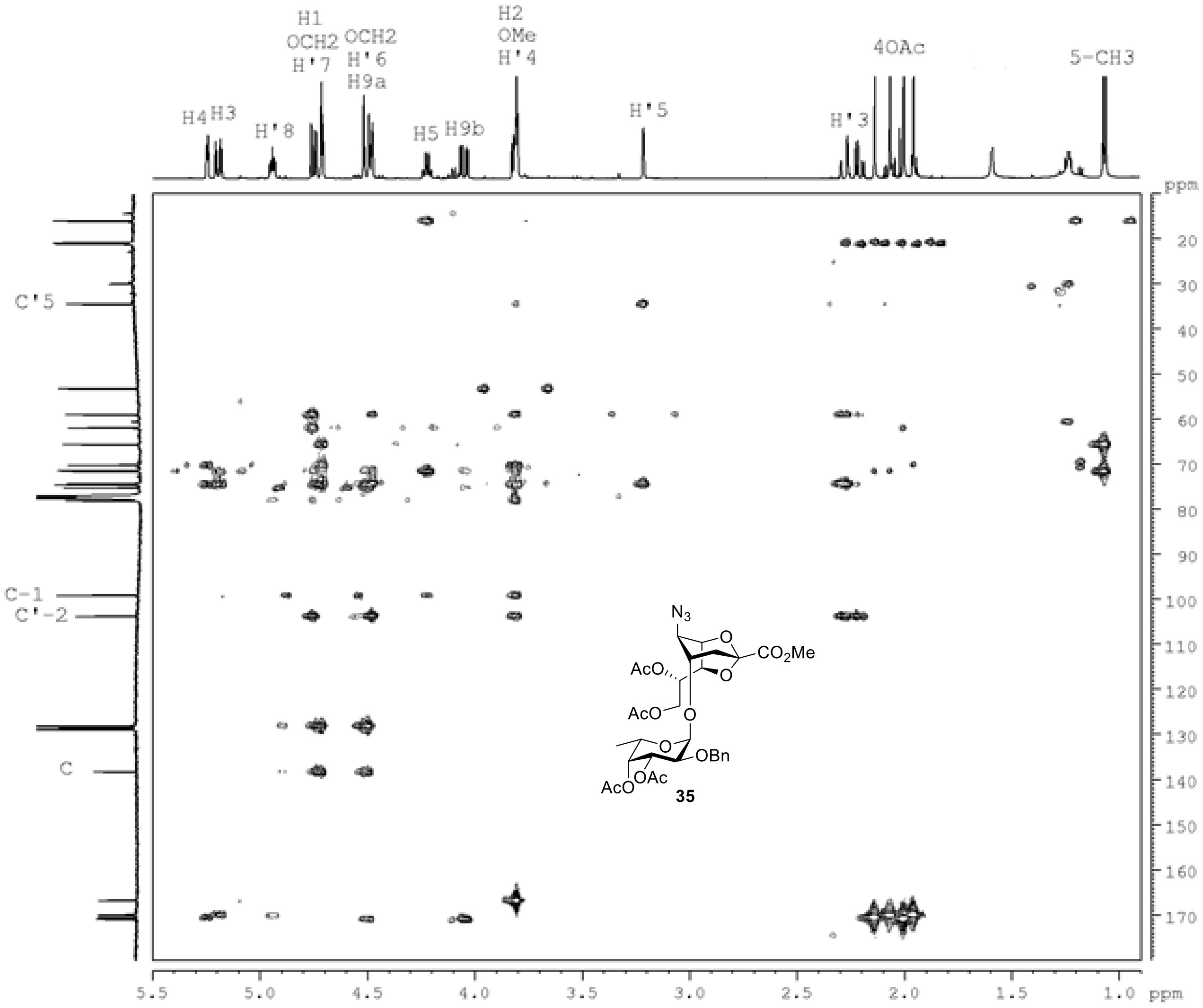

The HMBC NMR spectrum of compound **35**.

Elemental Composition Report

Page 1

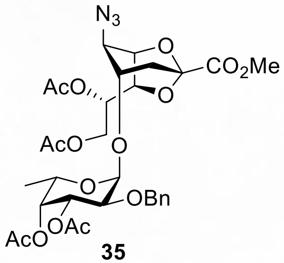

35

Single Mass Analysis

Tolerance = 40.0 PPM / DBE: min = -1000.0, max = 1000.0
Element prediction: Off
Number of isotope peaks used for i-FIT = 2

Monoisotopic Mass, Even Electron Ions
35 formula(e) evaluated with 1 results within limits (all results (up to 1000) for each mass)
Elements Used:
C: 0-400 H: 0-1000 N: 3-3 O: 15-15 Na: 1-1

KHA 387 F30-35 N3 FucoDisac KE267 27-Sep-2017 16:57:28

0927_KHA 387 F30-35 N3 FucoDisac 27 (0.970) Cm (27-1x10.000) 1: TOF MS ES+ 5.46e+003

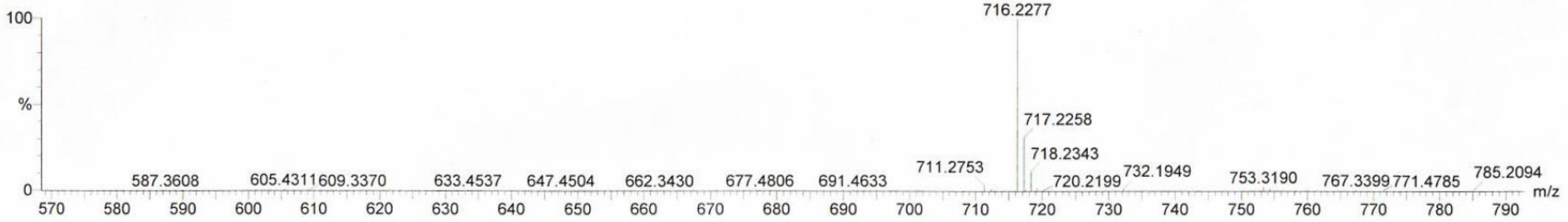

	Mass	Calc. Mass	mDa	PPM	DBE	i-FIT	i-FIT (Norm)	Formula
Minimum:					-1000.0			
Maximum:			5.0	40.0	1000.0			
	716.2277	716.2279	-0.2	-0.3	13.5	22.7	0.0	C31 H39 N3 O15 Na

The HRMS spectrum of compound **35**.

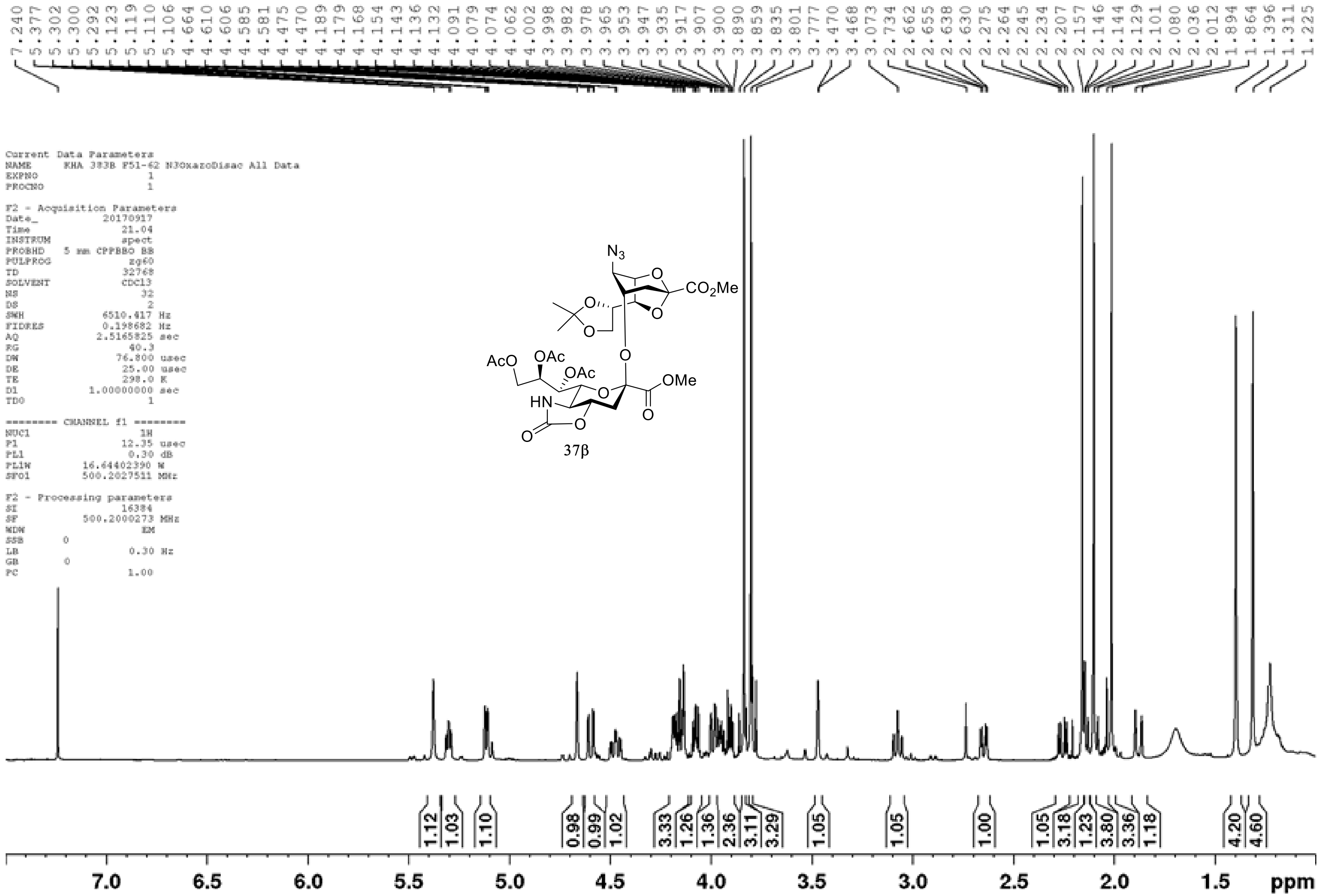

The ^{1}H NMR spectrum of compound **37β**.

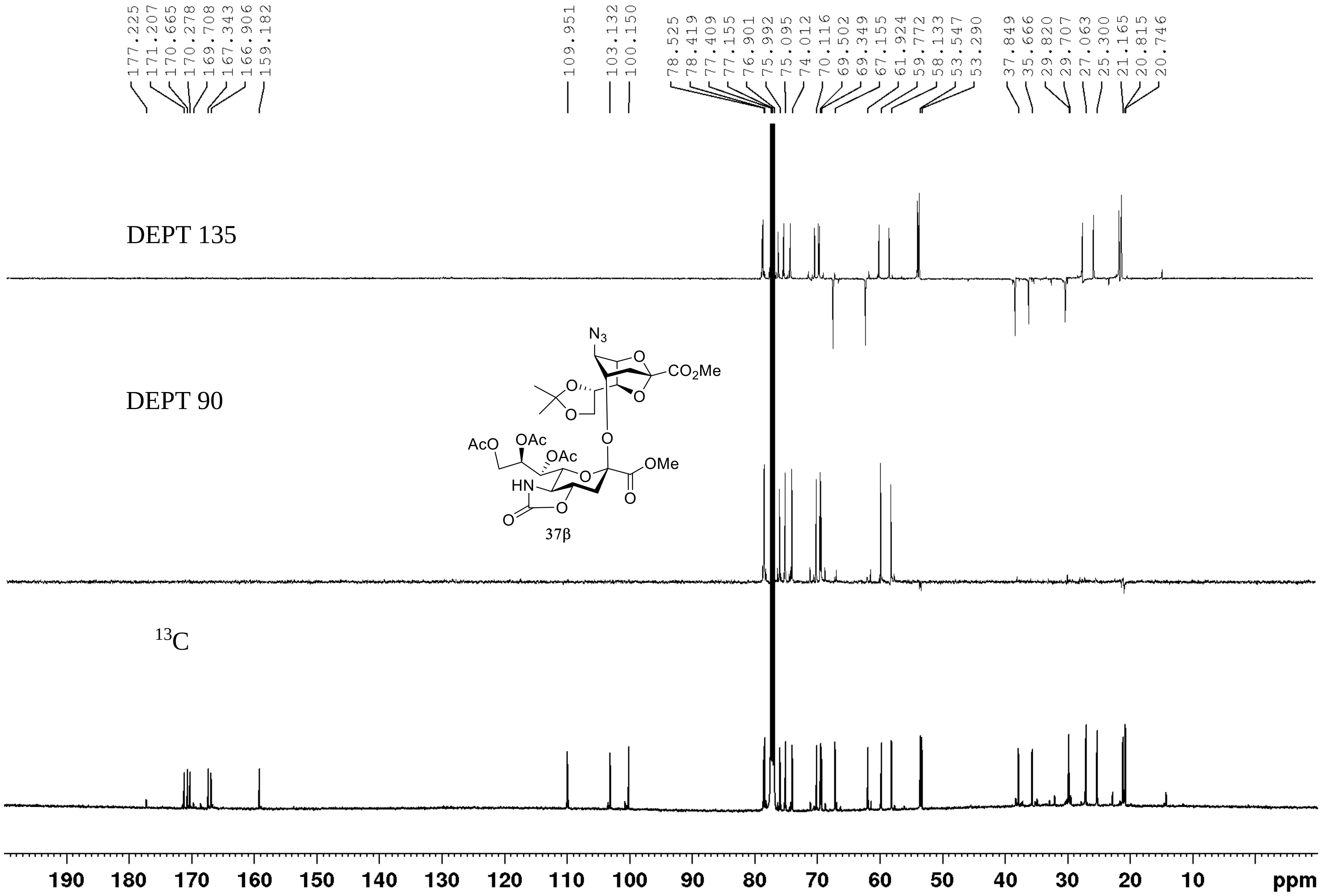

The ^{13}C NMR spectrum of compound **37β**.

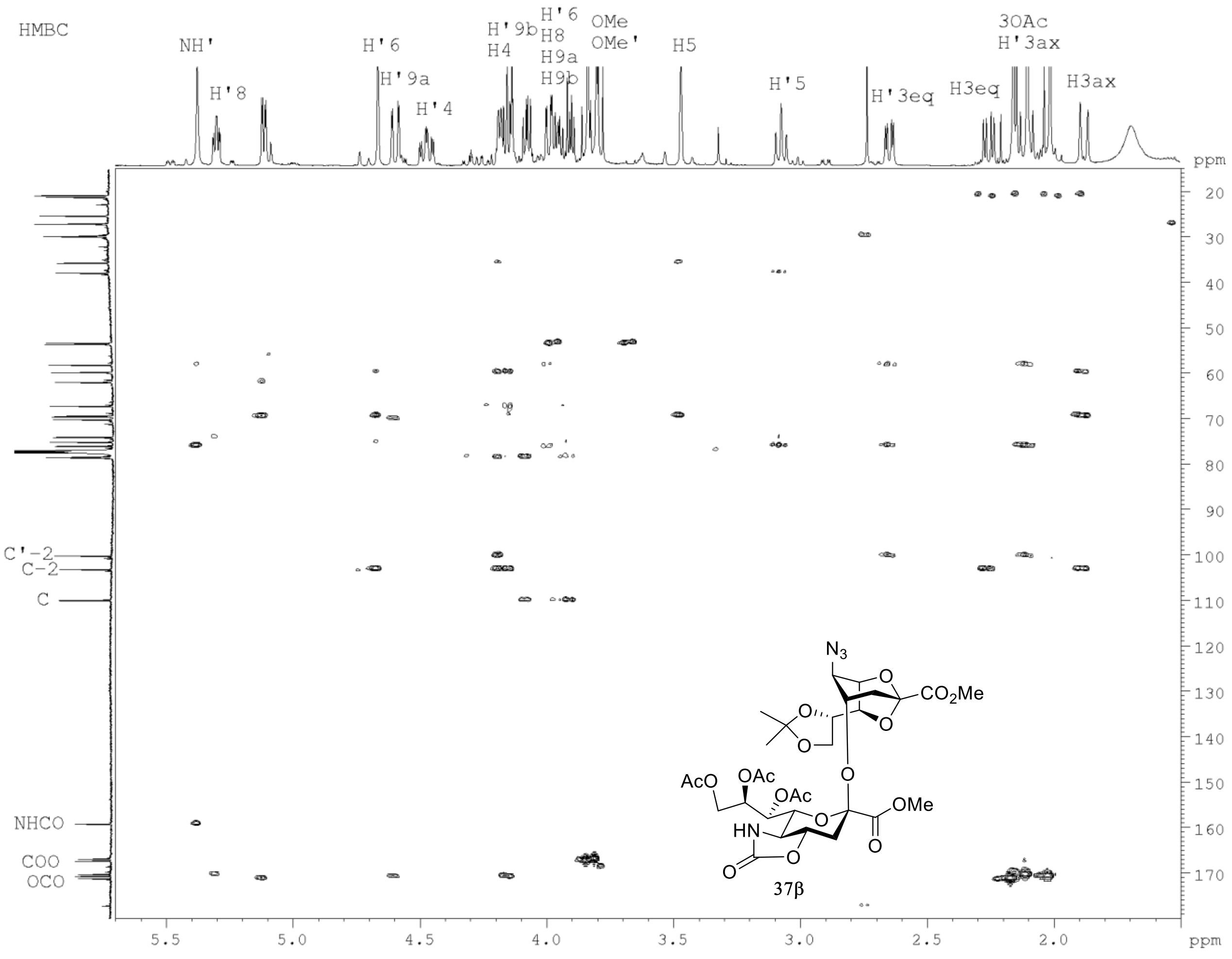

The HMBC NMR spectrum of compound **37β**.

Elemental Composition Report

Page 1

Single Mass Analysis
Tolerance = 20.0 PPM / DBE: min = -1000.0, max = 1000.0
Element prediction: Off
Number of isotope peaks used for i-FIT = 2

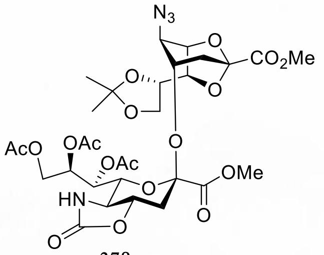

37β

Monoisotopic Mass, Even Electron Ions
34 formula(e) evaluated with 1 results within limits (all results (up to 1000) for each mass)
Elements Used:
C: 0-400 H: 0-1000 N: 4-4 O: 18-18 Na: 1-1
KHA 383 F44-52 N3NHDisac

KE267

05-Sep-2017
16:44:40
1: TOF MS ES+
4.15e+003

0905_KHA 383 F44-52 N3NHDisac 17 (0.629) Cm (17-6x10.000)

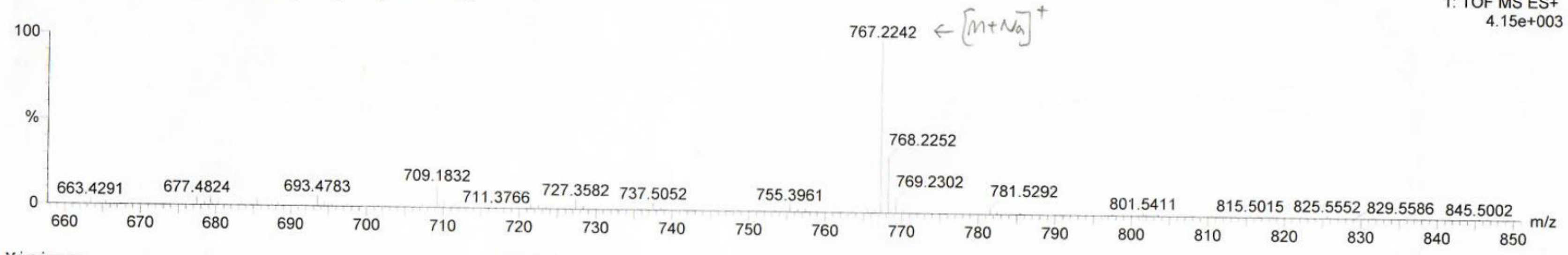

Minimum:				-1000.0			
Maximum:		5.0	20.0	1000.0			
Mass	Calc. Mass	mDa	PPM	DBE	i-FIT	i-FIT (Norm)	Formula
767.2242	767.2235	0.7	0.9	12.5	27.0	0.0	C30 H40 N4 O18 Na

The HRMS spectrum of compound **37β**.

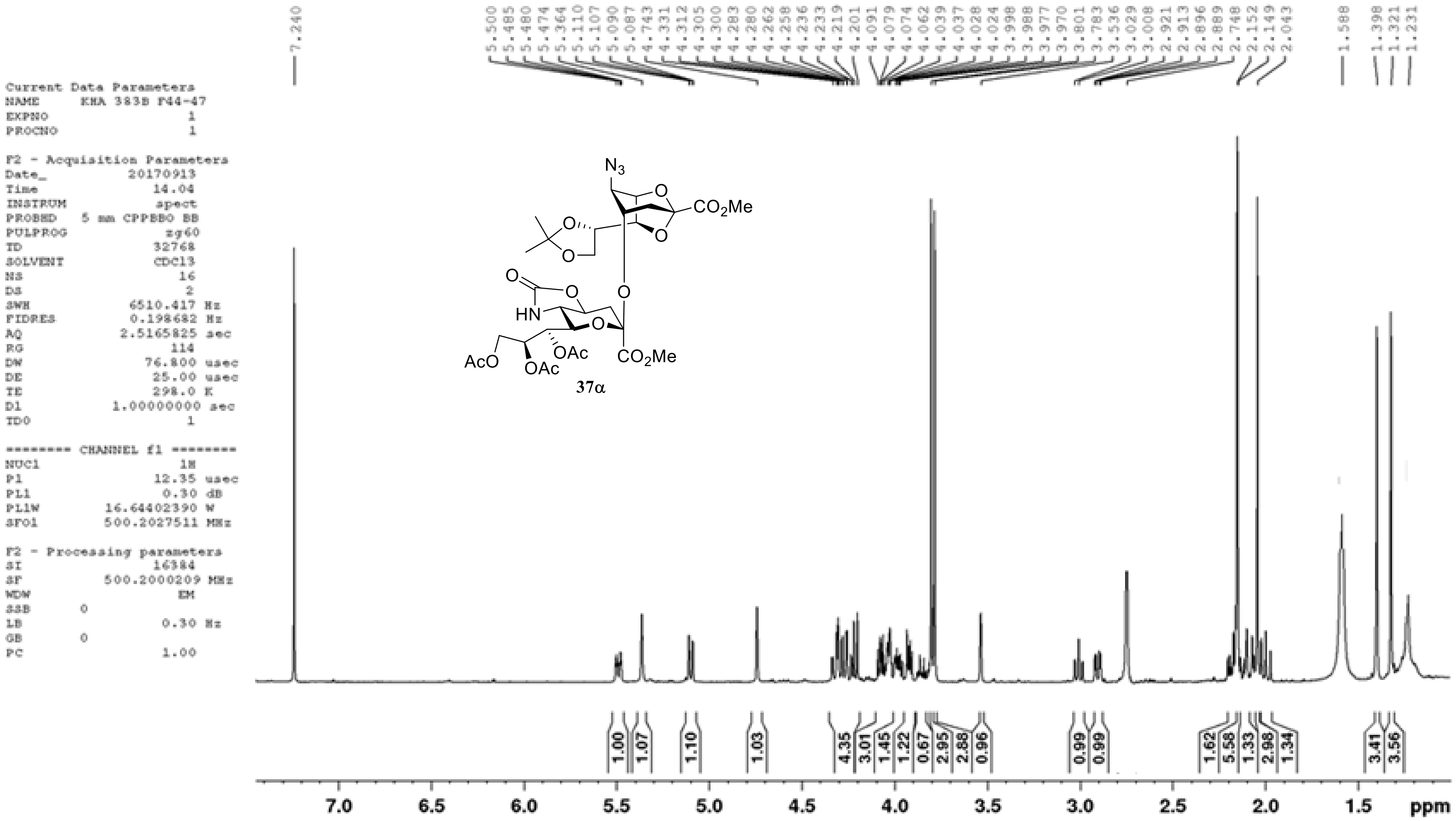

The ^1H NMR spectrum of compound **37α**.

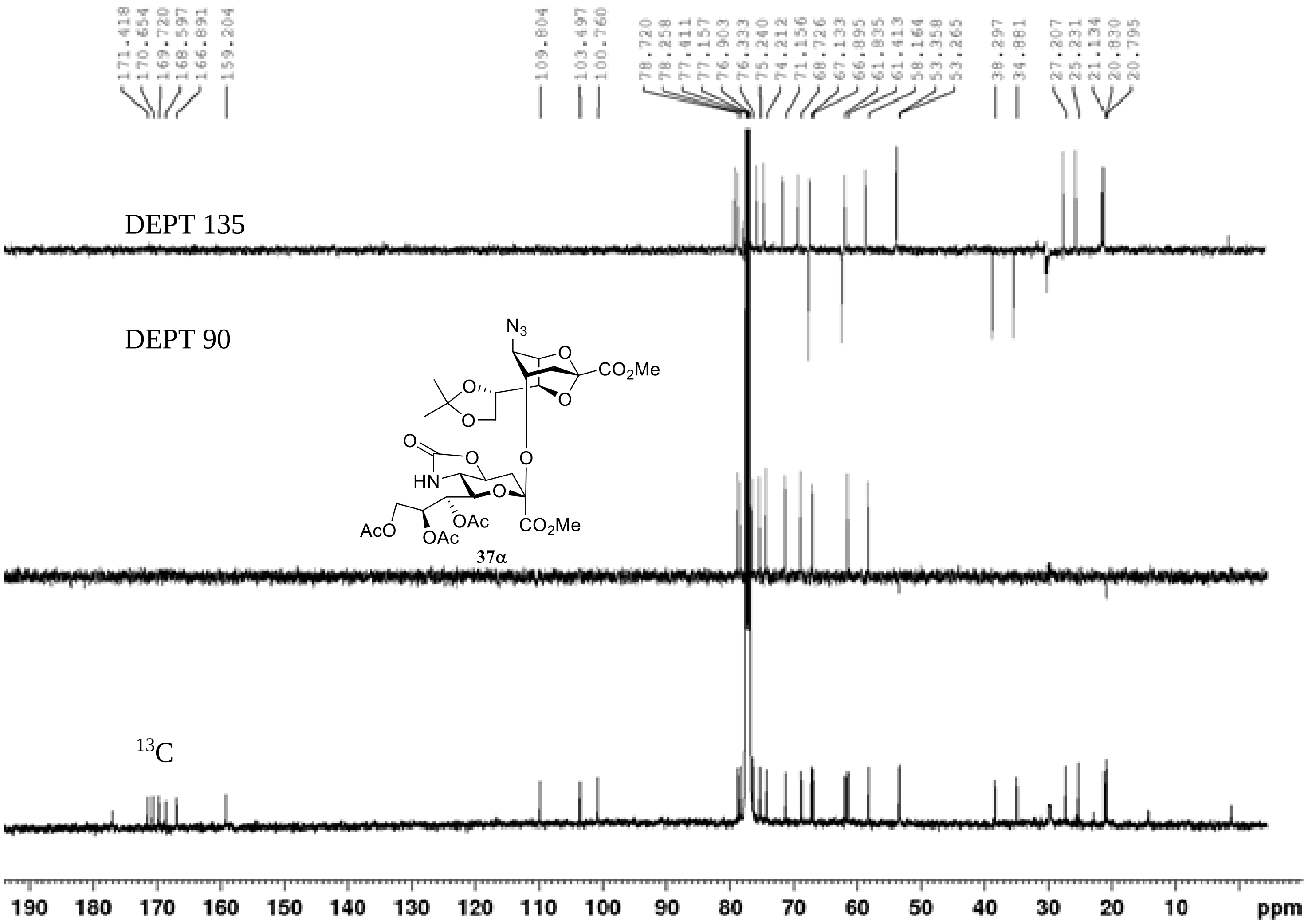

The ^{13}C NMR spectrum of compound **37α**.

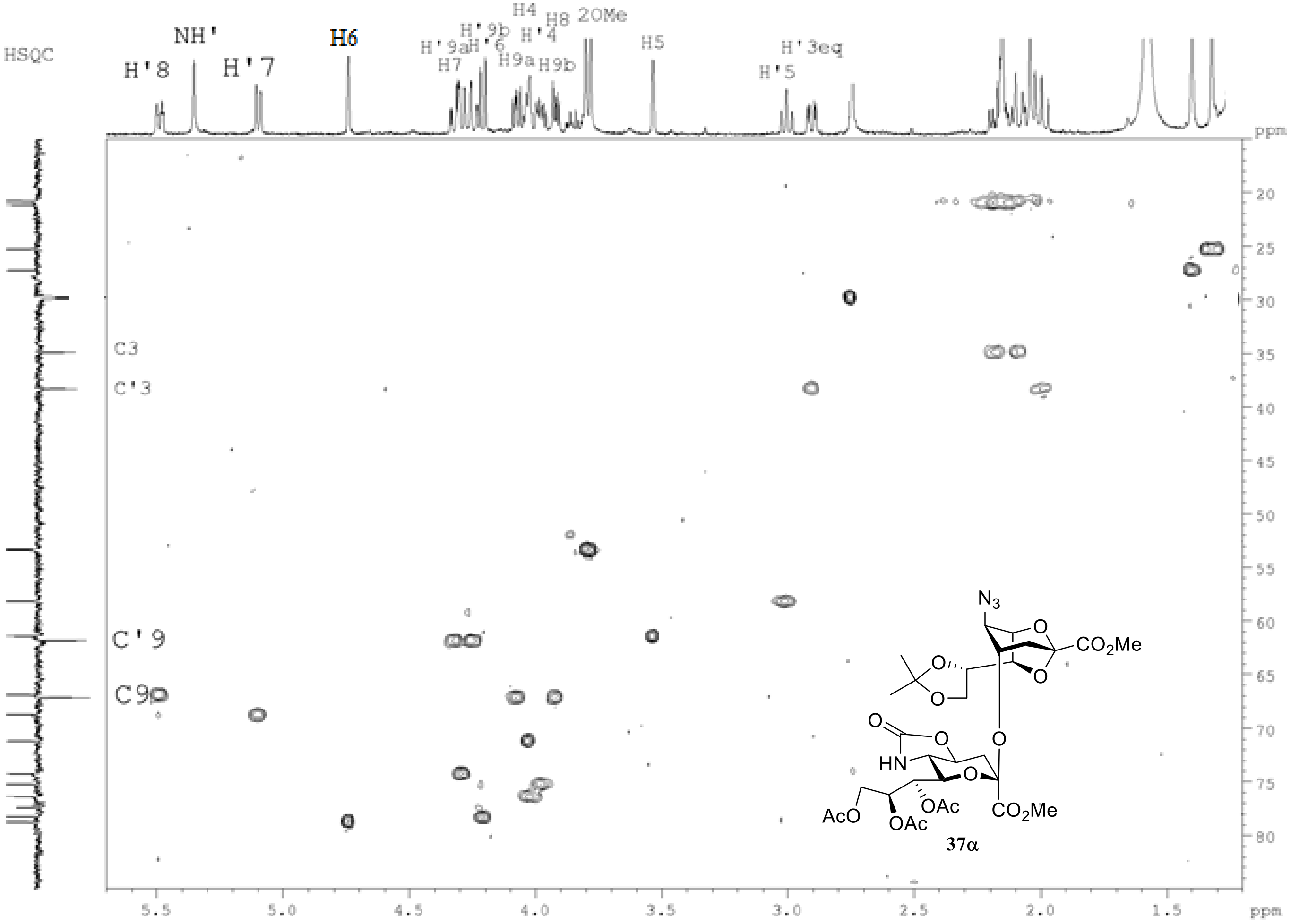

The HSQC NMR spectrum of compound **37α**.

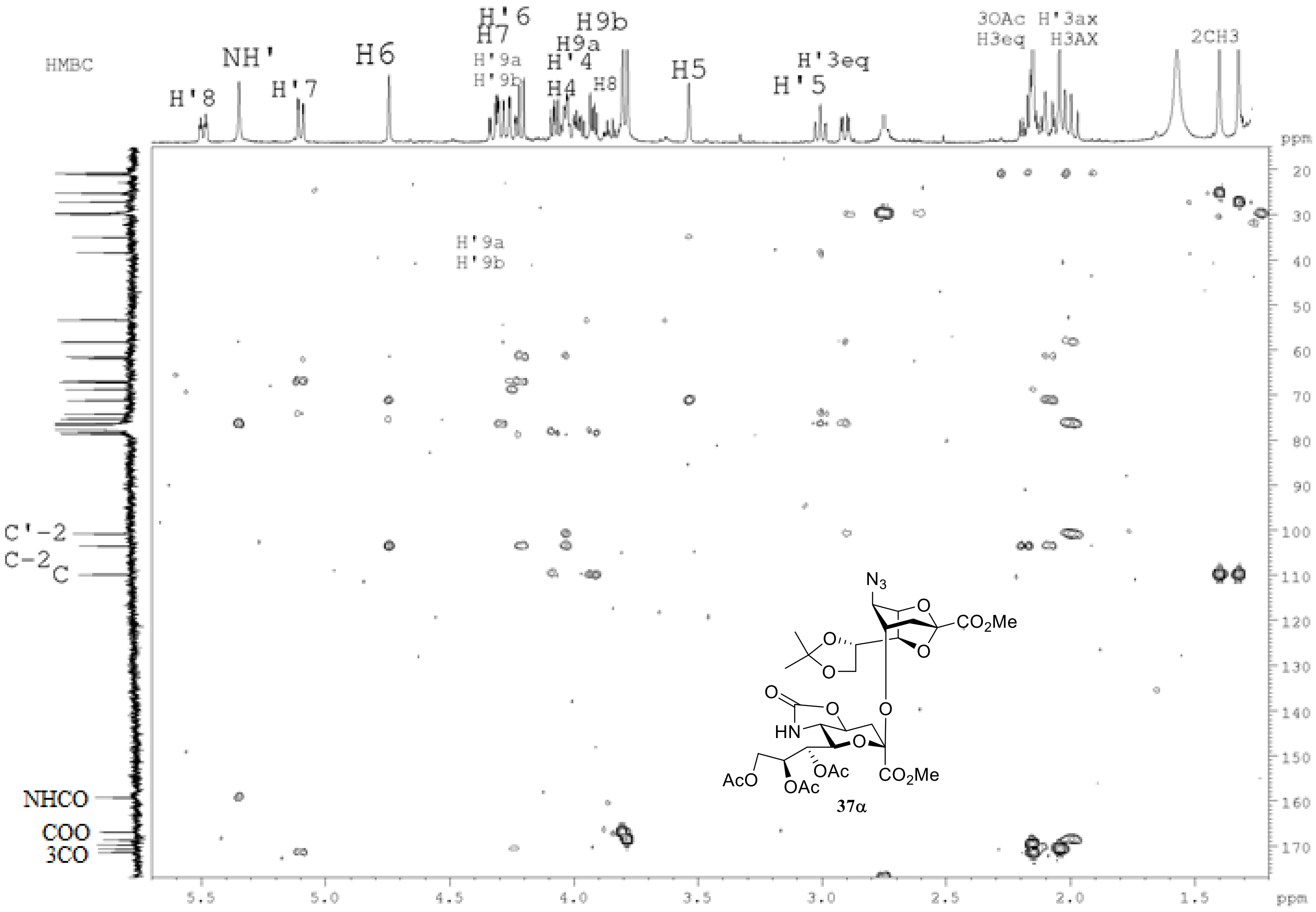

The HMBC NMR spectrum of compound **37α**.

Spectrum

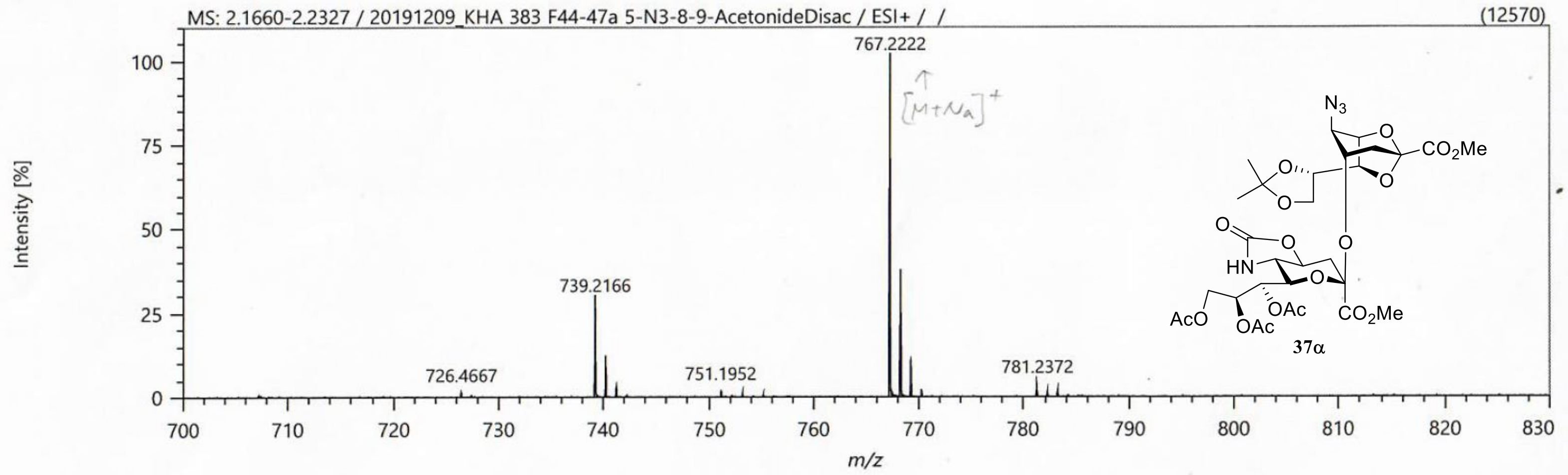

Elemental Composition

Parameters

Tolerance:	±50.00 ppm
Electron:	Odd/Even
Charge:	+1
DBE:	-1.5 - 999.0

Elements Set 1:

Symbol	C	H	O	Na	N	S	P	Si	F	Cl
Min	0	0	18	1	4	0	0	0	0	0
Max	400	1000	18	1	4	0	0	0	0	0

Results

Mass	Formula	Calculated Mass	Mass Difference [mDa]	Mass Difference [ppm]	DBE
767.22223	C30 H40 N4 O18 Na	767.22298	-0.75	-0.98	12.5

The HRMS spectrum of compound **37α**.

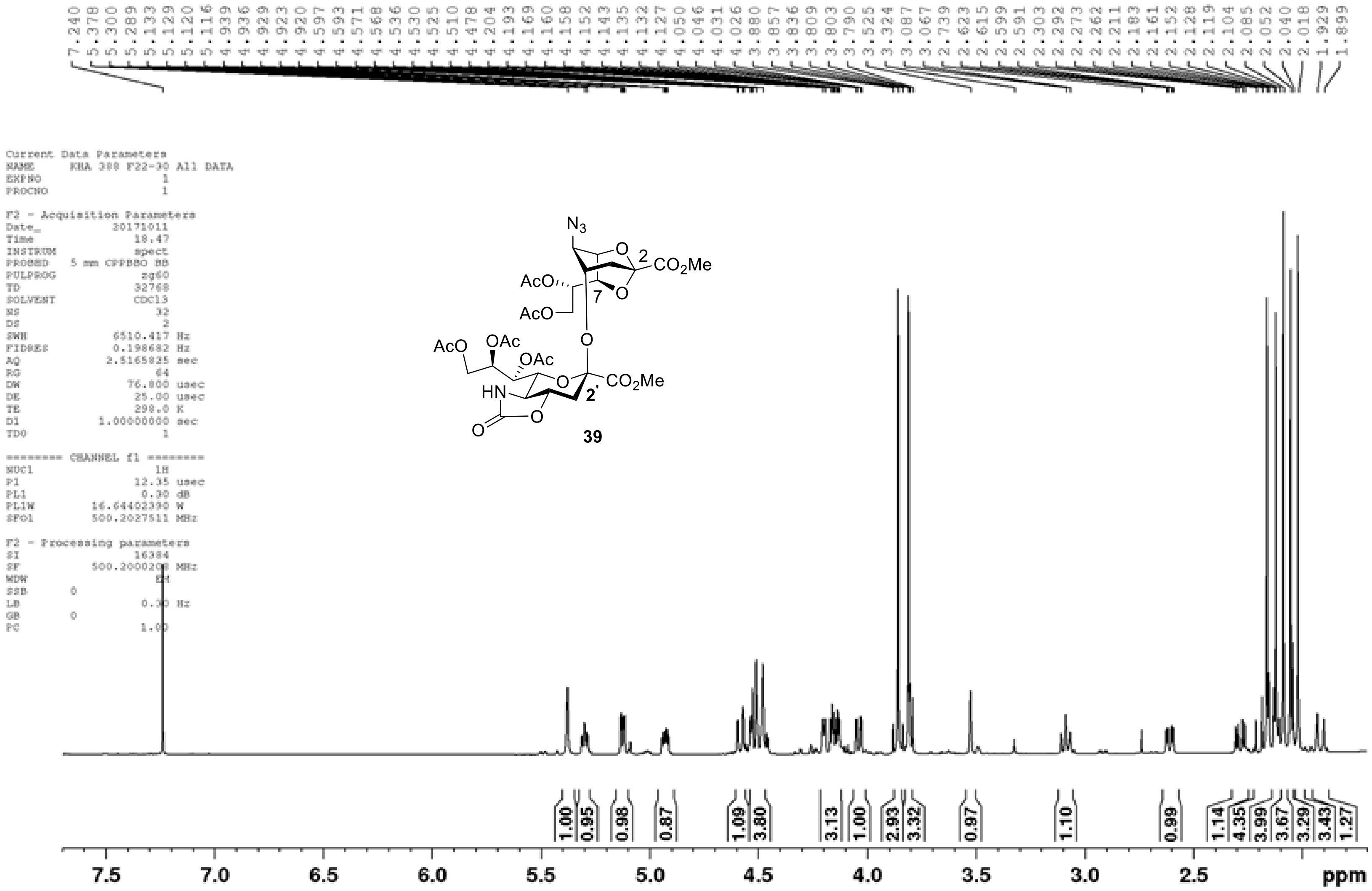

The ^{1}H NMR spectrum of compound **39**.

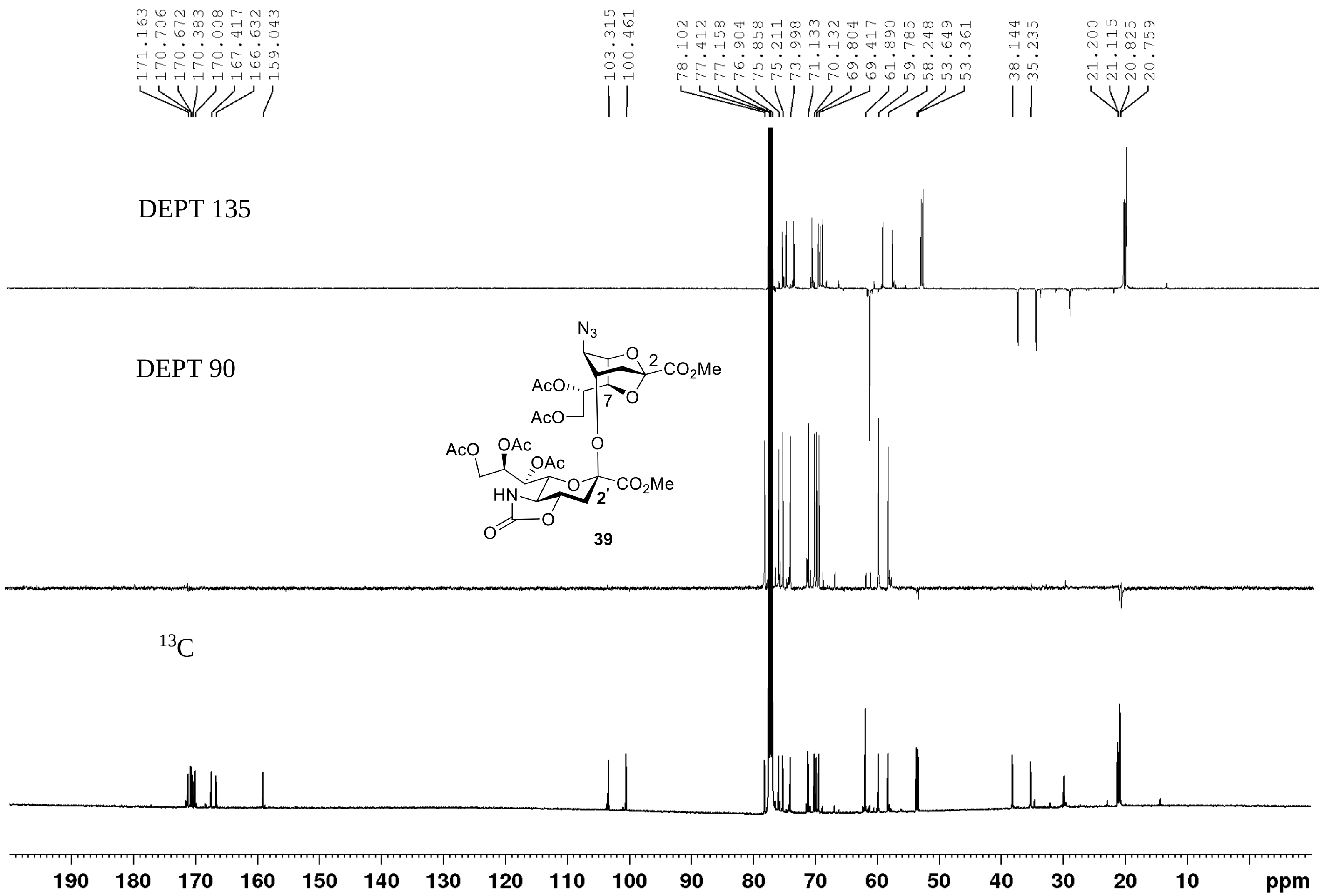

The ^{13}C NMR spectrum of compound **39**.

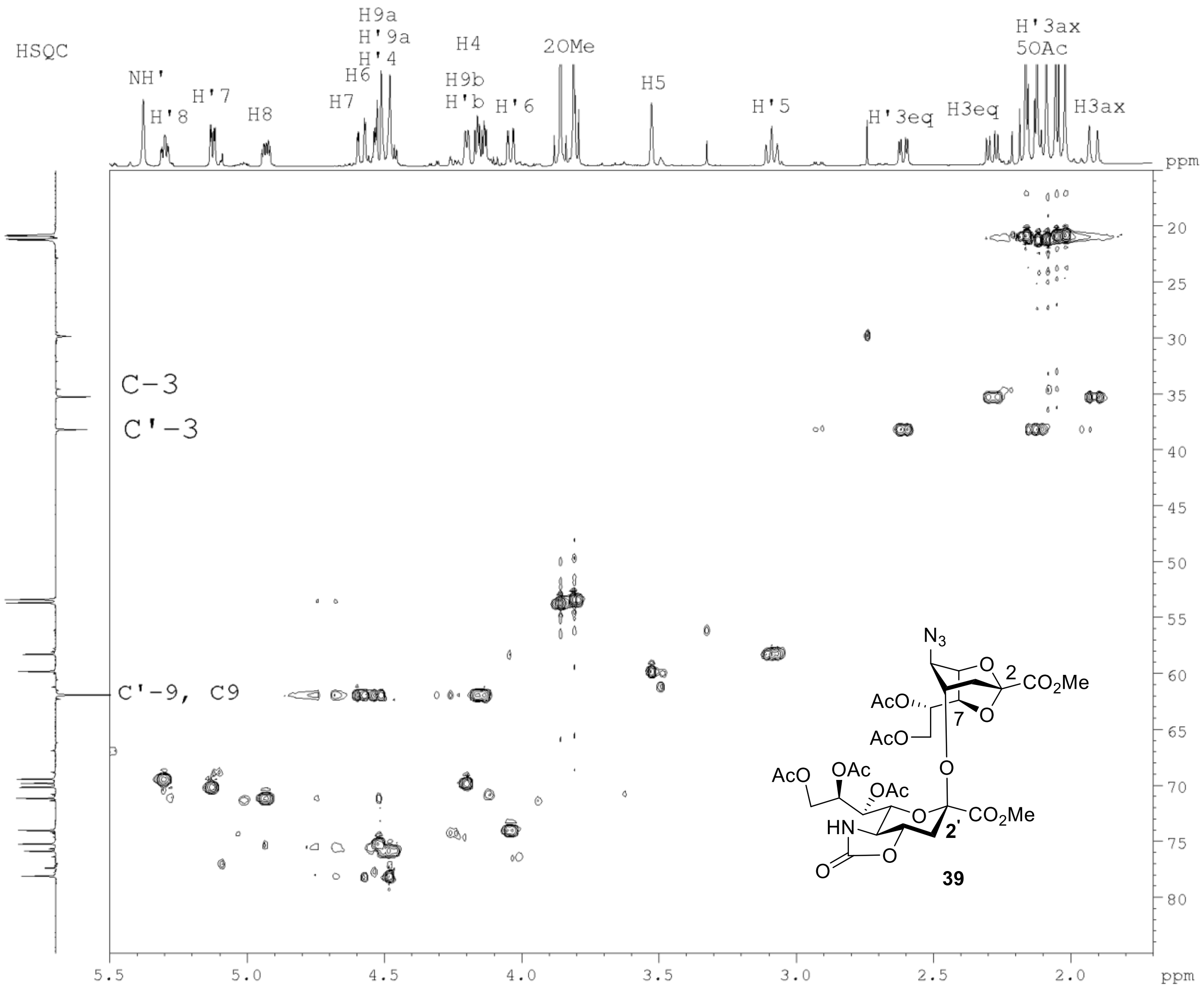

The HSQC NMR spectrum of compound **39**.

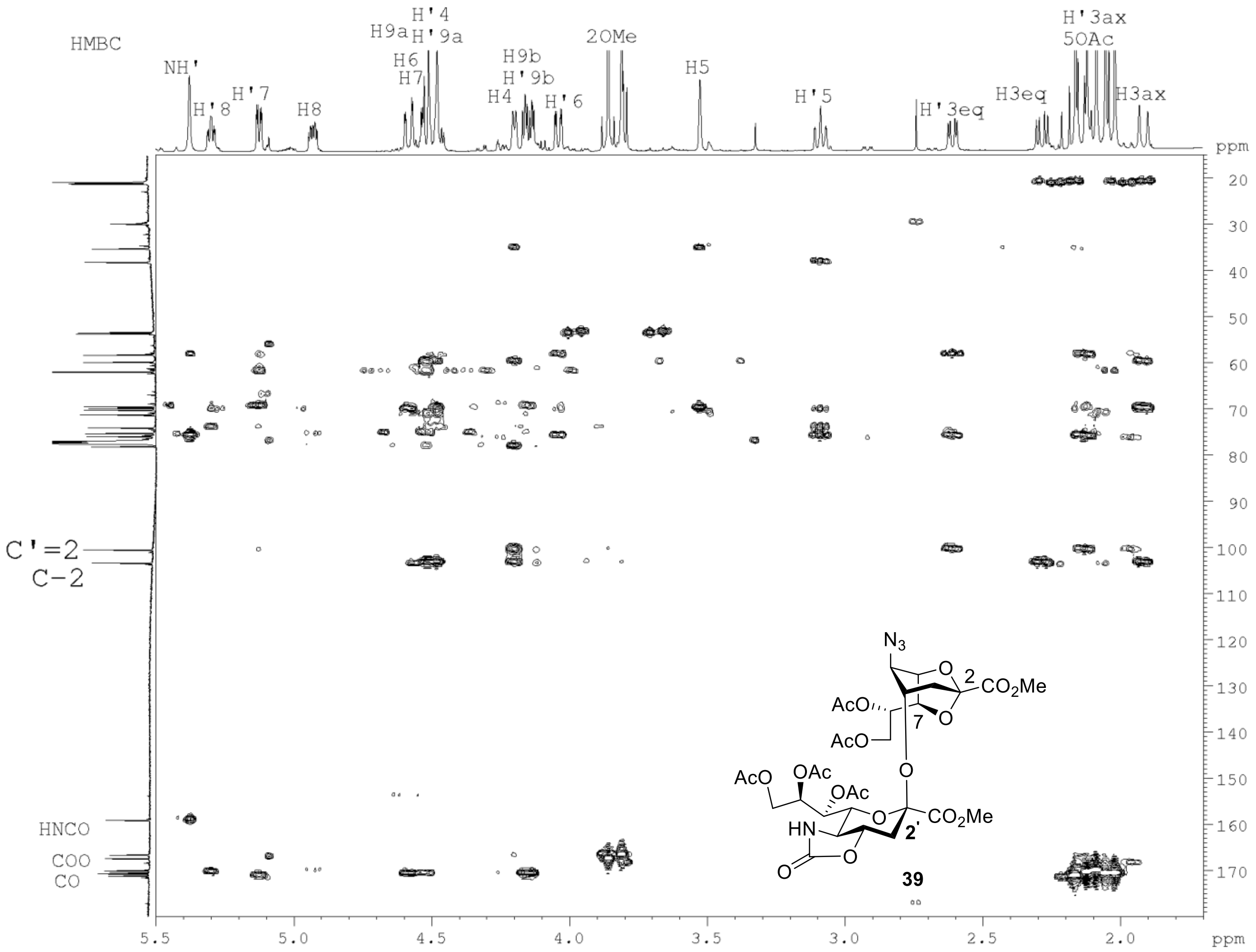

The HMBC NMR spectrum of compound **39**.

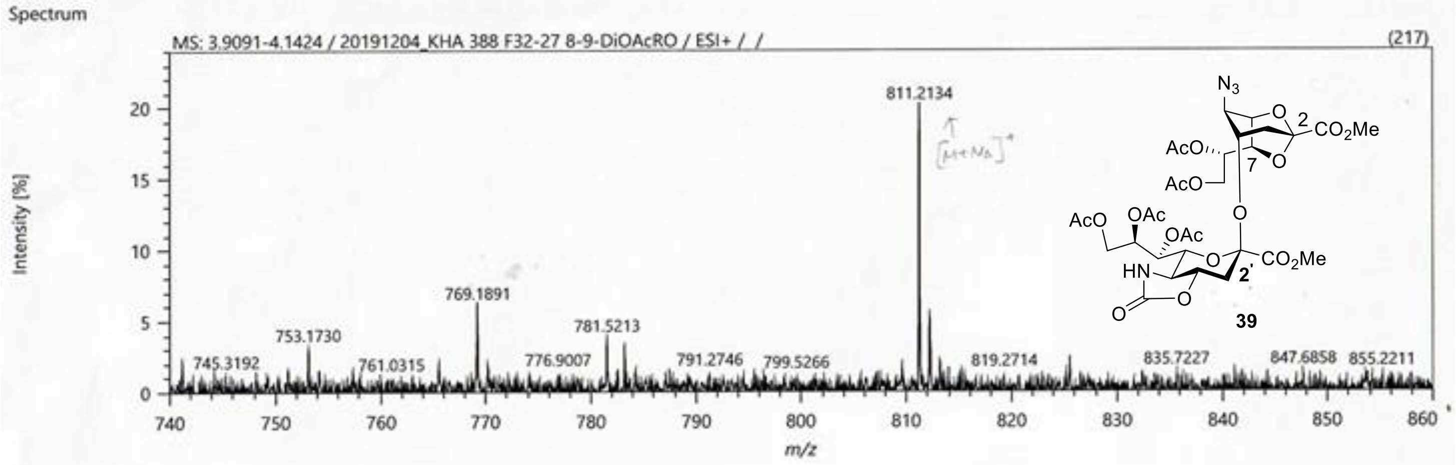

Elemental Composition

Parameters	
Tolerance:	±10.00 ppm
Electron:	Odd/Even
Charge:	+1
DBE:	-1.5 - 999.0

Elements Set 1:

Symbol	C	H	O	Na	N	S	P	Si	F	Cl
Min	0	0	20	1	4	0	0	0	0	0
Max	400	1000	20	1	4	0	0	0	0	0

Results

Mass	Formula	Calculated Mass	Mass Difference [mDa]	Mass Difference [ppm]	DBE
811.21336	C31 H40 N4 O20 Na	811.21281	0.54	0.67	13.5

The HRMS spectrum of compound **39**.

References

1. Crich, D.; Wenju, L. *J. Org. Chem.* **2007,** *72,* 2387–2391. doi:10.1021/jo062431r
