# Supplementary material for: SnCl4-catalyzed solvent-free acetolysis of 2,7-anhydrosialic acid derivatives
Source: Beilstein J Org Chem. 2019 Dec 23;15:2990–9. doi: 10.3762/bjoc.15.295 (PMC6948141; doi:10.3762/bjoc.15.295)
Supplement: File 2 — X-ray crystallographic data for compound 4. [file Beilstein_J_Org_Chem-15-2990-s002.pdf]

BEILSTEIN JOURNAL OF ORGANIC CHEMISTRY

Supporting Information

for

$SnCl_4$-catalyzed solvent-free acetolysis of 2,7-anhydrosialic acid derivatives

Kesatebrhan Haile Asressu and Cheng-Chung Wang

Beilstein J. Org. Chem. **2019,** *15,* 2990–2999. doi:10.3762/bjoc.15.295

X-ray crystallographic data for compound 4

License and Terms: This is a supporting information file under the terms of the Creative Commons Attribution License (https://creativecommons.org/licenses/by/4.0). Please note that the reuse, redistribution and reproduction in particular requires that the authors and source are credited.
The license is subject to the *Beilstein Journal of Organic Chemistry* terms and conditions: (https://www.beilstein-journals.org/bjoc)

Table S1: Crystal data and structure refinement for i16965 (**4**).

Identification code	i16965	
Empirical formula	C15 H25 N O9	
Formula weight	363.36	
Temperature	100.0(2) K	
Wavelength	0.71073 Å	
Crystal system	Monoclinic	
Space group	C 2	
Unit cell dimensions	a = 17.0788(6) Å	α= 90°.
	b = 5.5622(2) Å	β= 103.916(2)°.
	c = 18.6402(6) Å	γ = 90°.
Volume	1718.77(10) Å^3	
Z	4	
Density (calculated)	1.404 Mg/m^3	
Absorption coefficient	0.116 mm^{-1}	
F(000)	776	
Crystal size	0.400 x 0.100 x 0.080 mm^3	
Theta range for data collection	2.251 to 27.095°.	
Index ranges	-21<=h<=21, -7<=k<=7, -23<=l<=23	
Reflections collected	28816	
Independent reflections	3788 [R(int) = 0.0572]	
Completeness to theta = 25.000°	99.9 %	
Absorption correction	Semi-empirical from equivalents	
Max. and min. transmission	0.9705 and 0.7926	
Refinement method	Full-matrix least-squares on F^2	
Data / restraints / parameters	3788 / 3 / 257	
Goodness-of-fit on F^2	1.026	
Final R indices [I>2sigma(I)]	R1 = 0.0383, wR2 = 0.0856	
R indices (all data)	R1 = 0.0439, wR2 = 0.0893	
Absolute structure parameter	-0.2(12)	
Extinction coefficient	0.0022(8)	
Largest diff. peak and hole	0.302 and -0.305 e.Å^{-3}	

Table S2: Atomic coordinates (× 10^4) and equivalent isotropic displacement parameters (Å^2 × 10^3) for **4**. U(eq) is defined as one third of the trace of the orthogonalized U^{ij} tensor.

	x	y	z	U(eq)
O(3)	3073(1)	2586(3)	7530(1)	21(1)
O(4)	3470(1)	8214(3)	8734(1)	18(1)
O(5)	2434(1)	6166(3)	7326(1)	19(1)
O(8)	3867(1)	6637(3)	6160(1)	25(1)
O(9)	3160(1)	10114(3)	5858(1)	24(1)
O(10)	5733(1)	4160(4)	9115(1)	38(1)
N(1)	4504(1)	2537(4)	8667(1)	18(1)
C(1)	1643(2)	2793(5)	7507(2)	38(1)
C(2)	2471(2)	3991(4)	7731(1)	18(1)
C(3)	2706(1)	4442(5)	8562(1)	18(1)
C(4)	3535(2)	5652(4)	8812(1)	17(1)
C(5)	4126(1)	4781(4)	8360(1)	18(1)
C(6)	3699(2)	4339(4)	7554(1)	19(1)
C(7)	3212(1)	6470(5)	7160(1)	18(1)
C(8)	3097(2)	6296(5)	6325(1)	19(1)
C(9)	2580(2)	8314(5)	5907(2)	22(1)
C(10)	5284(2)	2393(5)	9032(1)	21(1)
C(11)	5584(2)	-41(5)	9315(2)	23(1)
C(13)	3853(2)	8863(5)	5764(2)	22(1)
C(14)	3793(2)	8310(6)	4958(2)	31(1)
C(15)	4580(2)	10334(5)	6119(2)	31(1)
O(1)	1052(2)	3668(6)	7731(2)	37(1)
O(12)	1603(2)	908(5)	7141(2)	35(1)
C(12)	856(2)	-445(9)	6990(3)	46(1)
O(1')	1471(4)	2577(18)	6689(5)	37(1)
O(12')	1527(4)	1182(15)	7800(5)	35(1)
C(12')	837(8)	-360(30)	7474(10)	46(1)
O(13)	7352(1)	4223(4)	9628(1)	25(1)

Table S3: Bond lengths (Å) and angles (°) for **4**.

O(3)-C(2)	1.412(3)
O(3)-C(6)	1.439(3)
O(4)-C(4)	1.434(3)
O(4)-H(4A)	0.75(3)
O(5)-C(2)	1.419(3)
O(5)-C(7)	1.445(3)
O(8)-C(8)	1.434(3)
O(8)-C(13)	1.439(3)
O(9)-C(13)	1.420(3)
O(9)-C(9)	1.426(3)
O(10)-C(10)	1.234(3)
N(1)-C(10)	1.345(3)
N(1)-C(5)	1.458(3)
N(1)-H(1A)	0.90(3)
C(1)-O(12')	1.092(9)
C(1)-O(12)	1.244(4)
C(1)-O(1)	1.277(4)
C(1)-O(1')	1.487(9)
C(1)-C(2)	1.529(4)
C(2)-C(3)	1.523(3)
C(3)-C(4)	1.536(3)
C(3)-H(3A)	0.9900
C(3)-H(3B)	0.9900
C(4)-C(5)	1.540(4)
C(4)-H(4)	1.0000
C(5)-C(6)	1.523(3)
C(5)-H(5)	1.0000
C(6)-C(7)	1.531(3)
C(6)-H(6)	1.0000
C(7)-C(8)	1.523(3)
C(7)-H(7)	1.0000
C(8)-C(9)	1.522(4)

C(8)-H(8)	1.0000
C(9)-H(9A)	0.9900
C(9)-H(9B)	0.9900
C(10)-C(11)	1.498(4)
C(11)-H(11A)	0.9800
C(11)-H(11B)	0.9800
C(11)-H(11C)	0.9800
C(13)-C(15)	1.502(4)
C(13)-C(14)	1.513(4)
C(14)-H(14A)	0.9800
C(14)-H(14B)	0.9800
C(14)-H(14C)	0.9800
C(15)-H(15A)	0.9800
C(15)-H(15B)	0.9800
C(15)-H(15C)	0.9800
O(12)-C(12)	1.448(5)
C(12)-H(12A)	0.9800
C(12)-H(12B)	0.9800
C(12)-H(12C)	0.9800
O(12')-C(12')	1.467(14)
C(12')-H(12D)	0.9800
C(12')-H(12E)	0.9800
C(12')-H(12F)	0.9800
O(13)-H(13A)	0.86(3)
O(13)-H(13B)	0.84(2)
C(2)-O(3)-C(6)	101.47(18)
C(4)-O(4)-H(4A)	109.5
C(2)-O(5)-C(7)	106.76(18)
C(8)-O(8)-C(13)	108.76(19)
C(13)-O(9)-C(9)	106.0(2)
C(10)-N(1)-C(5)	122.7(2)
C(10)-N(1)-H(1A)	116.6(19)
C(5)-N(1)-H(1A)	120.5(19)
O(12)-C(1)-O(1)	123.8(3)

O(12')-C(1)-O(1')	115.6(6)
O(12')-C(1)-C(2)	119.2(5)
O(12)-C(1)-C(2)	116.3(3)
O(1)-C(1)-C(2)	119.8(3)
O(1')-C(1)-C(2)	104.9(4)
O(3)-C(2)-O(5)	105.82(19)
O(3)-C(2)-C(3)	109.3(2)
O(5)-C(2)-C(3)	111.6(2)
O(3)-C(2)-C(1)	111.8(2)
O(5)-C(2)-C(1)	107.8(2)
C(3)-C(2)-C(1)	110.4(2)
C(2)-C(3)-C(4)	112.2(2)
C(2)-C(3)-H(3A)	109.2
C(4)-C(3)-H(3A)	109.2
C(2)-C(3)-H(3B)	109.2
C(4)-C(3)-H(3B)	109.2
H(3A)-C(3)-H(3B)	107.9
O(4)-C(4)-C(3)	111.2(2)
O(4)-C(4)-C(5)	107.6(2)
C(3)-C(4)-C(5)	112.0(2)
O(4)-C(4)-H(4)	108.7
C(3)-C(4)-H(4)	108.7
C(5)-C(4)-H(4)	108.7
N(1)-C(5)-C(6)	108.1(2)
N(1)-C(5)-C(4)	109.9(2)
C(6)-C(5)-C(4)	111.6(2)
N(1)-C(5)-H(5)	109.1
C(6)-C(5)-H(5)	109.1
C(4)-C(5)-H(5)	109.1
O(3)-C(6)-C(5)	108.4(2)
O(3)-C(6)-C(7)	101.31(19)
C(5)-C(6)-C(7)	115.1(2)
O(3)-C(6)-H(6)	110.5
C(5)-C(6)-H(6)	110.5
C(7)-C(6)-H(6)	110.5

O(5)-C(7)-C(8)	108.19(19)
O(5)-C(7)-C(6)	103.16(19)
C(8)-C(7)-C(6)	111.3(2)
O(5)-C(7)-H(7)	111.3
C(8)-C(7)-H(7)	111.3
C(6)-C(7)-H(7)	111.3
O(8)-C(8)-C(9)	103.93(19)
O(8)-C(8)-C(7)	108.14(19)
C(9)-C(8)-C(7)	113.2(2)
O(8)-C(8)-H(8)	110.5
C(9)-C(8)-H(8)	110.5
C(7)-C(8)-H(8)	110.5
O(9)-C(9)-C(8)	103.0(2)
O(9)-C(9)-H(9A)	111.2
C(8)-C(9)-H(9A)	111.2
O(9)-C(9)-H(9B)	111.2
C(8)-C(9)-H(9B)	111.2
H(9A)-C(9)-H(9B)	109.1
O(10)-C(10)-N(1)	121.8(2)
O(10)-C(10)-C(11)	121.8(2)
N(1)-C(10)-C(11)	116.4(2)
C(10)-C(11)-H(11A)	109.5
C(10)-C(11)-H(11B)	109.5
H(11A)-C(11)-H(11B)	109.5
C(10)-C(11)-H(11C)	109.5
H(11A)-C(11)-H(11C)	109.5
H(11B)-C(11)-H(11C)	109.5
O(9)-C(13)-O(8)	105.61(19)
O(9)-C(13)-C(15)	107.7(2)
O(8)-C(13)-C(15)	109.4(2)
O(9)-C(13)-C(14)	111.4(2)
O(8)-C(13)-C(14)	108.9(2)
C(15)-C(13)-C(14)	113.5(2)
C(13)-C(14)-H(14A)	109.5
C(13)-C(14)-H(14B)	109.5

H(14A)-C(14)-H(14B)	109.5
C(13)-C(14)-H(14C)	109.5
H(14A)-C(14)-H(14C)	109.5
H(14B)-C(14)-H(14C)	109.5
C(13)-C(15)-H(15A)	109.5
C(13)-C(15)-H(15B)	109.5
H(15A)-C(15)-H(15B)	109.5
C(13)-C(15)-H(15C)	109.5
H(15A)-C(15)-H(15C)	109.5
H(15B)-C(15)-H(15C)	109.5
C(1)-O(12)-C(12)	118.3(3)
O(12)-C(12)-H(12A)	109.5
O(12)-C(12)-H(12B)	109.5
H(12A)-C(12)-H(12B)	109.5
O(12)-C(12)-H(12C)	109.5
H(12A)-C(12)-H(12C)	109.5
H(12B)-C(12)-H(12C)	109.5
C(1)-O(12')-C(12')	119.8(11)
O(12')-C(12')-H(12D)	109.5
O(12')-C(12')-H(12E)	109.5
H(12D)-C(12')-H(12E)	109.5
O(12')-C(12')-H(12F)	109.5
H(12D)-C(12')-H(12F)	109.5
H(12E)-C(12')-H(12F)	109.5
H(13A)-O(13)-H(13B)	107(4)

Symmetry transformations used to generate equivalent atoms: n/a

Table S4: Anisotropic displacement parameters (Å^2 × 10^3) for **4**. The anisotropic displacement factor exponent takes the form $-2\pi^2(h^2 \times a \times 2U^{11} + ... + 2h \times k \times a \times b \times U^{12})$.

	U^{11}	U^{22}	U^{33}	U^{23}	U^{13}	U^{12}
O(3)	22(1)	15(1)	22(1)	-2(1)	-1(1)	-1(1)
O(4)	16(1)	13(1)	25(1)	-2(1)	5(1)	0(1)
O(5)	20(1)	17(1)	21(1)	3(1)	5(1)	2(1)
O(8)	24(1)	23(1)	30(1)	6(1)	10(1)	5(1)
O(9)	23(1)	21(1)	29(1)	4(1)	11(1)	4(1)
O(10)	19(1)	20(1)	64(2)	3(1)	-11(1)	-3(1)
N(1)	15(1)	12(1)	24(1)	0(1)	0(1)	1(1)
C(1)	31(2)	24(2)	45(2)	12(1)	-16(1)	-10(1)
C(2)	17(1)	15(1)	21(1)	2(1)	1(1)	0(1)
C(3)	16(1)	18(1)	20(1)	3(1)	1(1)	-3(1)
C(4)	16(1)	15(1)	17(1)	0(1)	-1(1)	-1(1)
C(5)	16(1)	15(1)	22(1)	1(1)	1(1)	0(1)
C(6)	20(1)	17(1)	18(1)	-1(1)	3(1)	1(1)
C(7)	17(1)	18(1)	20(1)	-1(1)	4(1)	0(1)
C(8)	19(1)	19(1)	20(1)	0(1)	5(1)	-1(1)
C(9)	20(1)	26(1)	22(1)	8(1)	6(1)	1(1)
C(10)	18(1)	18(1)	25(1)	-2(1)	-1(1)	0(1)
C(11)	19(1)	20(1)	29(1)	0(1)	0(1)	1(1)
C(13)	20(1)	22(1)	25(1)	2(1)	8(1)	4(1)
C(14)	27(1)	40(2)	27(2)	0(1)	11(1)	6(1)
C(15)	25(1)	25(2)	42(2)	-5(1)	9(1)	-2(1)
O(1)	13(1)	56(2)	43(2)	-22(2)	11(1)	-7(1)
O(12)	17(1)	26(1)	61(2)	-18(1)	10(1)	-8(1)
C(12)	14(2)	30(2)	89(4)	-22(3)	4(2)	-7(2)
O(1')	13(1)	56(2)	43(2)	-22(2)	11(1)	-7(1)
O(12')	17(1)	26(1)	61(2)	-18(1)	10(1)	-8(1)
C(12')	14(2)	30(2)	89(4)	-22(3)	4(2)	-7(2)
O(13)	15(1)	36(1)	23(1)	-2(1)	2(1)	3(1)
